# Effects of total glucosides of paeony on serum inflammatory cytokines in animal models of rheumatoid arthritis: a systematic review and meta-analysis

**DOI:** 10.3389/fphar.2024.1349259

**Published:** 2024-03-25

**Authors:** Mengdi He, Zhipeng Hu, Maoyi Yang

**Affiliations:** Hospital of Chengdu University of Traditional Chinese Medicine, Chengdu, China

**Keywords:** total glucosides of paeony, rheumatoid arthritis, animal models, inflammatory cytokines, meta-analysis

## Abstract

**Background:** Rheumatoid arthritis (RA) is an autoimmune disease characterized by chronic synovitis of the affected joints. Total glucosides of paeony (TGP) capsules have been widely used clinically for the treatment of RA with good efficacy and safety. However, its effect on inflammatory cytokines remains unclear.

**Objectives:** This study aimed to summarize the effect of TGP on the expression level of serum inflammatory cytokines in RA animal models and its potential mechanisms.

**Methods:** Six databases were searched up to 14 August 2023, relevant animal experiment studies were screened, data were extracted, and the SYRCLE animal experiment bias risk assessment tool was used for risk assessment.

**Results:** A total of 24 studies were included, including 581 animals. Results showed that compared with the model control group, TGP decreased the levels of TNF-α, IL-1β, IL-6, and PGE2 and increased the levels of TGF-β1 after 1–2 weeks of intervention, decreased the levels of TNF-α, IL-1β, IL-6, IL-2, IL-17, IL-17α, IL-21, VEGF, IFN-γ and PGE2 and increased the levels of IL-10 and IL-4 after 3–4 weeks of intervention, decreased the levels of TNF-α, IL-6, IL-17α and increased the level of IL-10 after 8 weeks of intervention. There was no significant difference in the effects of TGP on the levels of IL-10, IL-17, and IFN-γ after 1–2 weeks of intervention and IL-1 and TGF-β1 after 3–4 weeks of intervention.

**Conclusion:** In summary, based on the existing studies, this study found that compared with the control group of the RA animal model, TGP can reduce the levels of serum pro-inflammatory cytokines such as TNF-α, IL-1β, and IL-6 and increase the levels of serum anti-inflammatory cytokines such as IL-10, exerting an anti-inflammatory effect by regulating and improving the levels of inflammatory cytokines, and thus alleviating the disease. Given the low quality of the included studies and the lack of sufficient evidence, more high-quality studies are still needed to validate the results of this study.

## 1 Introduction

Rheumatoid arthritis (RA) is an autoimmune disease characterized by chronic synovitis of the affected joints, which can lead to progressive cartilage and bone damage as well as disability (Smolen et al., 2016). The most prominent clinical feature of the disease is symmetric, polyarticular pain and swelling, mainly involving the hands, wrists, feet, and knees (Sparks, 2019). The prevalence of RA is about 0.5%–1% and it is higher in some populations. For example, the prevalence is higher in the Northern Hemisphere and urban areas than in the Southern Hemisphere and rural areas, which in women is 2–3 times higher than that in men globally (Smolen et al., 2016; Gravallese and Firestein, 2023). Interactions among cells within joint synovium are a key part of RA pathogenesis. Specifically, B cells, dendritic cells, and macrophages can present antigens to T cells to activate these cells and induce their differentiation, leading to the production of cytokines, which in turn activate other neighboring cells to produce more pro-inflammatory cytokines and factors (Gravallese and Firestein, 2023). Thus, as signaling mediators both among immune cells as well as between immune cells and tissue cells, cytokines (such as inflammatory cytokines) have an important role in the establishment and perpetuation of inflammation in RA (Lin et al., 2020).

Total glucosides of paeony (TGP) capsules, also known as Pafulin, extracted from the dry roots of the traditional Chinese medicine plant Paeonia lactiflora, have been developed as a drug for the treatment of RA and are widely used in the clinic in China (Jiang et al., 2020). A meta-analysis found that compared with methotrexate and leflunomide, TGP combined with the above therapy for RA significantly decreased the disease activity score in 28 joints and the occurrence of liver dysfunction, leukopenia, diarrhea, nausea, and vomiting, which has a favorable efficacy and safety (Yang et al., 2023). Existing studies have shown that TGP and its main active ingredient, paeoniflorin, can exert anti-inflammatory and immunomodulatory effects by regulating the function and activation of immune cells, reducing the production of inflammatory mediators, and restoring abnormal signaling pathways (Zhang and Wei, 2020).

Animal models of RA are used to study the pathogenesis of RA and to identify therapeutic targets of drugs (McNamee et al., 2015). They are classified into induced and genetic models, and among the former, adjuvant-induced arthritis (AIA) and collagen-induced arthritis (CIA) models are most commonly used because of the advantages of simple operation and fast modeling (Choudhary et al., 2018). The AIA model mainly shows a T cell-mediated cellular immune response, whereas the CIA model shows both cellular and humoral immunity co-mediated by T and B cells, and the immunological characteristics are more similar to those of humans (Hegen et al., 2008; Asquith et al., 2009).

Although there are many studies on the effects of TGP on inflammatory cytokines, the outcomes reported vary greatly, resulting in no unified understanding, which is the starting point of this study.

Given the above-mentioned situation, this study aims to conduct a systematic review and meta-analysis of relevant *in vivo* animal experiments, to explore the effects of TGP on the expression level of inflammatory cytokines in serum during the pathogenesis of RA. In addition, potential mechanisms of TGP on RA in the included studies were summarized, to provide some basis for clinical research.

## 2 Materials and methods

### 2.1 Databases and search strategies

Chinese databases of China National Knowledge Infrastructure (CNKI), Wanfang, VIP, and English databases of PubMed, Embase, and Cochrane Library were retrieved from inception to 14 August 2023. The basic search framework was: (“total glucosides of paeony”) and (“rheumatoid arthritis” or “experimental arthritis”). Moreover, retrieve references cited by the included studies and include them if they met the inclusion criteria. The previous step was repeated for that included reference until no references met the inclusion criteria. This is to minimize the rate of missed detections. Detailed search strategies can be found in Supplementary Material S1.

### 2.2 Inclusion criteria

1) Study Types: *In vivo* animal experiments.
2) Participants: No limitations on animal model type, species, strain, sex, weight, or age.
3) Interventions: The experimental group is given TGP, and the control group is given drug solvents or no treatment. The intervention time and manufacturer of TGP are not limited. In relevant studies, the drug administration route was mainly gavage (i.g.), and a few were intraperitoneal injections. There are two reasons for limiting the route of administration to gavage. First, intraperitoneal injection has no gastrointestinal first-pass metabolism, its bioavailability of drugs is higher than that of the gavage. So there is a fundamental difference between these two routes of administration. Second, clinical administration of TGP is oral, which is more closely related to gavage. Thus, gavage has a better clinical representation. The intervention dose of TGP is limited from the clinical reality, the standard dose used in adult patients with RA (70 kg) is 0.6 g Tid (Yang et al., 2023). Equivalent doses are converted according to the body surface area coefficients of different experimental animals and humans (The formula is shown below.), such as the equivalent dose for rats (200 g) is 163 mg·kg-1·day-1, and the equivalent dose for mice (20 g) is 234 mg·kg-1·day-1 (Huang et al., 2004). Based on the above criteria, the intervention dose is limited to not exceeding two times the equivalent dose, for example, the dose of rats is less than or equal to 326 mg·kg-1·day-1, and the dose of mice is less than or equal to 468 mg·kg-1·day-1.
$$ED=HD\times \frac{K_{a}}{K_{h}}\times {\left(\frac{W_{h}}{W_{a}}\right)}^{\frac{1}{3}}$$
  In the formula, ED is the equivalent dose for animals (mg·kg-1·day-1) and HD is the standard dose for humans (assuming a constant human body weight of 70 kg, HD = 1800/70 mg·kg-1·day-1). K_a_ and K_h_ are constants, representing the body size coefficients of animals and humans respectively. K_a_ is 0.06 in mice and 0.09 in rats. K_h_ is 0.1. W_a_ and W_h_ are the animal and human body weight (kg). The calculation results are retained in whole numbers.
4) Outcomes: Serum inflammatory cytokines (such as TNF-α, IL-1, IL-2, IL-1β, IL-4, IL-6, IL-10, IL-17, IL-17α, IL-21, VEGF, IFN-γ, PGE2, TGF-β1). The main outcomes are TNF-α, IL-1β, IL-6 and IL-10. As long as any outcome is reported in the study, it can be included. If the study reports multiple time points, the results are all extracted.

### 2.3 Exclusion criteria

(1) The same study with duplicate publications (retaining the one with more complete data); (2) Reviews; (3) Clinical studies and case reports; (4) Conference literature, scientific and technological achievements, and guidelines; (5) Cell experiments; (6) Interventions using paeoniflorin or other components of TGP; (7) Studies that do not report data.

### 2.4 Data extraction and management

Two researchers (Mengdi He and Zhipeng Hu) independently screened the literature by reading the title, abstract, and full text to obtain the literature that met the inclusion criteria. Disagreements were resolved by consulting a third researcher (Maoyi Yang). Two researchers (Mengdi He and Zhipeng Hu) independently extracted data in Excel, including literature author, publication year, country, sex ratio, animal species and strains, age, body weight, equivalent dose, type of animal model, sample size, co-intervention, interventions in experimental *versus* control groups, route of administration, intervention time, manufacturer of TGP, paeoniflorin content, measurement specimens, outcomes and their detection methods. Then cross-check to reduce the error rate. Outcome data were recorded as mean and standard deviation. If the literature provided standard error, the data were transformed. If the literature presented data only in the form of images, data were extracted three times using WebPlotDigitizer and averaged. If the literature lacked relevant data, Mengdi He attempted to contact the authors and excluded the literature when no response resulted in incomplete key data.

### 2.5 Risk of bias assessment

Two investigators (Mengdi He and Zhipeng Hu) used the SYRCLE tool to assess the risk of bias in animal experiments (Zeng et al., 2015), which included the following 10 perspectives: (1) sequence generation; (2) baseline characteristics; (3) allocation concealment; (4) random housing; (5) blinding of animal keepers and investigators; (6) assessment of randomization outcomes; (7) blinding of outcome evaluators; (8) reporting of incomplete data; (9) selective reporting of outcomes; (10) other sources of bias. Each included study was classified as Yes (low risk of bias), No (high risk of bias), or Uncertain (uncertain risk of bias). Disagreements were resolved by consulting a third researcher (Maoyi Yang).

### 2.6 Statistical methods

Meta-analysis was performed using RevMan 5.4 provided by the Cochrane Collaboration and Stata 12.0 software. When multiple intervention groups were included in a study, the common control group was evenly divided into several small sample groups (Control group sample size divided by the number of doses in the intervention group), and then all pairwise comparison combinations were included in the meta-analysis to avoid artificially expanding the sample size (Higgins et al., 2023). For example, in a study with three doses of TGP intervention groups (n = 10) and a control group (n = 10), the sample size of the control group was split into 3, 3, and 4, which were combined with the intervention groups into the 3–10, 3–10, 4–10 comparison combinations.

All outcomes were continuous variables. If the effect size units and measurement methods were the same among studies, effect sizes were represented by weighted mean difference (WMD) and 95% confidence interval (CI). Conversely, standardized mean difference (SMD) and 95% CI were used. Combined effect sizes were considered statistically significant at *p* < 0.05. *I* ^*2*^ and Q-test were used to assess the heterogeneity of the studies. If *I* ^*2*^ ≤ 50% or *p* ≥ 0.1, the heterogeneity among the studies was small, and the fixed-effects model was used. If *I* ^*2*^ > 50% or *p* < 0.1, the heterogeneity among the studies was large, and leave-one-out sensitivity analysis was performed first to explore the source of heterogeneity and judge the stability of the results. If the source could not be found, the random-effects model was used and subgroup analysis was performed.

Subgroup analysis was performed from five perspectives: dose and manufacturer of TGP, model type (CIA or AIA), animal species (such as rat or mouse), and animal strain [such as Wistar or Sprague Dawley (SD) rat, DBA/1 mouse]. Subgroup analysis for each perspective was carried out when the number of subgroups was less than or equal to 3. The dose subgroup analysis was based on 1/2 of the equivalent dose, the equivalent dose, and two times the equivalent dose as the division points (Tang, 2020). Specifically, in rats (200 g), 0 < low dose (LD) group ≤81.5 mg·kg-1·day-1, 81.5 mg·kg-1·day-1< medium dose (MD) group ≤163 mg·kg-1·day-1, 163 mg·kg-1·day-1 < high dose (HD) group ≤326 mg·kg-1·day-1; in mice (20 g), 0< LD ≤ 117 mg·kg-1·day-1, 117 mg·kg-1·day-1 < MD ≤ 234 mg·kg-1·day-1, 234 mg·kg-1·day-1 < HD ≤ 468 mg·kg-1·day-1. Although the equivalent doses for 200 g rats and 20 g mice obtained by conversion from the standard dose for a 70 kg human are acceptable within a certain body weight range, large conversion errors occur when the actual body weights of the rats or mice are outside that range (Xia et al., 2021). Therefore, in the dose subgroup analysis, we convert the equivalent dose and adjust the low, medium, and high dose ranges one by one according to the mean of the actual body weight of the animals in each study.

When there were ten or more comparison groups for the same outcome, publication bias was assessed by plotting a funnel plot or performing an Egger’s test (with *p* < 0.05 as the presence of publication bias).

## 3 Results

### 3.1 Literature screening

**FIGURE 1:**
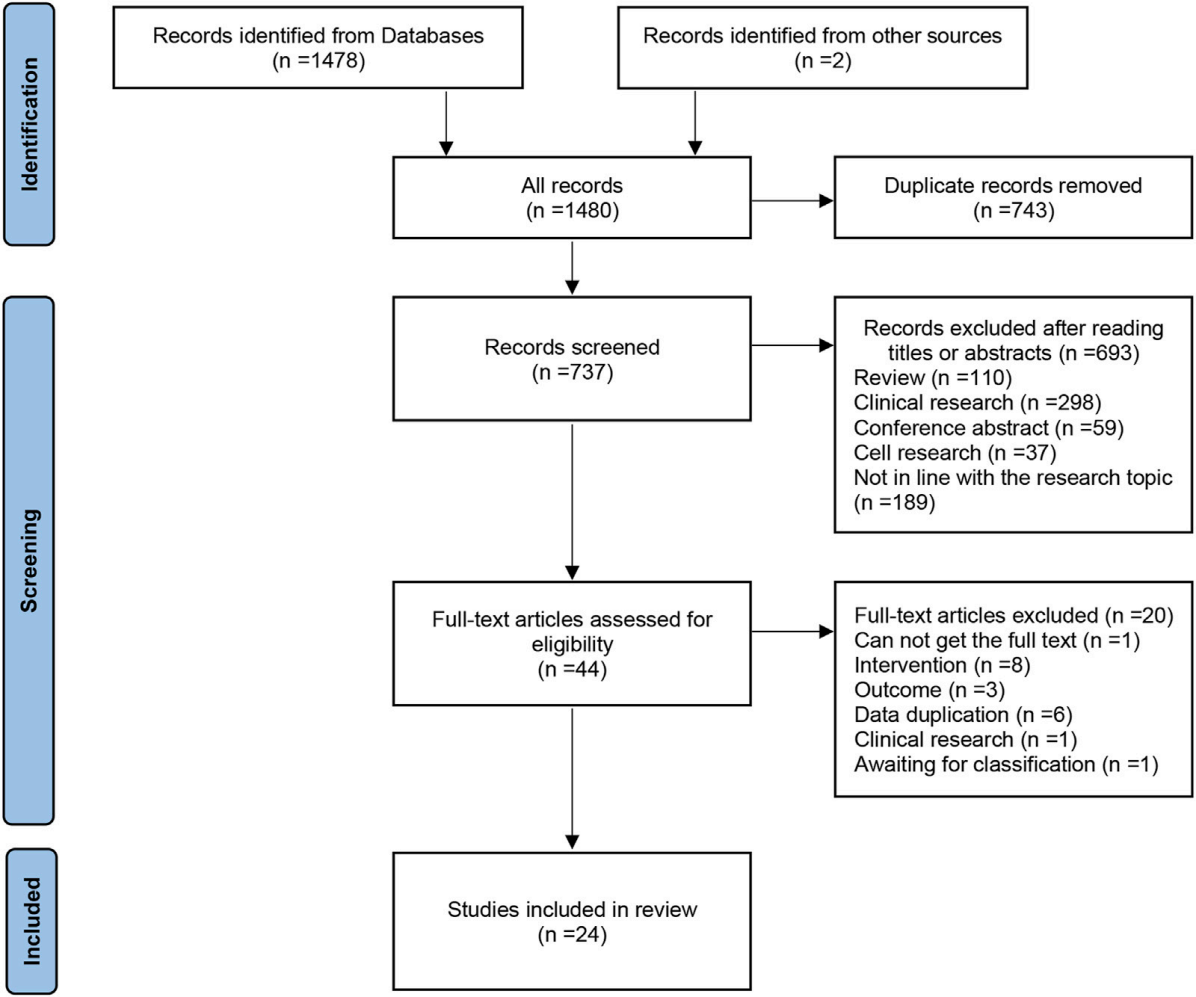
The flow chart of literature screening.

A total of 1478 studies were retrieved from the databases (CNKI, 545; Wanfang, 462; VIP, 295; PubMed, 86; Embase, 77; Cochrane Library, 13). First, 743 studies were excluded due to duplication, and then 693 studies were excluded by reading the title and abstract. 20 studies were excluded by reading the full text and one of them was categorized as unclassified because the measurement specimen was unknown (Fu et al., 2022), and then 22 studies were included. By searching the references of the included studies, two additional studies were included (Chang et al., 2016; Asenso et al., 2019), and finally, 24 studies were included (Asenso et al., 2019; Chang et al., 2016; Jia, 2015; Li et al., 2019; Li, 2012; Li et al., 2011; Li et al., 2010; Liu et al., 2010; Liu et al., 2012; Peng et al., 2012; Qin, 2008; Qu et al., 2022; Shen et al., 2019; Wang et al., 2013a; Wang et al., 2013b; Wang, 2019; Xie, 2019; Yan et al., 2021; Yao, 2007; Yuan, 2013; Zhou et al., 2019; Zhou et al., 2018; Zhou et al., 2012; Zhou et al., 2009). It should be noted that during the screening process, regarding characteristics and outcome data, study (Chang et al., 2012a) is similar to study (Li et al., 2010), and study (Chang et al., 2012b) is similar to study (Li et al., 2011), and there was a great possibility of duplicated reporting. So Mengdi He tried to contact the authors for more detailed information, but the authors did not respond, and out of an abundance of caution, we excluded two studies by Jingzhi Chang (Chang et al., 2012a; Chang et al., 2012b). The specific literature screening flow chart is shown in Figure 1.

### 3.2 Basic characteristics of the included studies

The meta-analysis included a total of 41 independent comparison groups, 581 experimental and control animals, all of which were rodents, including 510 rats (392 SD rats, 98 Wistar rats, 20 Lewis rats) and 71 mice (31 DBA/1 mice, 40 C57BL/6J mice). Fifteen studies used SD rats, five studies used Wistar rats, one study used Lewis rats, two studies used DBA/1 mice, and one study used C57BL/6J mice. In terms of model types, 12 studies used the AIA model, and 12 studies used the CIA model.

The dose range of TGP was 25–370 mg·kg-1·day-1, with six studies involving three doses, one study involving two doses, and 17 studies involving one dose. The intervention time of TGP ranged from 1 to 8 weeks, with 23 studies for 1–4 weeks and one study for 8 weeks. Regarding the reported time points of outcomes, only two studies reported multiple time points. TGP originated from six different manufacturers: (1) Institute of Clinical Pharmacology, Anhui Medical University. (2) Anhui Bozhou Chinese herbal medicine market (self-made purification). (3) Ningbo Lihua Pharmaceutical Co., Ltd. (4) Shanxi Weikangtang Chinese Herbal Pieces Co., Ltd. (5) Sanjiu Pharmaceutical Co., Ltd. (6) Ningbo Yicuijian Biological Technology Co., Ltd. Among them, the first two are self-made purification, and the last four are proprietary Chinese medicine. The specific basic characteristics are shown in Table 1.

**TABLE 1:** Characteristics of the included studies.

Study	Country	Animal type	Female and male proportion	Age (week)	Weight (g)	Equivalent dose (mg·kg-1·day-1)	Animal model	Sample size (EG/CG)	Co-intervention	Intervention (EG, TGP, mg·kg-1·day-1)	Intervention (CG)
Jia Xiaoyi2015	China	SD rat	0/1	NR	150 ± 20 (150)	180	AIA	10/10	CFA	50	NR
Li Xingxing2012	China	SD rat	0/1	6–8	160 ± 15 (160)	176	AIA	8/8	CFA	150	H2O
Li Yichuan2011	China	SD rat	1/1	NR	180 ± 20 (180)	169	CIA	10/10/10/10	CII, CFA	25/50/100	NS
Li Yichuan2010	China	SD rat	1/1	NR	180 ± 20 (180)	169	CIA	10/10/10/10	CII, CFA	25/50/100	NS
Liu Guoling2010	China	SD rat	1/1	NR	about 180 (180)	169	CIA	10/10/10/10	CII, CFA	25/50/100	NR
Liu Guoling2012	China	SD rat	1/1	NR	about 180 (180)	169	CIA	10/10/10/10	CII, CFA	25/50/100	NR
Qin Ce2008	China	Wistar rat	0/1	8–12	140–160 (150)	180	AIA	10/10/10	CFA	50/300	H2O
Wang Gang 2013 (1)	China	SD rat	1/1	NR	200 ± 20 (200)	163	CIA	8/8	CII, CFA	108	H2O
Wang Gang 2013 (2)	China	SD rat	1/1	NR	200 ± 20 (200)	163	CIA	24/24	CII, CFA	108	H2O
Wang Qian2019	China	SD rat	NR	NR	180 ± 20 (180)	169	AIA	10/10	CFA	126	NS
Xie Yunfei2019	China	SD rat	0/1	NR	200 ± 20 (200)	163	AIA	10/10	CFA	200	NS
Yan Lijun2021	China	SD rat	0/1	10–12	200–230 (215)	159	CIA	10/10/10/10	CII, CFA	25/50/100	NS
Zhou Wentao2018	China	Wistar rat	1/0	5–9	180–220 (200)	163	CIA	10/10	CII, CFA	160	NS
Zhou Xiaotao2012	China	Wistar rat	0/1	NR	180–220 (200)	163	AIA	8/8	CFA	160	NS
Zhou Xiaotao2009	China	Wistar rat	0/1	NR	180–220 (200)	163	AIA	8/8	CFA	160	NS
Peng Cheng2012	China	SD rat	1/0	NR	120–140 (130)	188	CIA	8/8	CII, CFA	180	NR
Qu Biao2022	China	SD rat	NR	NR	100–120 (110)	199	AIA	6/6	CFA	150	PBS
Yao Qi2007	China	Wistar rat	0/1	NR	160 ± 20 (160)	176	AIA	8/8	CFA	65	NR
Zhou Rui2019	China	SD rat	0/1	NR	160 ± 20 (160)	176	AIA	6/6	CFA	125	NS
Li Hui2019	China	DBA/1 mice	0/1	8	NR (20)	234	CIA	8/8	CII, CFA	360	0.5% CMC-Na
Shen Weixing2019	China	DBA/1 mice	0/1	8	NR (20)	234	CIA	7/8	CII, CFA	370	0.5% CMC-Na
Yuan Jun2013	China	C57BL/6J mice	0/1	NR	20 ± 2 (20)	234	CIA	10/10/10/10	CII, CFA	35/70/140	NR
James Asenso2019	China	SD rat	0/1	4	185 ± 15 (185)	167	AIA	6/6	CFA	50	0.5% CMC-Na
Chang Yan2016	China	Lewis rat	0/1	NR	150–180 (165)	174	AIA	10/10	CFA	50	0.5% CMC-Na

Study	The route of administration	Intervention time(d)	Manufacturer of TGP	Paeoniflorin content	Specimen	Detection method	Outcomes
Jia Xiaoyi2015	i.g	14	Institute of Clinical Pharmacology, Anhui Medical University	NR	serum	ELISA	TNF-α, PGE-2
Li Xingxing2012	i.g	28	Ningbo Lihua Pharmaceutical Co., Ltd	NR	serum	NR	TNF-α, IL-1
Li Yichuan2011	i.g	24	Institute of Clinical Pharmacology, Anhui Medical University	NR	serum	ELISA	IL-1β, VEGF
Li Yichuan2010	i.g	21	Institute of Clinical Pharmacology, Anhui Medical University	NR	serum	ELISA	TNF-α, IL-1β, IL-4, IL-10
Liu Guoling2010	i.g	21	Institute of Clinical Pharmacology, Anhui Medical University	NR	serum	ELISA	TNF-α, IL-1β, IL-10
Liu Guoling2012	i.g	21	Institute of Clinical Pharmacology, Anhui Medical University	NR	serum	ELISA	IL-17, VEGF
Qin Ce2008	i.g	14, 28	Ningbo Lihua Pharmaceutical Co., Ltd	NR	plasma	ELISA	IL-1β, IFN-γ
Wang Gang 2013 (1)	i.g	21	Ningbo Lihua Pharmaceutical Co., Ltd	≥34.7%	serum	ELISA	IL-4, INF-γ
Wang Gang 2013 (2)	i.g	7, 14, 21	Ningbo Lihua Pharmaceutical Co., Ltd	≥34.7%	serum	ELISA	TNF-α
Wang Qian2019	i.g	28	Shanxi Weikangtang Chinese Herbal Pieces Co., Ltd	NR	serum	ELISA	TNF-α
Xie Yunfei2019	i.g	14	Anhui Bozhou Chinese herbal medicine market (self-made purification)	81.6%	serum	ELISA	TNF-α, IL-1β, IL-6, IL-10, IFN-γ
Yan Lijun2021	i.g	21	Ningbo Lihua Pharmaceutical Co., Ltd	NR	serum	ELISA	TNF-α, IL-1β, IL-6
Zhou Wentao2018	i.g	25	Sanjiu Pharmaceutical Co., Ltd	NR	serum	ELISA	TNF-α, IL-17α
Zhou Xiaotao2012	i.g	25	Sanjiu Pharmaceutical Co., Ltd	NR	serum	ELISA	TNF-α
Zhou Xiaotao2009	i.g	25	Sanjiu Pharmaceutical Co., Ltd	NR	serum	ELISA	TNF-α
Peng Cheng2012	i.g	7	Ningbo Lihua Pharmaceutical Co., Ltd	NR	serum	ELISA	TNF-a, IL-1β
Qu Biao2022	i.g	21	Ningbo Yicuijian Biological Technology Co., Ltd	45.29%	serum	ELISA	TNF-α, IL-1β, IL-6
Yao Qi2007	i.g	7	Ningbo Lihua Pharmaceutical Co., Ltd	NR	serum	ELISA	TNF-α, IL-1β
Zhou Rui2019	i.g	23	Ningbo Lihua Pharmaceutical Co., Ltd	NR	serum	ELISA	TNF-α, IL-1, IL-6, PGE2
Li Hui2019	i.g	21	Ningbo Lihua Pharmaceutical Co., Ltd	about 47%	serum	ELISA	TNF-α, IL-6, IL-10(NR), IL-21
Shen Weixing2019	i.g	56	Ningbo Lihua Pharmaceutical Co., Ltd	NR	serum	ELISA	TNF-α, IL-6, IL-10, IL-17α
Yuan Jun2013	i.g	21	Institute of Clinical Pharmacology, Anhui Medical University	NR	serum	ELISA	IL-2, IL-4, IL-10, IL-17, TGF-β1
James Asenso2019	i.g	21	Institute of Clinical Pharmacology, Anhui Medical University	NR	serum	ELISA	TNF-α, IL-6, IL-17
Chang Yan2016	i.g	13	Institute of Clinical Pharmacology, Anhui Medical University	>90%	serum	ELISA	TNF-α, IL-1β, IL-6, IL-17, TGF-β1

Note: SD, Sprague-Dawley; CIA, collagen-induced arthritis; AIA, adjuvant-induced arthritis; CFA, complete freund’s adjuvant; EG, experimental group; CG, control group; NS, normal saline; NR, not reported; TGP, total glucosides of paeony; CII, type II collagen; CMC-Na, sodium carboxymethyl cellulose; PBS, phosphate buffered saline; i.g., gavage; TNF-α, tumor necrosis factor-α; IL-1β, interleukin-1β; IL-6, interleukin-6; IL-10, interleukin-10; IL-1, interleukin-1; IL-2, interleukin-2; IL-4, interleukin-4; IL-17, interleukin-17; IL-17α, interleukin-17α; IL-21, interleukin-21; VEGF, vascular endothelial growth factor; IFN-γ, interferon-γ; PGE2, prostaglandin E2; TGF-β1, transforming growth factor-β1.

### 3.3 Risk of bias assessment

**FIGURE 2:**
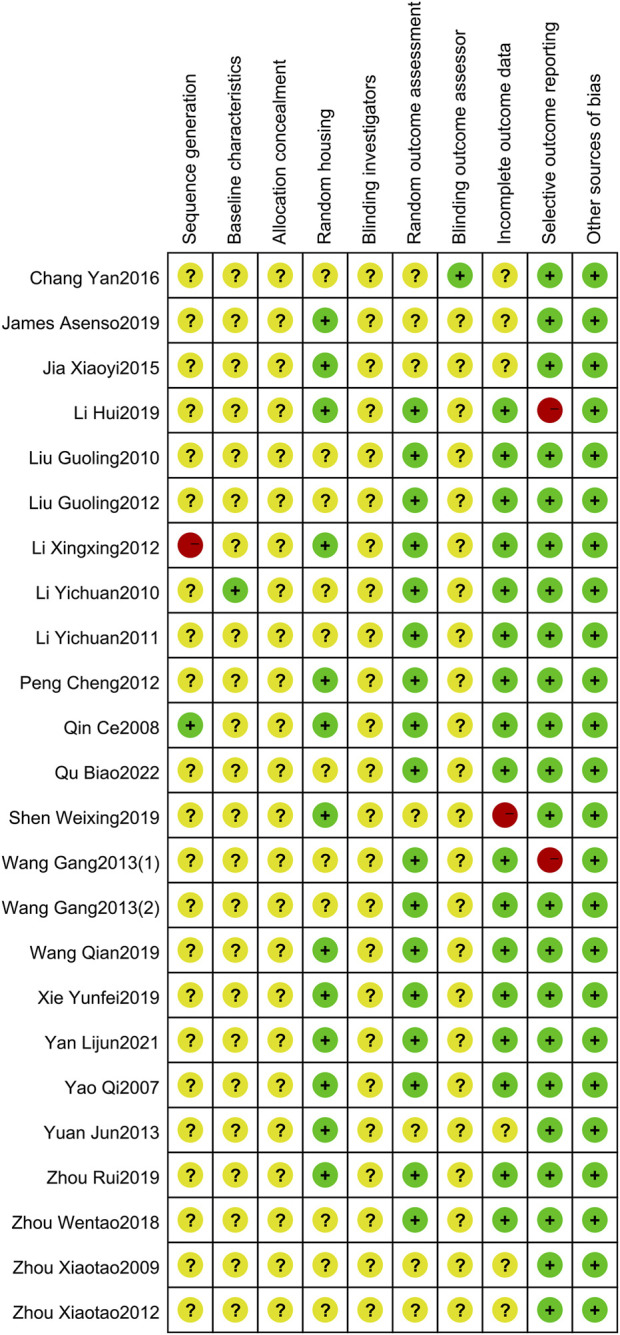
Risk of bias summary.

**FIGURE 3:**
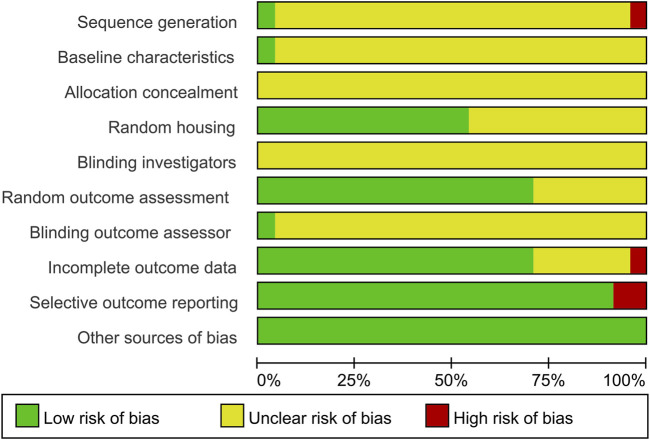
Risk of bias graph.

Only one study described the specific method of random sequence generation, one study had an incorrect method of sequence generation, and the rest mentioned randomization. The baseline characteristic of only one study was rated as low risk because it described serum cytokine levels after modeling. This phenomenon may be caused by the fact that existing studies were still accustomed to using outcomes such as paw swelling and arthritis index to measure baseline characteristics, and the topic of some studies was not that type of outcome, so it was not paid attention to before intervention of TGP. None of the studies described allocation concealment or blinding of animal keepers and investigators. Seven studies were rated as unclear risk in random outcome assessment. One study stated blinding of outcome evaluators. 13 studies mentioned randomization of animal placement. One study had incomplete data reporting. Two studies were rated as high risk for selective reporting of outcomes, one of which had multiple time points but reported only one, the other described measurement of serum IL-10 levels in the experimental method, but the results were not reported. All other sources of bias were low risk. The risk of bias summary and risk of bias graph are shown in Figure 2 and Figure 3.

### 3.4 Effects of TGP on RA

#### 3.4.1 TNF-α

1) TNF-α (Intervention time is 1–2 weeks).

**FIGURE 4:**
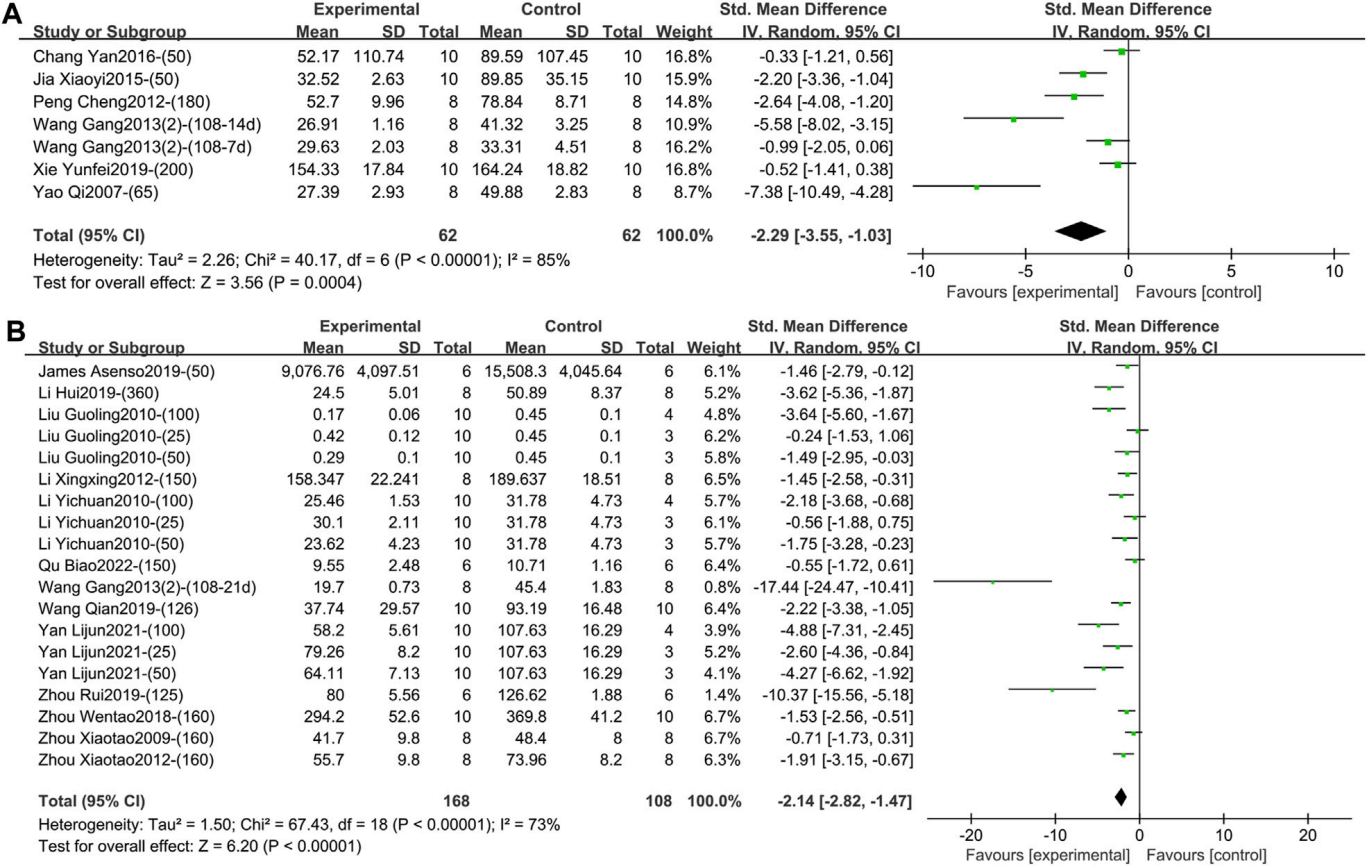
**(A)** Forest plot of TNF-α after 1–2 weeks of TGP intervention. **(B)** Forest plot of TNF-α after 3–4 weeks of TGP intervention.

Six studies including 124 animals reported the effect of TGP on serum TNF-α after 1–2 weeks of intervention (Chang et al., 2016; Jia, 2015; Peng et al., 2012; Wang et al., 2013b; Xie, 2019; Yao, 2007). The combined results showed that TGP could reduce TNF-α levels, but the heterogeneity between studies was high (SMD = −2.29, 95% CI [-3.55, −1.03], *p* = 0.0004, *I* ^*2*^ = 85%) (Figure 4A; Supplementary Material S2.1). Sensitivity analysis showed that the result was robust (Supplementary Material S2.2). Subgroup analyses showed that dose, model type, and TGP’s manufacturer were not sources of heterogeneity (*p* = 0.06, 0.54, 0.05) (Supplementary Material S2.3–2.5). There were significant statistical differences among the subgroups of animal strains (SD rat, Wistar rat, Lewis rat) (*p* < 0.0001) (Supplementary Material S2.6), but both the Wistar and Lewis strains only had one entry.

2) TNF-α (Intervention time is 3–4 weeks).

Thirteen studies containing 276 animals reported the effect of TGP on serum TNF-α after 3–4 weeks of intervention (Asenso et al., 2019; Li et al., 2019; Li, 2012; Li et al., 2010; Liu et al., 2010; Qu et al., 2022; Wang et al., 2013b; Wang, 2019; Yan et al., 2021; Zhou et al., 2019; Zhou et al., 2018; Zhou et al., 2012; Zhou et al., 2009). TGP reduced TNF-α levels compared to model controls with high inter-study heterogeneity (SMD = −2.14, 95% CI [-2.82, −1.47], *p* < 0.00001, *I* ^*2*^ = 73%) (Figure 4B; Supplementary Material S2.7). Sensitivity analysis indicated that the result was robust (Supplementary Material S2.8). Subsequent subgroup analyses were performed, and dose, model type, and animal species were not sources of heterogeneity (*p* = 0.07, 0.16, 0.10) (Supplementary Material S2.9–2.11). Subgroup analysis based on different animal strains (SD rat, Wistar rat, DBA/1 mice) was statistically different (*p* = 0.03), and the heterogeneity in the first two groups was 76% and 17%, respectively (Supplementary Material S2.12). In addition, the funnel plot (Supplementary Material S2.13) and Egger’s test (*p* < 0.001) indicated publication bias.

3) TNF-α (Intervention time is 8 weeks).

One study reported the effect of TGP on serum TNF-α after 8 weeks of intervention (Shen et al., 2019). Only descriptive analysis was performed, TGP reduced TNF-α levels compared with the model control group (SMD = −4.43, 95% CI [-6.53, −2.32], *p* < 0.0001) (Supplementary Material S2.14).

#### 3.4.2 IL-1β

1) IL-1β (Intervention time is 1–2 weeks).

**FIGURE 5:**
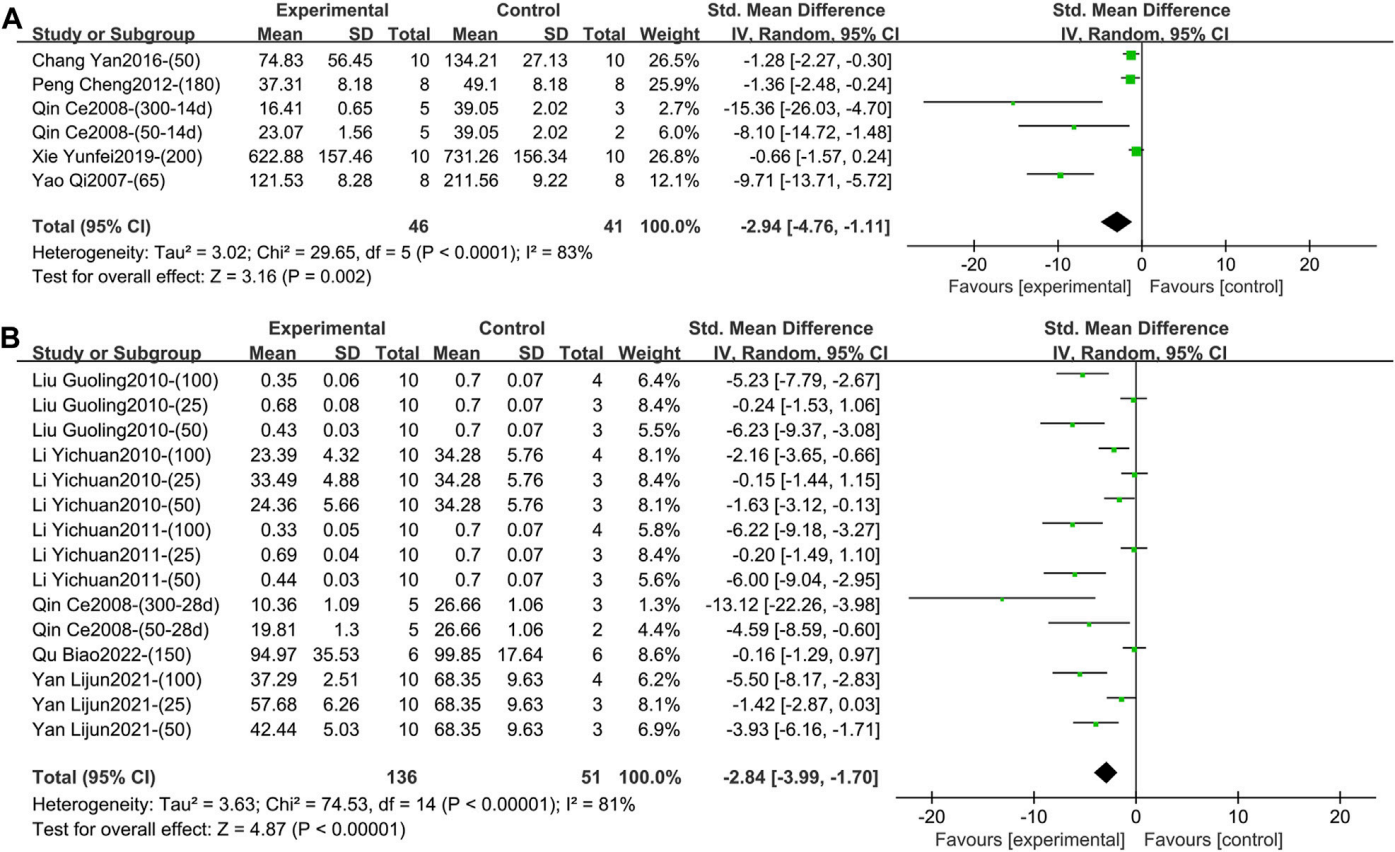
**(A)** Forest plot of IL-1β after 1–2 weeks of TGP intervention. **(B)** Forest plot of IL-1β after 3–4 weeks of TGP intervention.

Five studies containing 87 animals reported the effect of TGP intervention for 1–2 weeks on serum IL-1β (Yao, 2007; Qin, 2008; Peng et al., 2012; Chang et al., 2016; Xie, 2019). The combined results showed that TGP reduced IL-1β levels, but heterogeneity between studies was high (SMD = −2.94, 95% CI [-4.76, −1.11], *p* = 0.002, *I* ^*2*^ = 83%) (Figure 5A; Supplementary Material S2.15). Sensitivity analysis indicated that the result was robust (Supplementary Material S2.16). Subgroup analyses based on dose, model type, and TGP’s manufacturer indicated that these perspectives were not sources of heterogeneity (*p* = 0.29, 0.06, 0.08) (Supplementary Material S2.17–S2.19). Animal strain (SD rat, Wistar rat, Lewis rat) was a source of heterogeneity (*p* < 0.00001), and the heterogeneity disappeared in the subgroups of SD rat and Wistar rat (*I* ^*2*^ = 0) (Supplementary Material S2.20).

2) IL-1β (Intervention time is 3–4 weeks).

Six studies involving 187 animals reported the effect of TGP intervention for 3–4 weeks on serum IL-1β (Qin, 2008; Li et al., 2010; Liu et al., 2010; Li et al., 2011; Yan et al., 2021; Qu et al., 2022). The combined results showed that TGP significantly reduced IL-1β levels, while heterogeneity between studies was large (SMD = −2.84, 95% CI [-3.99, −1.70], *p* < 0.00001, *I* ^*2*^ = 81%) (Figure 5B; Supplementary Material S2.21). Sensitivity analysis showed that the result was robust (Supplementary Material S2.22). Subgroup analyses indicated that model type and animal strain were not sources of heterogeneity (*p* = 0.60, 0.21) (Supplementary Material S2.23, S2.24). Dose and TGP’s manufacturer (Institute of Clinical Pharmacology, Anhui Medical University, Ningbo Lihua Pharmaceutical Co., Ltd. and Ningbo Yicuijian Biological Technology Co., Ltd.) were sources of heterogeneity (*p* = 0.04, 0.002) (Supplementary Material S2.25, S2.26). The funnel plot (Supplementary Material S2.27) and Egger’s test (*p* < 0.001) indicated publication bias.

#### 3.4.3 IL-6

1) IL-6 (Intervention time is 1–2 weeks).

**FIGURE 6:**
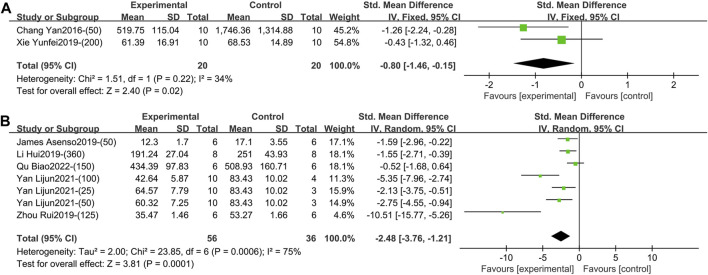
**(A)** Forest plot of IL-6 after 1–2 weeks of TGP intervention. **(B)** Forest plot of IL-6 after 3–4 weeks of TGP intervention.

Two studies containing 40 animals reported the effect of TGP intervention for 1–2 weeks (specifically 2 weeks) on serum IL-6 (Chang et al., 2016; Xie, 2019). Compared with the control group, TGP reduced IL-6 levels with low heterogeneity, so a fixed-effects model was used (SMD = −0.80, 95% CI [-1.46, −0.15], *p* = 0.02, *I* ^*2*^ = 34%) (Figure 6A; Supplementary Material S2.28).

2) IL-6 (Intervention time is 3–4 weeks).

Five studies involving 92 animals reported the effect of TGP on serum IL-6 after 3–4 weeks of intervention (Asenso et al., 2019; Li et al., 2019; Zhou et al., 2019; Yan et al., 2021; Qu et al., 2022). The combined results showed that TGP caused a significant reduction in IL-6 levels, but the heterogeneity was large (SMD = −2.48, 95% CI [-3.76, −1.21], *p* = 0.0001, *I* ^*2*^ = 75%) (Figure 6B; Supplementary Material S2.29). Sensitivity analysis showed that this result was robust (Supplementary Material S2.30). Subgroup analyses suggested that dose, model type, animal species, and strain were not sources of heterogeneity (*p* = 0.40, 0.95, 0.21, 0.21) (Supplementary Material S2.31–S2.34). However, TGP’s manufacturer (Institute of Clinical Pharmacology, Anhui Medical University, Ningbo Yicuijian Biological Technology Co., Ltd. and Ningbo Lihua Pharmaceutical Co., Ltd.) was a source of heterogeneity (*p* = 0.03) (Supplementary Material S2.35).

3) IL-6 (Intervention time is 8 weeks).

One study reported the effect of TGP intervention for 8 weeks on serum IL-6 (Shen et al., 2019). Only descriptive analysis was performed, TGP significantly reduced IL-6 levels compared with controls (SMD = −3.38, 95% CI [-5.12, −1.64], *p* = 0.0001) (Supplementary Material S2.36).

#### 3.4.4 IL-10

1) IL-10 (Intervention time is 1–2 weeks).

One study reported the effect of TGP intervention for 1–2 weeks (specifically 2 weeks) on serum IL-10 (Xie, 2019). Only descriptive analysis was performed, TGP tended to increase IL-10 levels compared with the control group, but the difference was not statistically significant (SMD = 0.64, 95% CI [-0.27, 1.54], *p* = 0.17) (Supplementary Material S2.37).

2) IL-10 (Intervention time is 3–4 weeks).

**FIGURE 7:**
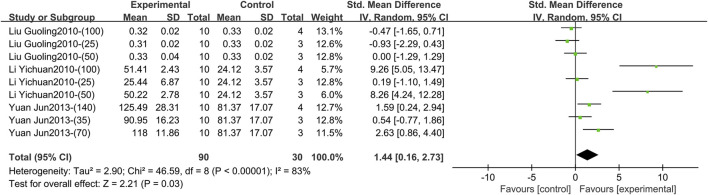
Forest plot of IL-10 after 3–4 weeks of TGP intervention.

Three studies containing 120 animals reported the effect of TGP on serum IL-10 after 3–4 weeks (specifically 3 weeks) of intervention (Li et al., 2010; Liu et al., 2010; Yuan, 2013). The combined results showed that TGP elevated IL-10 levels, but heterogeneity between studies was large (SMD = 1.44, 95% CI [0.16, 2.73], *p* = 0.03, *I* ^*2*^ = 83%) (Figure 7; Supplementary Material S2.38). Sensitivity analysis indicated that the results were robust (Supplementary Material S2.39). Dose, animal species, and strain were not sources of heterogeneity (*p* = 0.39, 0.90, 0.90) (Supplementary Material S2.40–S2.42).

2) IL-10 (Intervention time is 8 weeks)

One study reported the effect of TGP on serum IL-10 after 8 weeks of intervention (Shen et al., 2019). Only descriptive analysis was performed, TGP could increase IL-10 levels compared with the control group, and the difference was statistically significant (SMD = 1.86, 95% CI [0.58, 3.13], *p* = 0.004) (Supplementary Material S2.43).

#### 3.4.5 IL-1

1) IL-1 (Intervention time is 3–4 weeks)

**FIGURE 8:**
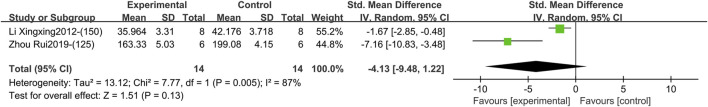
Forest plot of IL-1 after 3–4 weeks of TGP intervention.

Two studies containing 28 animals reported the effect of TGP intervention for 3–4 weeks (specifically 4 weeks) on serum IL-1 (Li, 2012; Zhou et al., 2019). The combined results showed that TGP failed to reduce IL-1 levels with a high degree of heterogeneity (SMD = −4.13, 95% CI [-9.48, 1.22], *p* = 0.13, *I* ^*2*^ = 87%) (Figure 8; Supplementary Material S2.44). The animal strains, model types, and manufacturers of TGP were the same in both studies, so the heterogeneity may be caused by different intervention times and doses.

#### 3.4.6 IL-2

1) IL-2 (Intervention time is 3–4 weeks)

**FIGURE 9:**
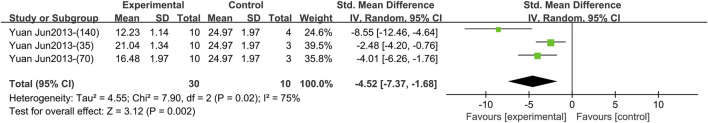
Forest plot of IL-2 after 3–4 weeks of TGP intervention.

One study containing three comparison groups reported the effect of TGP intervention for 3–4 weeks (specifically 3 weeks) on serum IL-2 (Yuan, 2013). The combined results indicated that IL-2 levels were significantly reduced, but the heterogeneity was large (SMD = −4.52, 95% CI [-7.37, −1.68], *p* = 0.002, *I* ^*2*^ = 75%) (Figure 9; Supplementary Material S2.45). Sensitivity analysis indicated that the result was robust (Supplementary Material S2.46). Subgroup analysis based on dose showed it was a source of heterogeneity (*p* = 0.01) (Supplementary Material S2.47).

#### 3.4.7 IL-4

1) IL-4 (Intervention time is 3–4 weeks).

**FIGURE 10:**
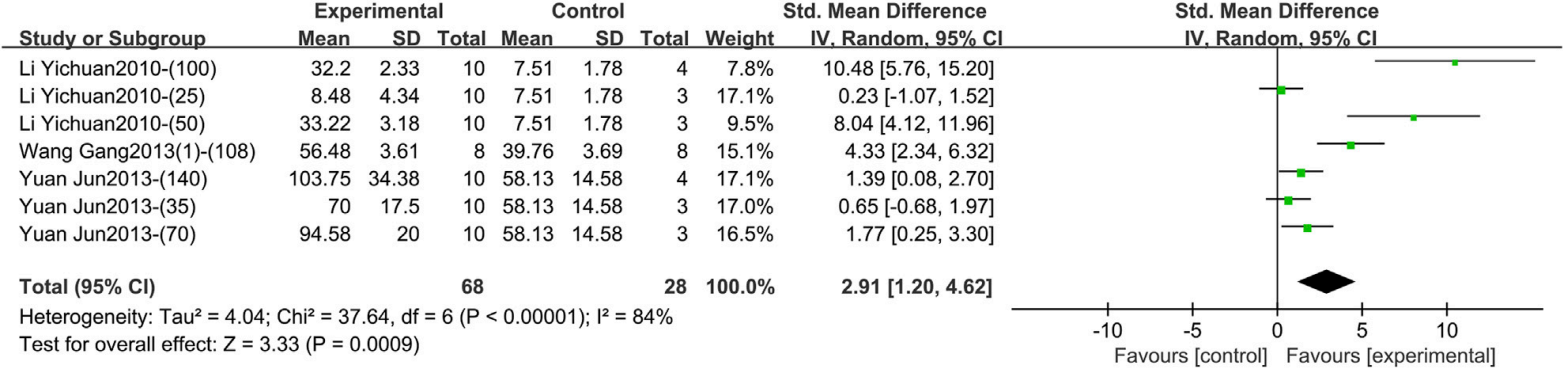
Forest plot of IL-4 after 3–4 weeks of TGP intervention.

Three studies including 96 animals reported the effect of TGP intervention for 3–4 weeks (specifically 3 weeks) on serum IL-4 (Li et al., 2010; Wang et al., 2013a; Yuan, 2013). The pooled results indicated that IL-4 levels were markedly elevated after TGP was used, but with great heterogeneity (SMD = 2.91, 95% CI [1.20, 4.62], *p* = 0.0009, *I* ^*2*^ = 84%) (Figure 10; Supplementary Material S2.48). The robustness of the result is proved by sensitivity analysis (Supplementary Material S2.49). Subgroup analyses found that dose, animal species and strain, and TGP’s manufacturer were not sources of heterogeneity (*p* = 0.18, 0.06, 0.06, 0.21) (Supplementary Material S2.50–S2.53).

#### 3.4.8 IL-17

1) IL-17 (Intervention time is 1–2 weeks).

One study reported the effect of TGP on serum IL-17 after 1–2 weeks (specifically 2 weeks) of intervention (Chang et al., 2016). Only descriptive analysis was performed, TGP tended to reduce IL-17 levels compared with model controls, but the difference was not statistically significant (SMD = −0.36, 95% CI [-1.25, 0.52], *p* = 0.42) (Supplementary Material S2.54).

2) IL-17 (Intervention time is 3–4 weeks).

**FIGURE 11:**
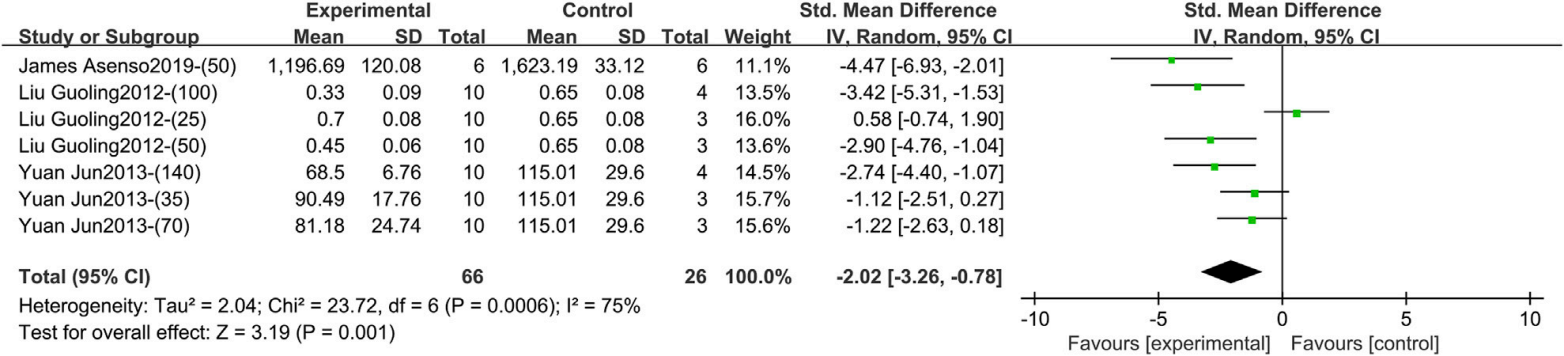
Forest plot of IL-17 after 3–4 weeks of TGP intervention.

Three studies containing 92 animals reported the effect of TGP intervention for 3–4 weeks (specifically 3 weeks) on serum IL-17 (Liu et al., 2012; Yuan, 2013; Asenso et al., 2019). The pooled results showed that TGP reduced IL-17 levels but with large heterogeneity (SMD = −2.02, 95% CI [-3.26, −0.78], *p* = 0.001, *I* ^*2*^ = 75%) (Figure 11; Supplementary Material S2.55). Sensitivity analysis indicated that the result was robust (Supplementary Material S2.56). Dose, model type, animal species, and strain were not sources of heterogeneity (*p* = 0.15, 0.05, 0.53, 0.53) (Supplementary Material S2.57–S2.60).

#### 3.4.9 IL-17α

1) IL-17α (Intervention time is 3–4 weeks).

One study reported the effect of TGP intervention for 3–4 weeks (specifically 4 weeks) on serum IL-17α (Zhou et al., 2018). Only descriptive analysis was performed, IL-17α levels were significantly lower in the experimental group than in the model control group (SMD = −2.27, 95% CI [-3.44, −1.09], *p* = 0.0002) (Supplementary Material S2.61).

2) IL-17α (Intervention time is 8 weeks).

One study reported the effect of TGP intervention for 8 weeks on serum IL-17α (Shen et al., 2019). Only descriptive analysis was performed, TGP markedly decreased IL- 17α levels *versus* controls (SMD = −4.47, 95% CI [-6.60, −2.35], *p* < 0.0001) (Supplementary Material S2.62).

#### 3.4.10 IL-21

1) IL-21 (Intervention time is 3–4 weeks)

One study reported the effect of TGP on serum IL-21 after 3–4 weeks (specifically 3 weeks) of intervention (Li et al., 2019). Only descriptive analysis was performed, the level of IL-21 was decreased after the use of TGP compared to controls (SMD = −3.26, 95% CI [-4.89, −1.63], *p* < 0.0001) (Supplementary Material S2.63).

#### 3.4.11 VEGF

1) VEGF (Intervention time is 3–4 weeks)

**FIGURE 12:**
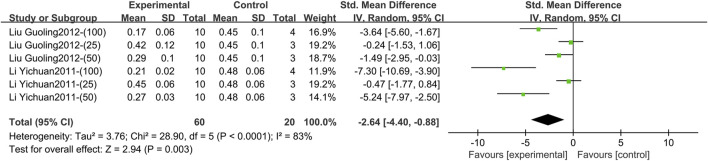
Forest plot of VEGF after 3–4 weeks of TGP intervention.

Two studies involving six comparison groups reported the effect of TGP intervention for 3–4 weeks on serum VEGF (Li et al., 2011; Liu et al., 2012). The pooled results showed that TGP resulted in a reduction in VEGF levels but with large heterogeneity (SMD = −2.64, 95% CI [-4.40, −0.88], *p* = 0.003, *I* ^*2*^ = 83%) (Figure 12; Supplementary Material S2.64). Sensitivity analysis indicated that this result was robust (Supplementary Material S2.65). Dose subgroup analysis indicated that it was not a source of heterogeneity (*p* = 0.06) (Supplementary Material S2.66).

#### 3.4.12 IFN-γ

1) IFN-γ (Intervention time is 1–2 weeks)

**FIGURE 13:**
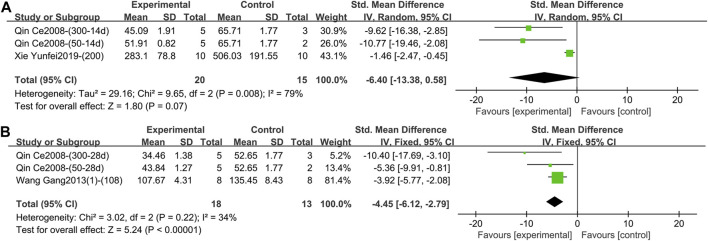
**(A)** Forest plot of IFN-γ after 1–2 weeks of TGP intervention. **(B)** Forest plot of IFN-γ after 3–4 weeks of TGP intervention.

Two studies containing 35 animals reported the effect of TGP intervention for 1–2 weeks on serum IFN-γ (Qin, 2008; Xie, 2019). The pooled results showed that the difference in IFN-γ between the two groups was not statistically significant and the heterogeneity was large (SMD = −6.40, 95% CI [-13.38, 0.58], *p* = 0.07, *I* ^*2*^ = 79%) (Figure 13A; Supplementary Material S2.67). Sensitivity analysis showed that the result was robust (Supplementary Material S2.68). Subgroup analyses suggested that dose was not a source of heterogeneity (*p* = 0.32) (Supplementary Material S2.69), but animal strain (SD rat, Wistar rat) and TGP’s manufacturer [Ningbo Lihua Pharmaceutical Co., Ltd. and Anhui Bozhou Chinese herbal medicine market (self-made purification)] were sources of heterogeneity (*p* = 0.002, 0.002) (Supplementary Material S2.70, S2.71).

2) IFN-γ (Intervention time is 3–4 weeks).

Two studies containing three comparison groups reported the effect of TGP intervention for 3–4 weeks on serum IFN-γ (Qin, 2008; Wang et al., 2013a). The pooled results showed that TGP dramatically decreased IFN-γ levels with low heterogeneity (SMD = −4.45, 95% CI [-6.12, −2.79], *p* < 0.00001, *I* ^*2*^ = 34%), so a fixed-effects model was used (Figure 13B; Supplementary Material S2.72).

#### 3.4.13 PGE2

1) PGE2 (Intervention time is 1–2 weeks).

One study reported the effect of TGP on serum PGE2 after 1–2 weeks (specifically 2 weeks) of intervention (Jia, 2015). Only descriptive analysis was performed, TGP could reduce PGE2 levels compared with the control group, and the variance was statistically significant (SMD = −8.03, 95% CI [-10.94, −5.12], *p* < 0.00001) (Supplementary Material S2.73).

2) PGE2 (Intervention time is 3–4 weeks)

One study reported the effect of TGP intervention for 3–4 weeks (specifically 4 weeks) on serum PGE2 (Zhou et al., 2019). Only descriptive analysis was performed, TGP dramatically lowered PGE2 levels compared with controls (SMD = −12.77, 95% CI [-19.11, −6.44], *p* < 0.0001) (Supplementary Material S2.74).

#### 3.4.14 TGF-β1

1) TGF-β1 (Intervention time is 1–2 weeks).

One study reported the effect of TGP on serum TGF-β1 after 1–2 weeks (specifically 2 weeks) of intervention (Chang et al., 2016). Only descriptive analysis was performed, TGP increased TGF-β1 levels compared with no TGP, and the difference was statistically significant (SMD = 1.29, 95% CI [0.31, 2.28], *p* = 0.01) (Supplementary Material S2.75).

2) TGF-β1 (Intervention time is 3–4 weeks).

**FIGURE 14:**
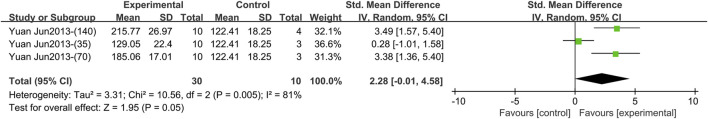
Forest plot of TGF-β1 after 3–4 weeks of TGP intervention.

One study involving three comparison groups reported the effect of TGP on serum TGF-β1 after 3–4 weeks (specifically 4 weeks) of intervention (Yuan, 2013). The pooled results suggested that TGP tended to increase TGF-β1 levels, but the difference was not statistically significant and heterogeneity was high (SMD = 2.28, 95% CI [-0.01, 4.58], *p* = 0.05, *I* ^*2*^ = 81%) (Figure 14; Supplementary Material S2.76). The robustness of the result was demonstrated by sensitivity analysis (Supplementary Material S2.77). Subgroup analysis showed that dose was not a source of heterogeneity (*p* = 0.34) (Supplementary Material S2.78).

### 3.5 Potential mechanisms of TGP on RA in the included studies

This study summarized potential mechanisms of TGP on RA in the included studies, which were mainly divided into four aspects: regulating inflammatory cytokines, inhibiting synoviocyte proliferation and promoting synoviocyte apoptosis, inhibiting bone destruction, and regulating immune function. The specific mechanisms are shown in Table 2.

**TABLE 2:** Table of potential mechanisms of TGP on RA in the included studies.

Aspects	Potential mechanisms	References
Regulating inflammatory cytokines	Reduce the levels of serum TNF-α, IL-1β, VEGF, PGE2, IL-2, IL-6, IL-17, IL-17α, IL-21, IFN-γ; Increase the levels of serum IL-4, IL-10, TGF-β1; Inhibit the production of inflammatory mediators and factors in the local area, and induce the balance of inflammatory cytokine network; Inhibit the interaction network between NF-κB/p65 and cytokines, inhibit vascular hyperplasia and inflammatory cell infiltration; Reduce the levels of IL-1β and IFN-γ in synovium, reduce joint swelling and inhibit bone destruction; Inhibit the expression of NF-κB p65 subunit, TNF-a, COX-2	Asenso et al. (2019); Chang et al. (2016); Jia, (2015); Li et al. (2019); Li, (2012); Li et al. (2011); Li et al. (2010); Liu et al. (2010); Liu et al. (2012); Peng et al. (2012); Qin, (2008); Shen et al. (2019); Wang et al. (2013a); Wang et al. (2013b); Yan et al. (2021); Yao, (2007); Yuan, (2013); Zhou et al. (2019); Zhou et al. (2018); Zhou et al. (2012); Zhou et al. (2009)
Inhibiting synoviocyte proliferation and promoting synoviocyte apoptosis	Reduce synoviocyte edema and inhibit synovial hyperplasia; Promote the expression of Cleaved caspase-3, Bax and inhibit the expression of Bcl-2 by inhibiting the TLR4/NF-κB signaling pathway, reduce inflammatory response and promote apoptosis of joint synovium cells	Qin, (2008); Yan et al. (2021)
Inhibiting bone destruction	Inhibit osteoclast differentiation mediated by NF-κB and STAT3 signaling pathways, and enhance osteoblast function mediated by Wnt/β-catenin signaling pathways	Shen et al. (2019)
Regulating immune function	Reduce the effect of inflammatory factors on immune dysfunction; Reduce the expression level of CD4 ^+^ T cells, correct the disordered immune function and restore the immune balance; Regulate the functions of T and B lymphocytes and peritoneal macrophage; Inhibit the differentiation of splenic Tfh cells and the formation of germinal centers through STAT3 signaling pathway, thus suppressing autoimmune reactions	Yao, (2007); Li, (2012); Yuan, (2013); Li et al. (2019)

## 4 Discussion

### 4.1 Summary of results

By combining 24 studies, this article found that compared to the model control group, TGP decreased the levels of TNF-α, IL-1β, IL-6, and PGE2 and increased the levels of TGF-β1 after 1–2 weeks of intervention. After 3–4 weeks of intervention, the levels of TNF-α, IL-1β, IL-6, and so on were decreased and the levels of IL-10 and IL-4 were increased. After 8 weeks of intervention, the levels of TNF-α, IL-6, and IL-17α were decreased and the level of IL-10 was increased. There was no significant difference in the effects of TGP on the levels of IL-10, IL-17, and IFN-γ after 1–2 weeks of intervention and IL-1 and TGF-β1 after 3–4 weeks of intervention. The specific results are summarized in Table 3.

**TABLE 3:** Summary of results.

Outcome	Intervention time	Result
TNF-α	1–2 weeks	SMD = −2.29, 95% CI [-3.55, −1.03], *p* = 0.0004, *I* ^*2*^ = 85%
	3–4 weeks	SMD = −2.14, 95% CI [-2.82, −1.47], *p* < 0.00001, *I* ^*2*^ = 73%
	8 weeks	SMD = −4.43, 95% CI [-6.53, −2.32], *p* < 0.0001
IL-1β	1–2 weeks	SMD = −2.94, 95% CI [-4.76, −1.11], *p* = 0.002, *I* ^*2*^ = 83%
	3–4 weeks	SMD = −2.84, 95% CI [-3.99, −1.70], *p* < 0.00001, *I* ^*2*^ = 81%
IL-6	1–2 weeks	SMD = −0.80, 95% CI [-1.46, −0.15], *p* = 0.02, *I* ^*2*^ = 34%
	3–4 weeks	SMD = −2.48, 95% CI [-3.76, −1.21], *p* = 0.0001, *I* ^*2*^ = 75%
	8 weeks	SMD = −3.38, 95% CI [-5.12, −1.64], *p* = 0.0001
IL-10	1–2 weeks	SMD = 0.64, 95% CI [-0.27, 1.54], *p* = 0.17
	3–4 weeks	SMD = 1.44, 95% CI [0.16, 2.73], *p* = 0.03, *I* ^*2*^ = 83%
	8 weeks	SMD = 1.86, 95% CI [0.58, 3.13], *p* = 0.004
IL-1	3–4 weeks	SMD = −4.13, 95% CI [-9.48, 1.22], *p* = 0.13, *I* ^*2*^ = 87%
IL-2	3–4 weeks	SMD = −4.52, 95% CI [-7.37, −1.68], *p* = 0.002, *I* ^*2*^ = 75%
IL-4	3–4 weeks	SMD = 2.91, 95% CI [1.20, 4.62], *p* = 0.0009, *I* ^*2*^ = 84%
IL-17	1–2 weeks	SMD = −0.36, 95% CI [-1.25, 0.52], *p* = 0.42
	3–4 weeks	SMD = −2.02, 95% CI [-3.26, −0.78], *p* = 0.001, *I* ^*2*^ = 75%
IL-17α	3–4 weeks	SMD = −2.27, 95% CI [-3.44, −1.09], *p* = 0.0002
	8 weeks	SMD = −4.47, 95% CI [-6.60, −2.35], *p* < 0.0001
IL-21	3–4 weeks	SMD = −3.26, 95% CI [-4.89, −1.63], *p* < 0.0001
VEGF	3–4 weeks	SMD = −2.64, 95% CI [-4.40, −0.88], *p* = 0.003, *I* ^*2*^ = 83%
IFN-γ	1–2 weeks	SMD = −6.40, 95% CI [-13.38, 0.58], *p* = 0.07, *I* ^*2*^ = 79%
	3–4 weeks	SMD = −4.45, 95% CI [-6.12, −2.79], *p* < 0.00001, *I* ^*2*^ = 34%
PGE2	1–2 weeks	SMD = −8.03, 95% CI [-10.94, −5.12], *p* < 0.00001
	3–4 weeks	SMD = −12.77, 95% CI [-19.11, −6.44], *p* < 0.0001
TGF-β1	1–2 weeks	SMD = 1.29, 95% CI [0.31, 2.28], *p* = 0.01
	3–4 weeks	SMD = 2.28, 95% CI [-0.01, 4.58], *p* = 0.05, *I* ^*2*^ = 81%

### 4.2 Heterogeneity interpretation

Due to the differences in animal species, strain, model type, TGP’s dose and manufacturer, intervention time, and other factors, clinical heterogeneity may be caused. Therefore, we reduced the possible clinical heterogeneity from two perspectives. First, before including the studies, we limited the dose to no more than twice the standard dose of the corresponding species. And the route of administration is limited to gavage. Second, before the results were combined, they were divided into 1–2 weeks, 2–4 weeks, and 8 weeks according to the different intervention times of the included studies.

After combining the results, this study found that the heterogeneity of most results was generally high. For this reason, the sensitivity analyses performed first proved that the results were all robust, and the subgroup analyses performed later found that the heterogeneity mainly originated from the animal strain, TGP’s manufacturer, and dose, while the animal species and model type were not the sources of heterogeneity. In terms of model types, neither AIA nor CIA can fully imitate human RA and can only reflect the nature of the disease from some aspects. Although there may be clinical heterogeneity between them, the results of this study showed that the model type was not the source of heterogeneity.

Although many studies were included in this study, most of them reported fewer outcomes, resulting in fewer comparison groups included in some outcomes. Moreover, the sources of heterogeneity are not consistent across outcomes, so sources of heterogeneity obtained through subgroup analyses need to be viewed with caution (Sun et al., 2010).

### 4.3 Implications for future research

This study has five implications for future research. First of all, there may be some methodological heterogeneity in the included studies. There were deficiencies or lack of relevant instructions in random sequence generation, baseline characteristics, allocation concealment, blinding of animal keepers and investigators, and blinding of outcome evaluators, so it is hoped that future researchers will be more rigorous in designing and executing their experimental protocols, and more standardized in writing their manuscripts.

Secondly, the outcomes vary greatly among the included studies, and core outcome sets need to be studied and popularized (Kirkham et al., 2017). And then, at present, the experimental animals used in the relevant studies are mainly in the lower age stages, and there is a lack of studies in the higher age stages. It is hoped that future researchers can consider that the main incidence population of RA is in the high age stage, and supplement the research of related age stages.

Next, the intervention time of TGP was obviously discontinuous, and there was a lack of studies on corresponding intervention time (5–7 weeks or even longer). It is not difficult to find that the low, medium, and high dosages in some studies were 25, 50, and 100 mg·kg-1·day-1, respectively (Li et al., 2010; Liu et al., 2010; Li et al., 2011; Liu et al., 2012; Yan et al., 2021), which were different from the pre-designed dose subgroup analysis. This may be due to the fact that researchers are not starting from a clinical reality. Therefore, the intervention time and dose of TGP need to be reported more comprehensively, the minimum intervention time and dose that begin to take effect should reach a consensus, and the maximum intervention time and dose that stop increasing effect remain to be investigated. More high-quality animal studies are expected to emerge as a reference for clinical research. Finally, funnel plots of TNF-α (Intervention time is 3–4 weeks) and IL-1β (Intervention time is 3–4 weeks) suggest publication bias, so negative results need to be encouraged for publication.

### 4.4 Advantages and disadvantages

This study is the first systematic review and meta-analysis of the effects of TGP on serum inflammatory cytokines in animal models of RA, with a complete set of outcomes included. There is a comprehensive study in this aspect. However, some studies have also reported local-level outcomes such as inflammatory factors and protein molecules in foot and claw synovial tissues, articular cartilage, and joint fluids. For example, TGP may exert anti-inflammatory effects by regulating the balance between Gαi and Gαs and decreasing the expression of β-arrestins (Wang et al., 2011; Jia et al., 2014). This aspect is not covered in this study, mainly due to the difference in measurement specimens and the lack of relevant studies. This perspective is expected to serve as an entry point for future systematic reviews and Meta-analyses to further evaluate the improvement of localized lesions in RA after drug administration, which will help to uncover and summarize the mechanism of TGP in the treatment of RA.

## 5 Conclusion

In summary, based on the existing studies, this study found that compared with the control group of the RA animal model, TGP can reduce the levels of serum pro-inflammatory cytokines such as TNF-α, IL-1β, and IL-6 and increase the levels of serum anti-inflammatory cytokines such as IL-10, exerting an anti-inflammatory effect by regulating and improving the levels of inflammatory cytokines and thus alleviating the disease. Given the low quality of the included studies and the lack of sufficient evidence, more high-quality studies are still needed to validate the results of this study.

## Data Availability

The original contributions presented in the study are included in the article/Supplementary Material, further inquiries can be directed to the corresponding author.

## Abbreviations

AIA, adjuvant-induced arthritis; CI, confidence interval; CIA, collagen-induced arthritis; CNKI, China National Knowledge Infrastructure; IFN-γ, interferon-γ; i. g., gavage; IL-1, interleukin-1; IL-1β, interleukin-1β; IL-2, interleukin-2; IL-4, interleukin-4; IL-6, interleukin-6; IL-10, interleukin-10; IL-17, interleukin-17; IL-17α, interleukin-17α; IL-21, interleukin-21; PGE2, prostaglandin E2; RA, rheumatoid arthritis; SD, Sprague Dawley; SMD, standardized mean difference; TGF-β1, transforming growth factor-β1; TGP, total glucosides of paeony; TNF-α, tumor necrosis factor-α; VEGF, vascular endothelial growth factor; WMD, weighted mean difference.
