# Supplementary material for: Effects of total glucosides of paeony on serum inflammatory cytokines in animal models of rheumatoid arthritis: a systematic review and meta-analysis
Source: Front Pharmacol. 2024 Mar 25;15:1349259. doi: 10.3389/fphar.2024.1349259 (PMC10999591; doi:10.3389/fphar.2024.1349259)

**Table of Contents**

[1. Search strategies in databases 4](#_Toc152536683)

[1.1 CNKI (545) 4](#_Toc152536684)

[1.2 Wanfang (462) 4](#_Toc152536685)

[1.3 VIP (295) 5](#_Toc152536686)

[1.4 PubMed (86) 6](#_Toc152536687)

[1.5 EMBASE (77) 6](#_Toc152536688)

[1.6 Cochrane Library (13) 7](#_Toc152536689)

[2. Supporting information for data analysis 9](#_Toc152536690)

[2.1 Forest plot of TNF-α after 1-2 weeks of TGP intervention 9](#_Toc152536691)

[2.2 Sensitivity analysis of TNF-α after 1-2 weeks of TGP intervention 9](#_Toc152536692)

[2.3 Subgroup analysis of TNF-α after 1-2 weeks of TGP intervention according to doses 10](#_Toc152536693)

[2.4 Subgroup analysis of TNF-α after 1-2 weeks of TGP intervention according to animal model types 10](#_Toc152536694)

[2.5 Subgroup analysis of TNF-α after 1-2 weeks of TGP intervention according to TGP 's manufacturers 11](#_Toc152536695)

[2.6 Subgroup analysis of TNF-α after 1-2 weeks of TGP intervention according to animal strains 11](#_Toc152536696)

[2.7 Forest plot of TNF-α after 3-4 weeks of TGP intervention 12](#_Toc152536697)

[2.8 Sensitivity analysis of TNF-α after 3-4 weeks of TGP intervention 12](#_Toc152536698)

[2.9 Subgroup analysis of TNF-α after 3-4 weeks of TGP intervention according to doses 13](#_Toc152536699)

[2.10 Subgroup analysis of TNF-α after 3-4 weeks of TGP intervention according to animal model types 13](#_Toc152536700)

[2.11 Subgroup analysis of TNF-α after 3-4 weeks of TGP intervention according to animal species 14](#_Toc152536701)

[2.12 Subgroup analysis of TNF-α after 3-4 weeks of TGP intervention according to animal strains 14](#_Toc152536702)

[2.13 Funnel plot of TNF-α after 3-4 weeks of TGP intervention 15](#_Toc152536703)

[2.14 Forest plot of TNF-α after 8 weeks of TGP intervention 15](#_Toc152536704)

[2.15 Forest plot of IL-1β after 1-2 weeks of TGP intervention 15](#_Toc152536705)

[2.16 Sensitivity analysis of IL-1β after 1-2 weeks of TGP intervention 16](#_Toc152536706)

[2.17 Subgroup analysis of IL-1β after 1-2 weeks of TGP intervention according to doses 16](#_Toc152536707)

[2.18 Subgroup analysis of IL-1β after 1-2 weeks of TGP intervention according to animal model types 17](#_Toc152536708)

[2.19 Subgroup analysis of IL-1β after 1-2 weeks of TGP intervention according to TGP 's manufacturers 17](#_Toc152536709)

[2.20 Subgroup analysis of IL-1β after 1-2 weeks of TGP intervention according to animal strains 18](#_Toc152536710)

[2.21 Forest plot of IL-1β after 3-4 weeks of TGP intervention 18](#_Toc152536711)

[2.22 Sensitivity analysis of IL-1β after 3-4 weeks of TGP intervention 19](#_Toc152536712)

[2.23 Subgroup analysis of IL-1β after 3-4 weeks of TGP intervention according to animal model types 19](#_Toc152536713)

[2.24 Subgroup analysis of IL-1β after 3-4 weeks of TGP intervention according to animal strains 20](#_Toc152536714)

[2.25 Subgroup analysis of IL-1β after 3-4 weeks of TGP intervention according to doses 20](#_Toc152536715)

[2.26 Subgroup analysis of IL-1β after 3-4 weeks of TGP intervention according to TGP 's manufacturers 21](#_Toc152536716)

[2.27 Funnel plot of IL-1β after 3-4 weeks of TGP intervention 22](#_Toc152536717)

[2.28 Forest plot of IL-6 after 1-2 weeks of TGP intervention 22](#_Toc152536718)

[2.29 Forest plot of IL-6 after 3-4 weeks of TGP intervention 22](#_Toc152536719)

[2.30 Sensitivity analysis of IL-6 after 3-4 weeks of TGP intervention 23](#_Toc152536720)

[2.31 Subgroup analysis of IL-6 after 3-4 weeks of TGP intervention according to doses 23](#_Toc152536721)

[2.32 Subgroup analysis of IL-6 after 3-4 weeks of TGP intervention according to animal model types 24](#_Toc152536722)

[2.33 Subgroup analysis of IL-6 after 3-4 weeks of TGP intervention according to animal species 24](#_Toc152536723)

[2.34 Subgroup analysis of IL-6 after 3-4 weeks of TGP intervention according to animal strains 25](#_Toc152536724)

[2.35 Subgroup analysis of IL-6 after 3-4 weeks of TGP intervention according to TGP 's manufacturers 25](#_Toc152536725)

[2.36 Forest plot of IL-6 after 8 weeks of TGP intervention 25](#_Toc152536726)

[2.37 Forest plot of IL-10 after 1-2 weeks of TGP intervention 26](#_Toc152536727)

[2.38 Forest plot of IL-10 after 3-4 weeks of TGP intervention 26](#_Toc152536728)

[2.39 Sensitivity analysis of IL-10 after 3-4 weeks of TGP intervention 26](#_Toc152536729)

[2.40 Subgroup analysis of IL-10 after 3-4 weeks of TGP intervention according to doses 27](#_Toc152536730)

[2.41 Subgroup analysis of IL-10 after 3-4 weeks of TGP intervention according to animal species 27](#_Toc152536731)

[2.42 Subgroup analysis of IL-10 after 3-4 weeks of TGP intervention according to animal strains 28](#_Toc152536732)

[2.43 Forest plot of IL-10 after 8 weeks of TGP intervention 28](#_Toc152536733)

[2.44 Forest plot of IL-1 after 3-4 weeks of TGP intervention 28](#_Toc152536734)

[2.45 Forest plot of IL-2 after 3-4 weeks of TGP intervention 28](#_Toc152536735)

[2.46 Sensitivity analysis of IL-2 after 3-4 weeks of TGP intervention 29](#_Toc152536736)

[2.47 Subgroup analysis of IL-2 after 3-4 weeks of TGP intervention according to doses 29](#_Toc152536737)

[2.48 Forest plot of IL-4 after 3-4 weeks of TGP intervention 30](#_Toc152536738)

[2.49 Sensitivity analysis of IL-4 after 3-4 weeks of TGP intervention 30](#_Toc152536739)

[2.50 Subgroup analysis of IL-4 after 3-4 weeks of TGP intervention according to doses 31](#_Toc152536740)

[2.51 Subgroup analysis of IL-4 after 3-4 weeks of TGP intervention according to animal species 31](#_Toc152536741)

[2.52 Subgroup analysis of IL-4 after 3-4 weeks of TGP intervention according to animal strains 32](#_Toc152536742)

[2.53 Subgroup analysis of IL-4 after 3-4 weeks of TGP intervention according to TGP 's manufacturers 32](#_Toc152536743)

[2.54 Forest plot of IL-17 after 1-2 weeks of TGP intervention 32](#_Toc152536744)

[2.55 Forest plot of IL-17 after 3-4 weeks of TGP intervention 33](#_Toc152536745)

[2.56 Sensitivity analysis of IL-17 after 3-4 weeks of TGP intervention 33](#_Toc152536746)

[2.57 Subgroup analysis of IL-17 after 3-4 weeks of TGP intervention according to doses 34](#_Toc152536747)

[2.58 Subgroup analysis of IL-17 after 3-4 weeks of TGP intervention according to animal model types 34](#_Toc152536748)

[2.59 Subgroup analysis of IL-17 after 3-4 weeks of TGP intervention according to animal species 35](#_Toc152536749)

[2.60 Subgroup analysis of IL-17 after 3-4 weeks of TGP intervention according to animal strains 35](#_Toc152536750)

[2.61 Forest plot of IL-17α after 3-4 weeks of TGP intervention 35](#_Toc152536751)

[2.62 Forest plot of IL-17α after 8 weeks of TGP intervention 36](#_Toc152536752)

[2.63 Forest plot of IL-21 after 3-4 weeks of TGP intervention 36](#_Toc152536753)

[2.64 Forest plot of VEGF after 3-4 weeks of TGP intervention 36](#_Toc152536754)

[2.65 Sensitivity analysis of VEGF after 3-4 weeks of TGP intervention 37](#_Toc152536755)

[2.66 Subgroup analysis of VEGF after 3-4 weeks of TGP intervention according to doses 37](#_Toc152536756)

[2.67 Forest plot of IFN-γ after 1-2 weeks of TGP intervention 38](#_Toc152536757)

[2.68 Sensitivity analysis of IFN-γ after 1-2 weeks of TGP intervention 38](#_Toc152536758)

[2.69 Subgroup analysis of IFN-γ after 1-2 weeks of TGP intervention according to doses 39](#_Toc152536759)

[2.70 Subgroup analysis of IFN-γ after 1-2 weeks of TGP intervention according to animal strains 39](#_Toc152536760)

[2.71 Subgroup analysis of IFN-γ after 1-2 weeks of TGP intervention according to TGP 's manufacturers 40](#_Toc152536761)

[2.72 Forest plot of IFN-γ after 3-4 weeks of TGP intervention 40](#_Toc152536762)

[2.73 Forest plot of PGE2 after 1-2 weeks of TGP intervention 40](#_Toc152536763)

[2.74 Forest plot of PGE2 after 3-4 weeks of TGP intervention 40](#_Toc152536764)

[2.75 Forest plot of TGF-β1 after 1-2 weeks of TGP intervention 41](#_Toc152536765)

[2.76 Forest plot of TGF-β1 after 3-4 weeks of TGP intervention 41](#_Toc152536766)

[2.77 Sensitivity analysis of TGF-β1 after 3-4 weeks of TGP intervention 41](#_Toc152536767)

[2.78 Subgroup analysis of TGF-β1 after 3-4 weeks of TGP intervention according to doses 42](#_Toc152536768)

# Search strategies in databases

## 1.1 CNKI (545)

The database search in CNKI was carried out on August 14, 2023, and a total of 545 studies were found.

Search formulation:

((SU %= '白芍总苷胶囊' OR SU %= '白芍总苷' OR SU %= '帕夫林胶囊' OR SU %= '帕夫林' OR SU %= '芍药总苷') OR (TKA = '白芍总苷胶囊' OR TKA = '白芍总苷' OR TKA = '帕夫林胶囊' OR TKA = '帕夫林' OR TKA = '芍药总苷')) AND ((SU %= '类风湿性关节炎' OR SU %= '类风湿关节炎' OR SU %= '类风关' OR SU %= '痹症' OR SU %= '尫痹' OR SU %= '实验性关节炎' OR SU %= '佐剂性关节炎' OR SU %= '佐剂诱导' OR SU %= '胶原性关节炎' OR SU %= '胶原诱导') OR (TKA = '类风湿性关节炎' OR TKA = '类风湿关节炎' OR TKA = '类风关' OR TKA = '痹症' OR TKA = '尫痹' OR TKA = '实验性关节炎' OR TKA = '佐剂性关节炎' OR TKA = '佐剂诱导' OR TKA = '胶原性关节炎' OR TKA = '胶原诱导'))


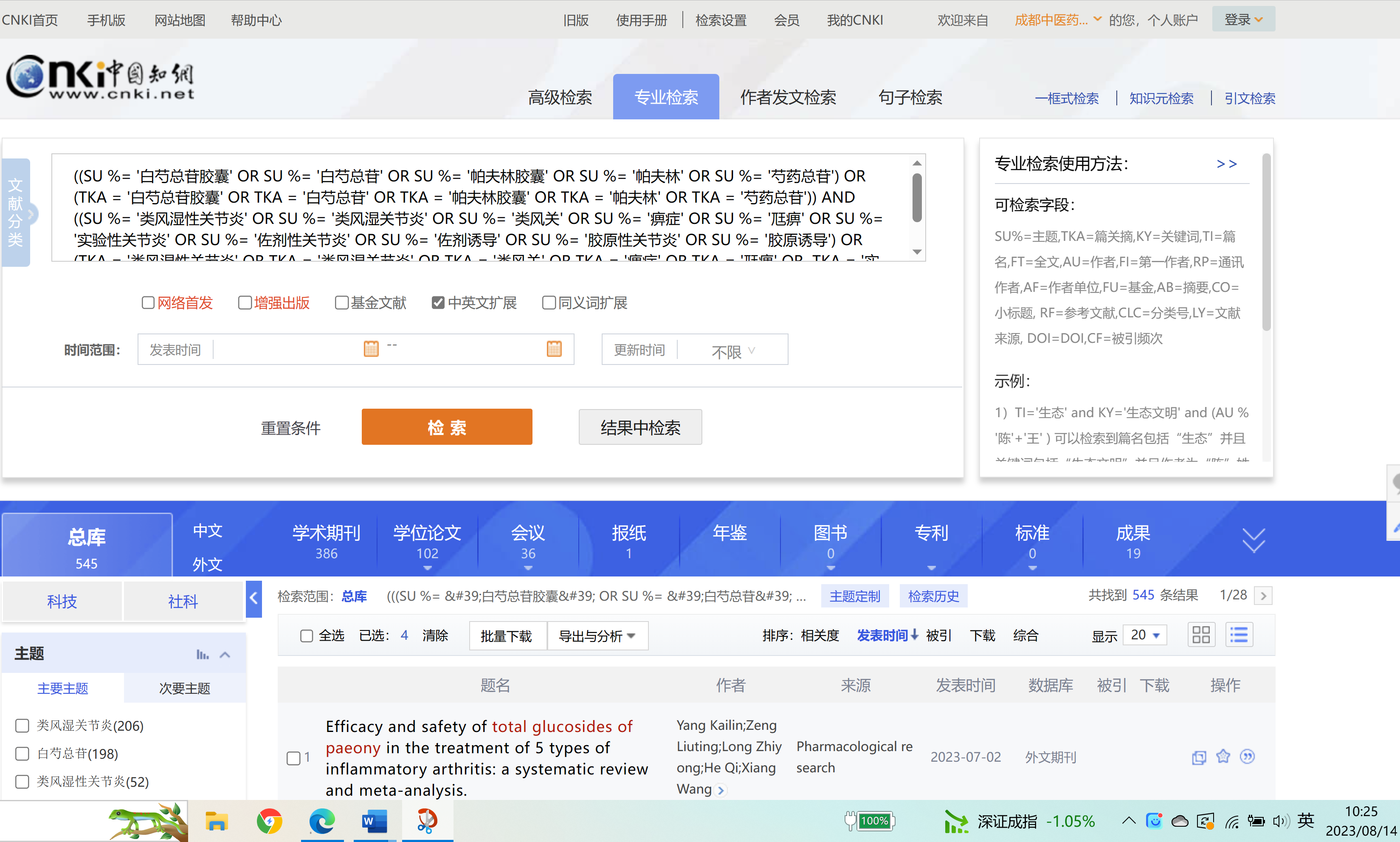


## 1.2 Wanfang (462)

The database search in Wanfang was carried out on August 14, 2023, and a total of 462 studies were found.


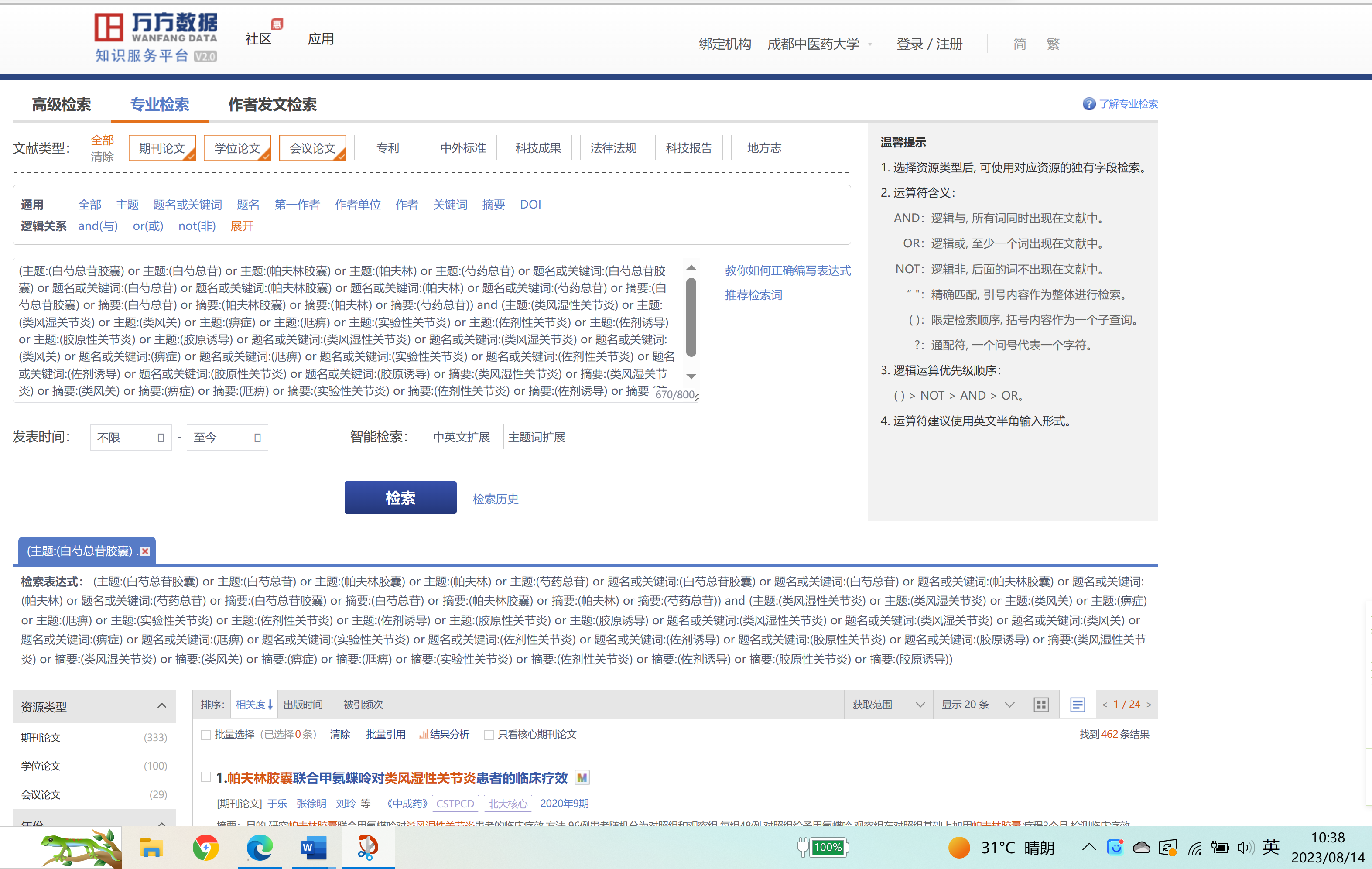


## 1.3 VIP (295)

The database search in VIP was carried out on August 14, 2023, and a total of 301 studies were found, but only 295 literatures were obtained.


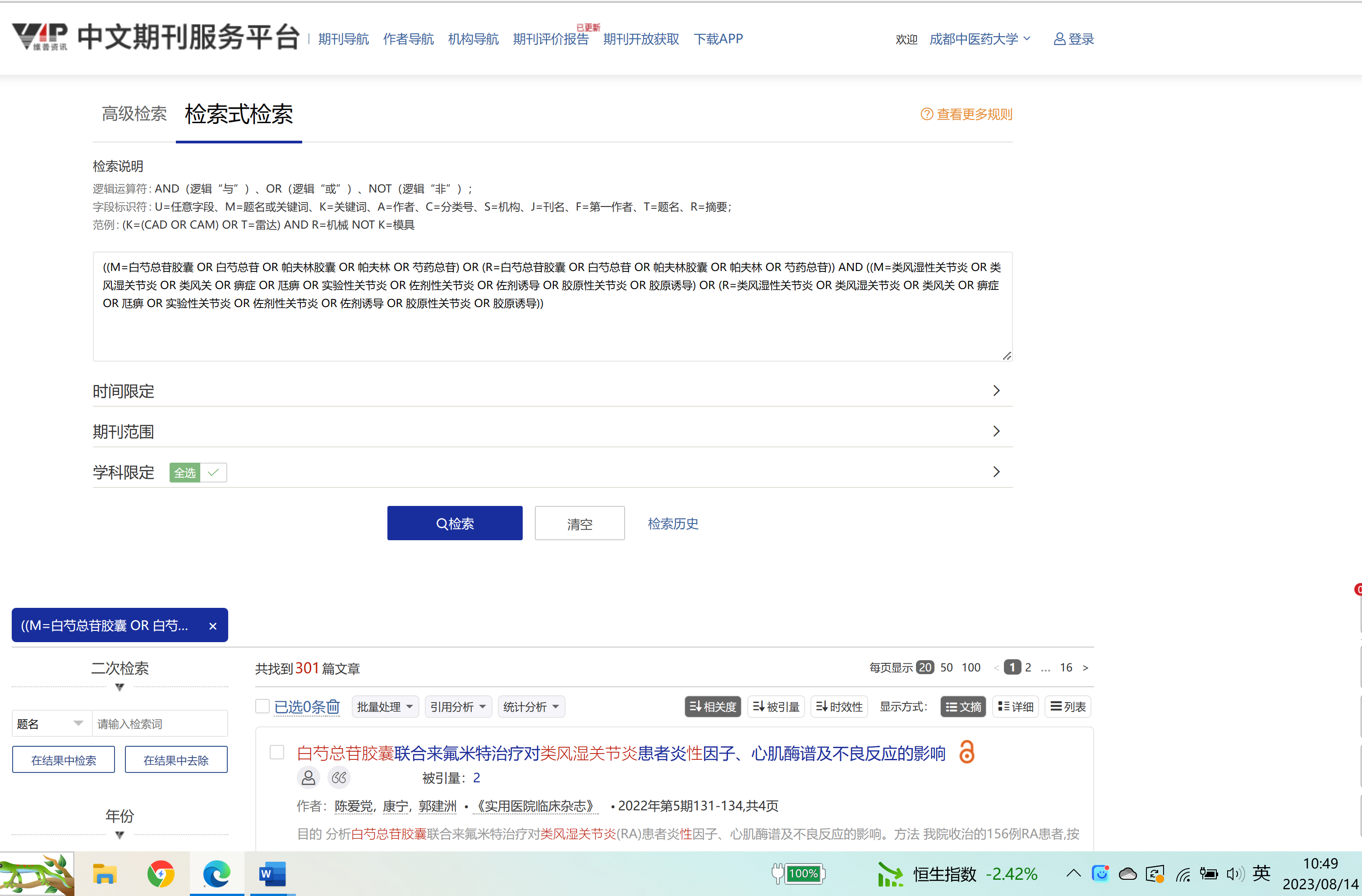


## 1.4 PubMed (86)

The database search in PubMed was carried out on August 14, 2023, and a total of 86 studies were found.


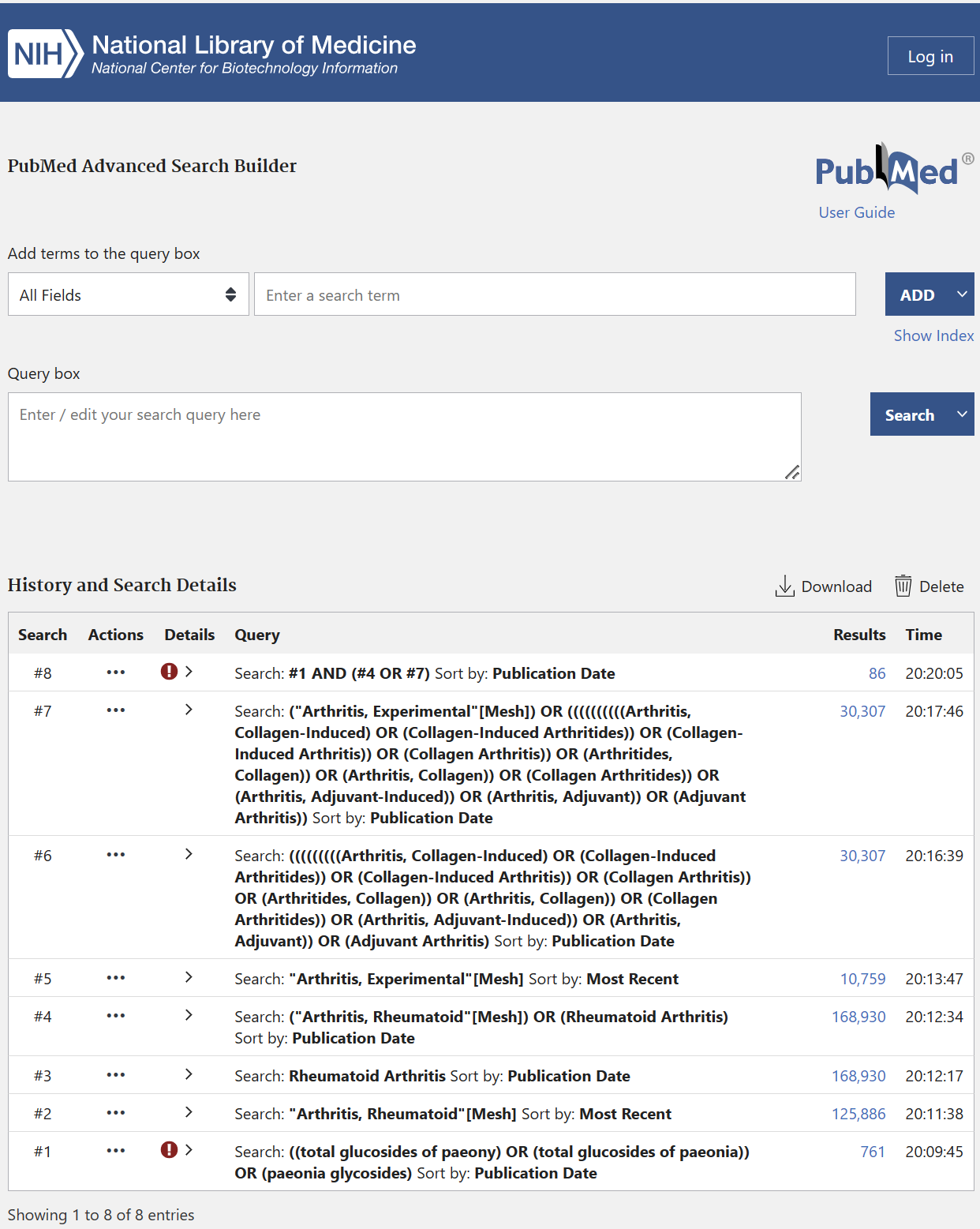


## 1.5 EMBASE (77)

The database search in EMBASE was carried out on August 14, 2023, and a total of 77 studies were found.


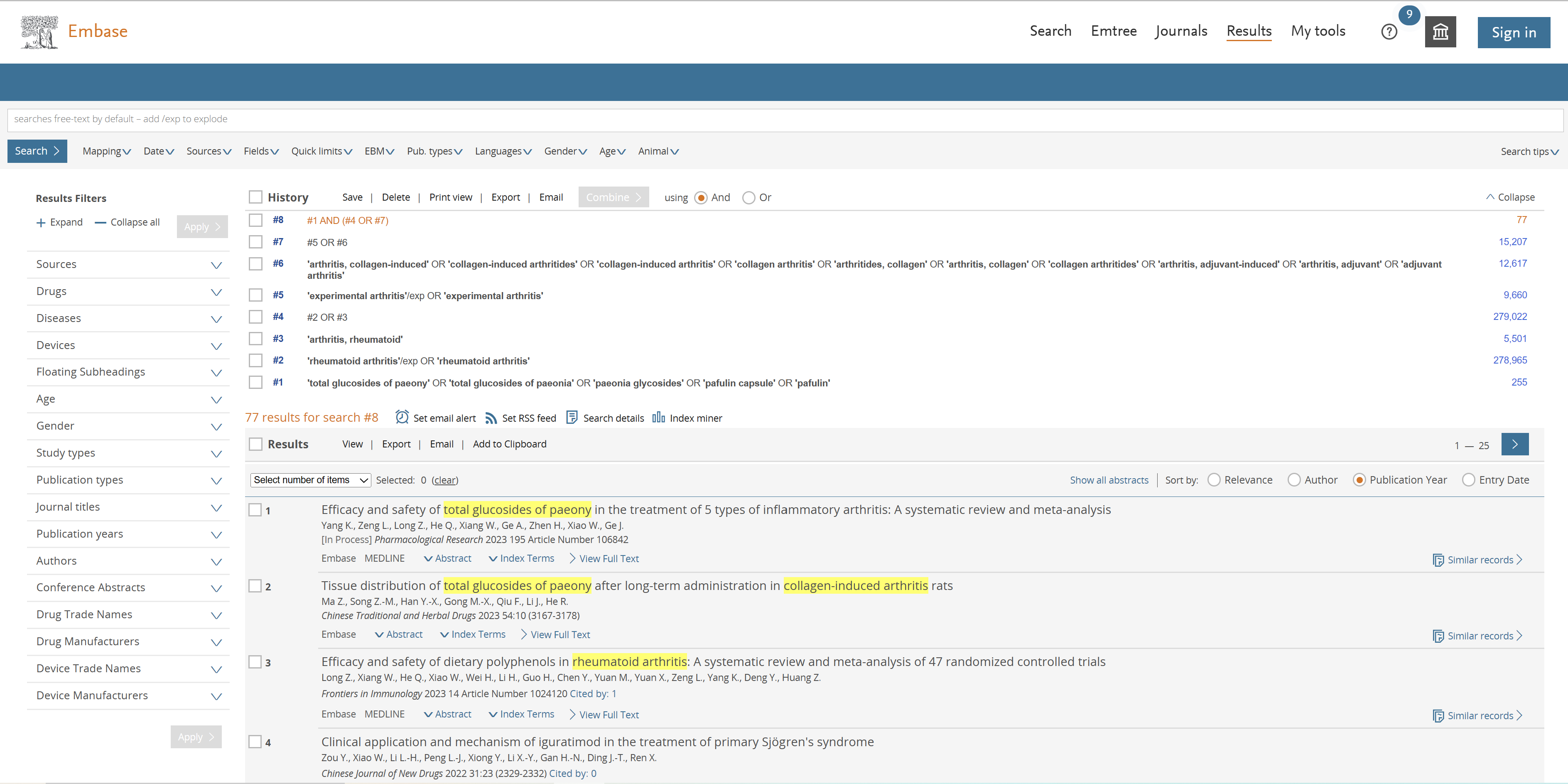


## 1.6 Cochrane Library (13)

The database search in Cochrane Library was carried out on August 14, 2023, and a total of 15 studies were found, but only export 13 Trials.


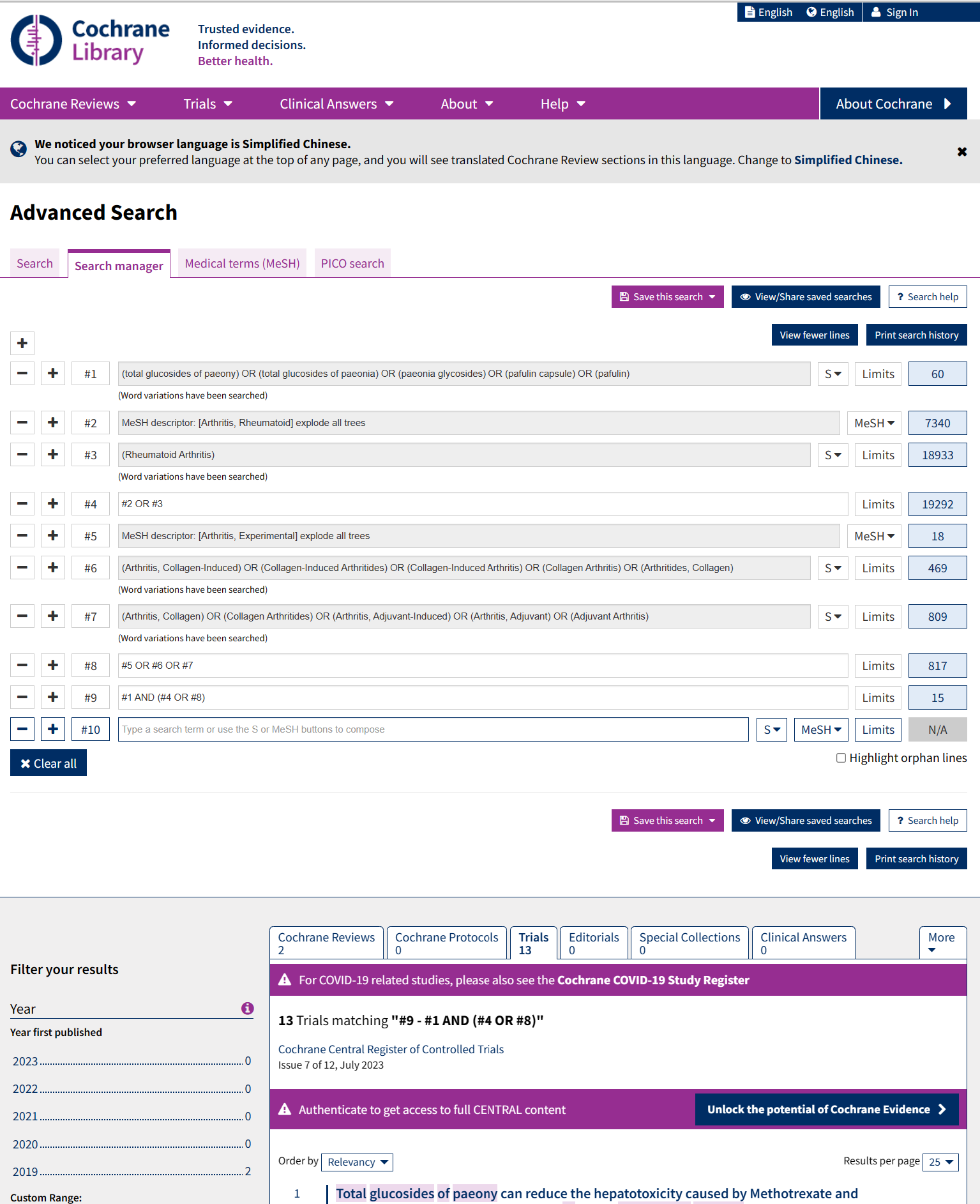


# Supporting information for data analysis

## 2.1 Forest plot of TNF-α after 1-2 weeks of TGP intervention


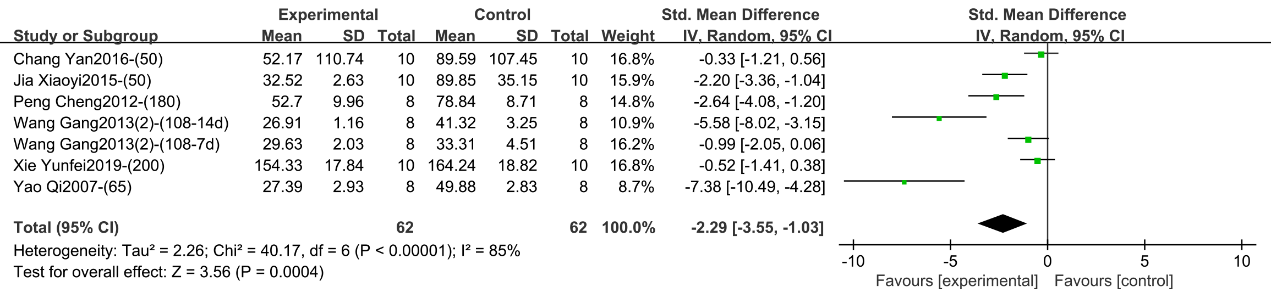


## 2.2 Sensitivity analysis of TNF-α after 1-2 weeks of TGP intervention


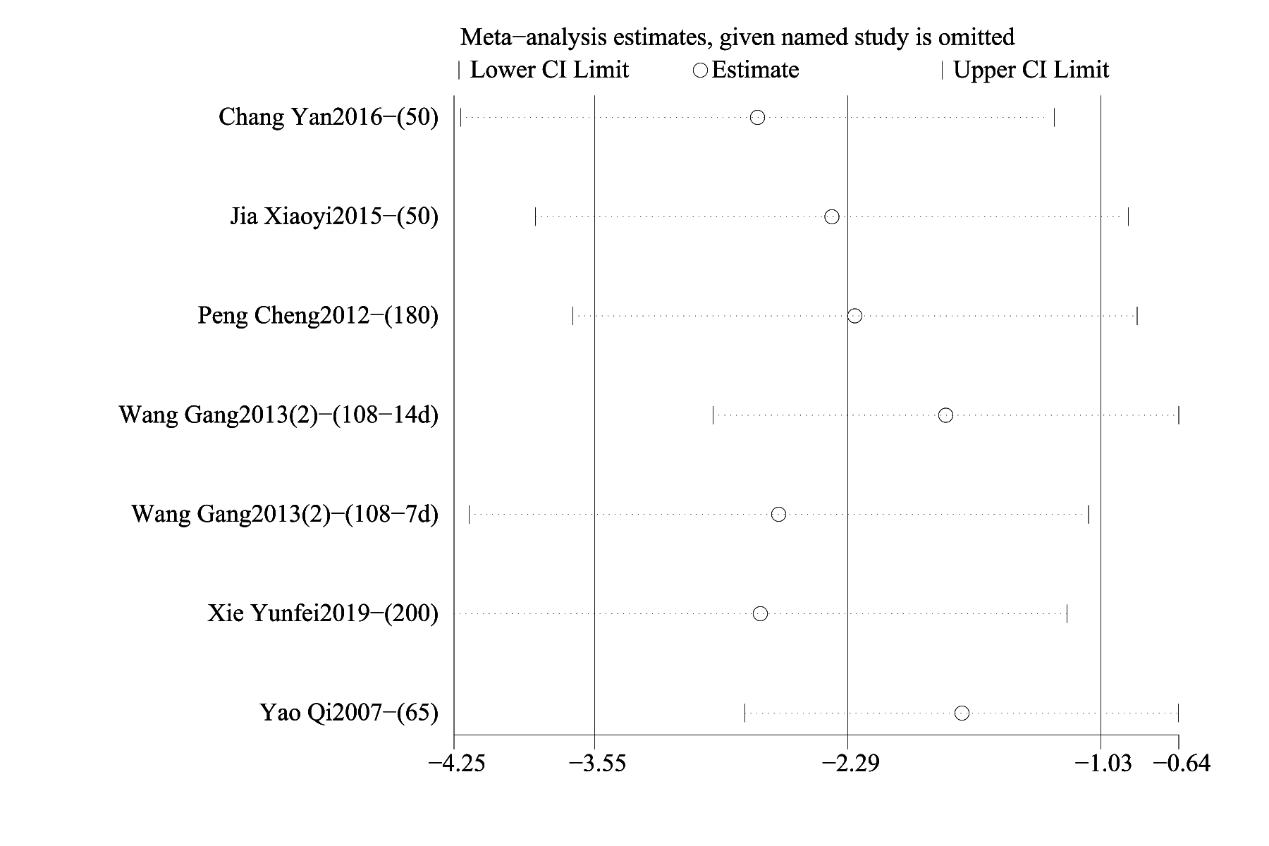


## 2.3 Subgroup analysis of TNF-α after 1-2 weeks of TGP intervention according to doses


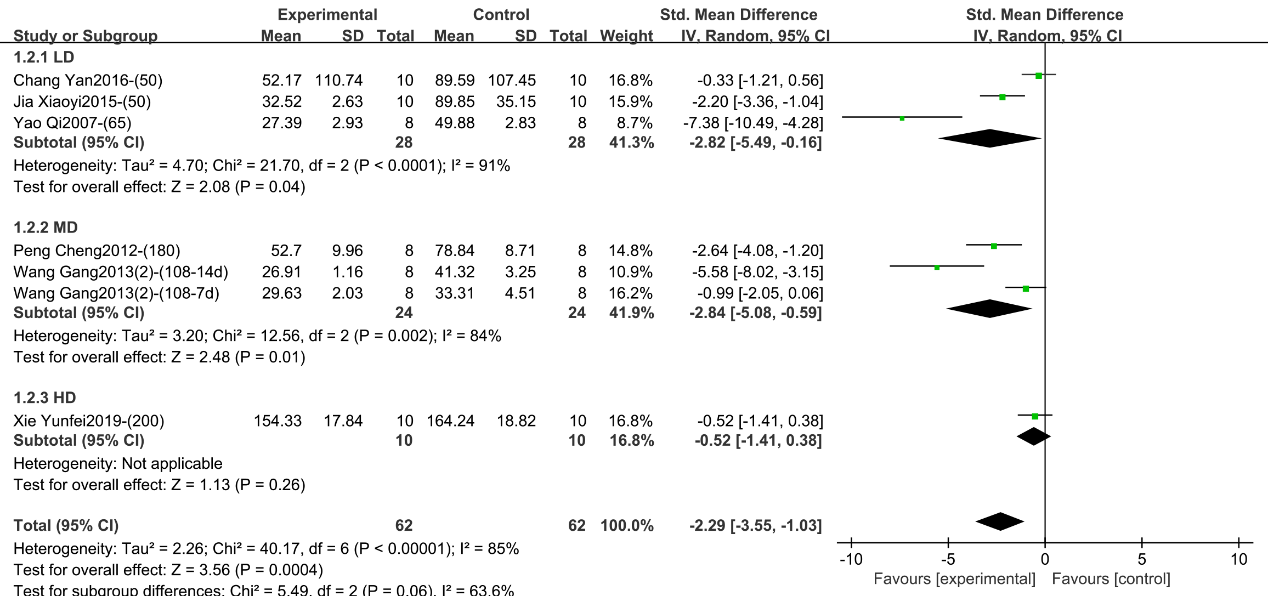


## 2.4 Subgroup analysis of TNF-α after 1-2 weeks of TGP intervention according to animal model types


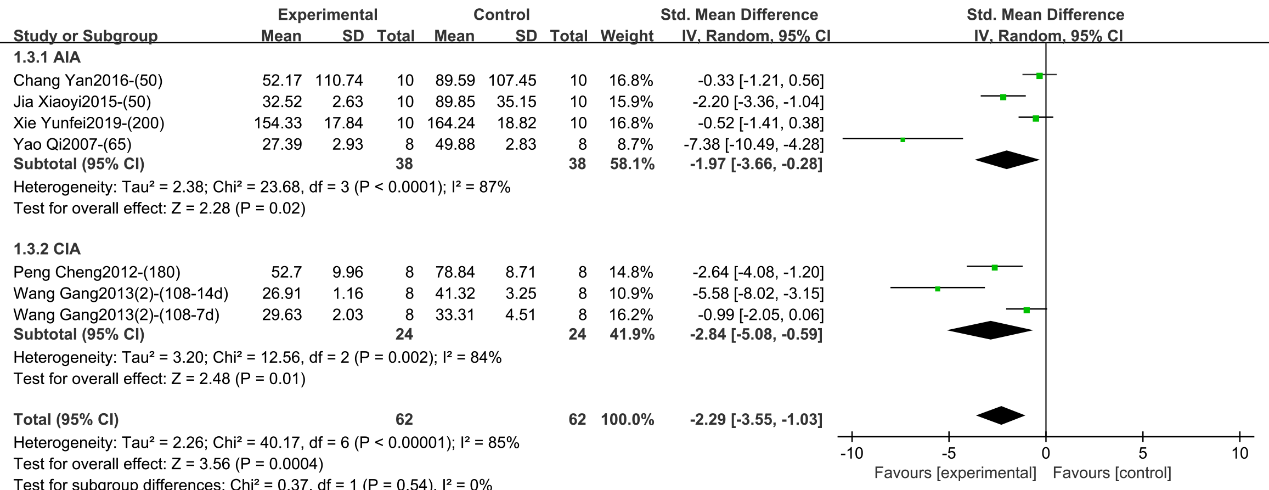


## 2.5 Subgroup analysis of TNF-α after 1-2 weeks of TGP intervention according to TGP 's manufacturers


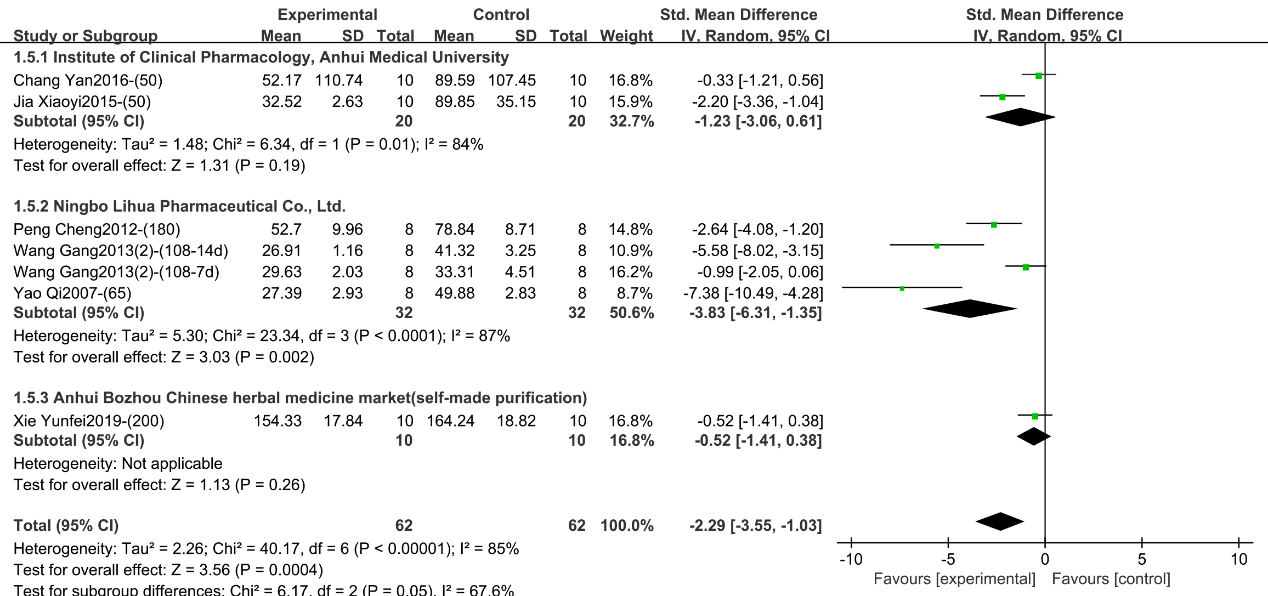


## 2.6 Subgroup analysis of TNF-α after 1-2 weeks of TGP intervention according to animal strains


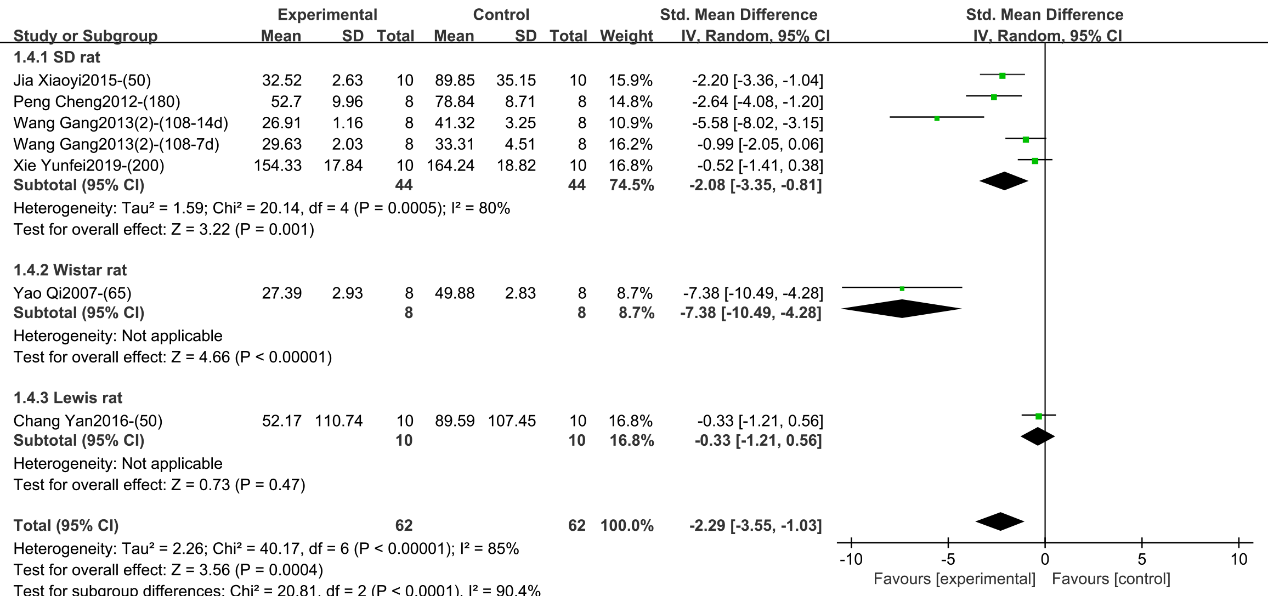


## 2.7 Forest plot of TNF-α after 3-4 weeks of TGP intervention


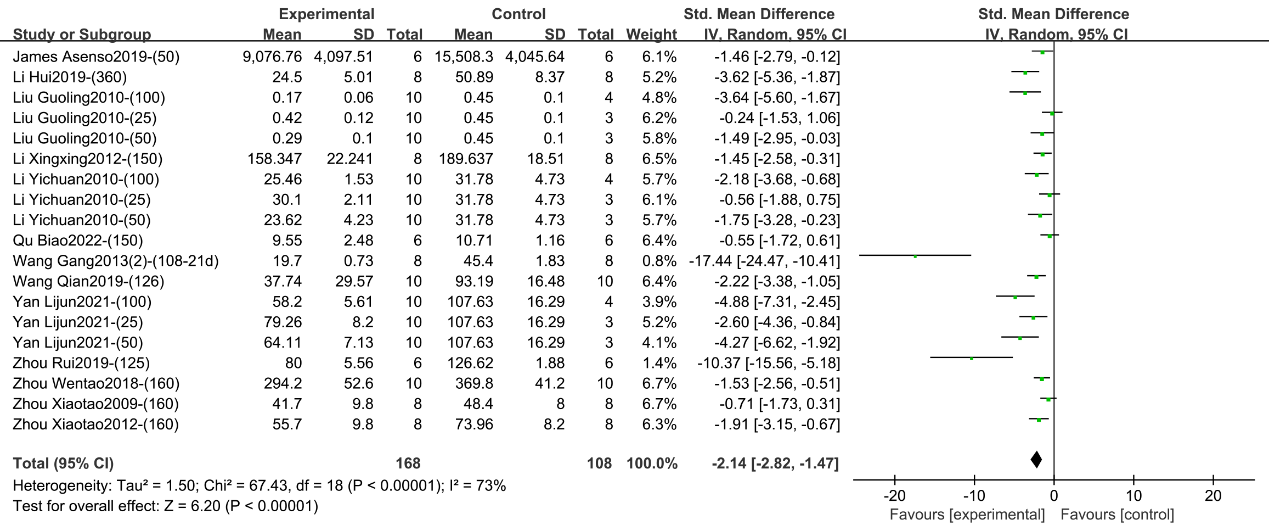


## 2.8 Sensitivity analysis of TNF-α after 3-4 weeks of TGP intervention


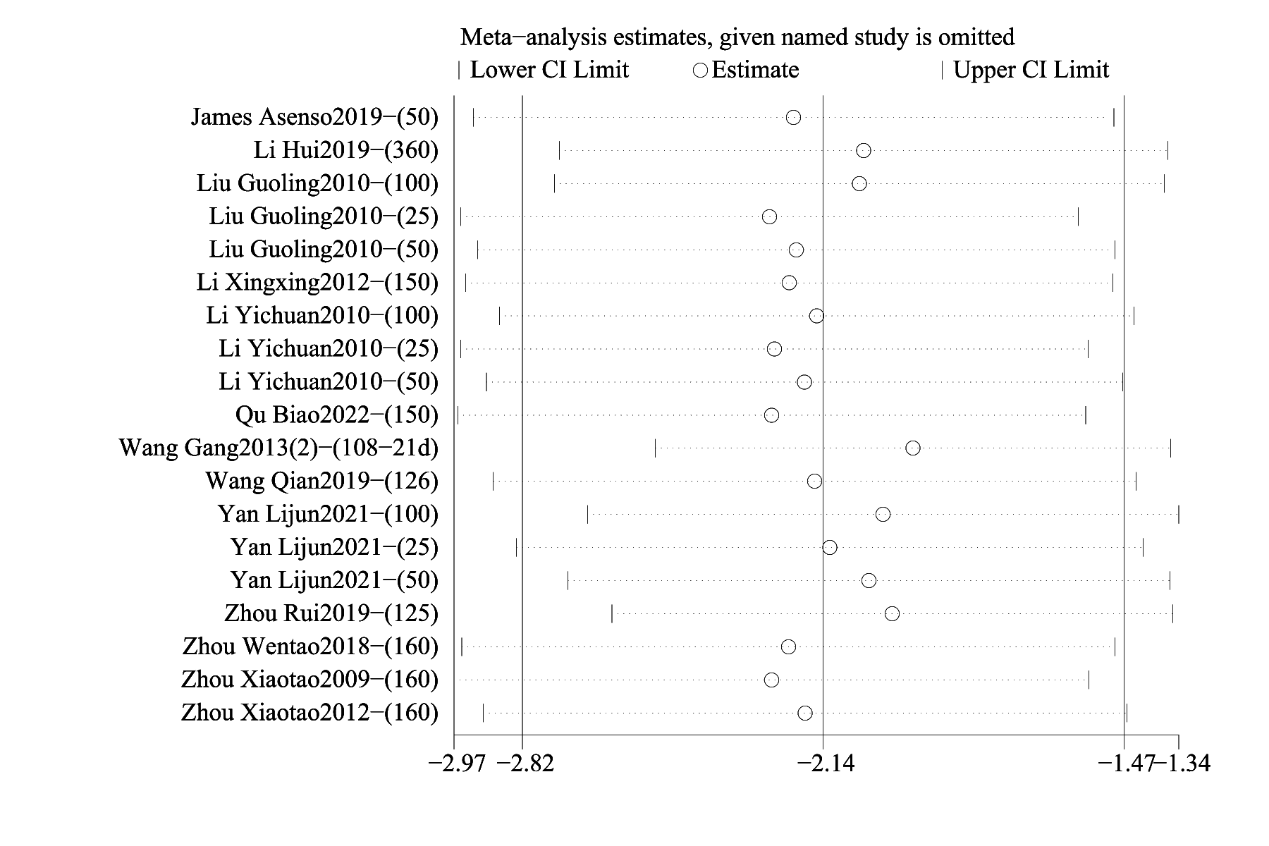


## 2.9 Subgroup analysis of TNF-α after 3-4 weeks of TGP intervention according to doses


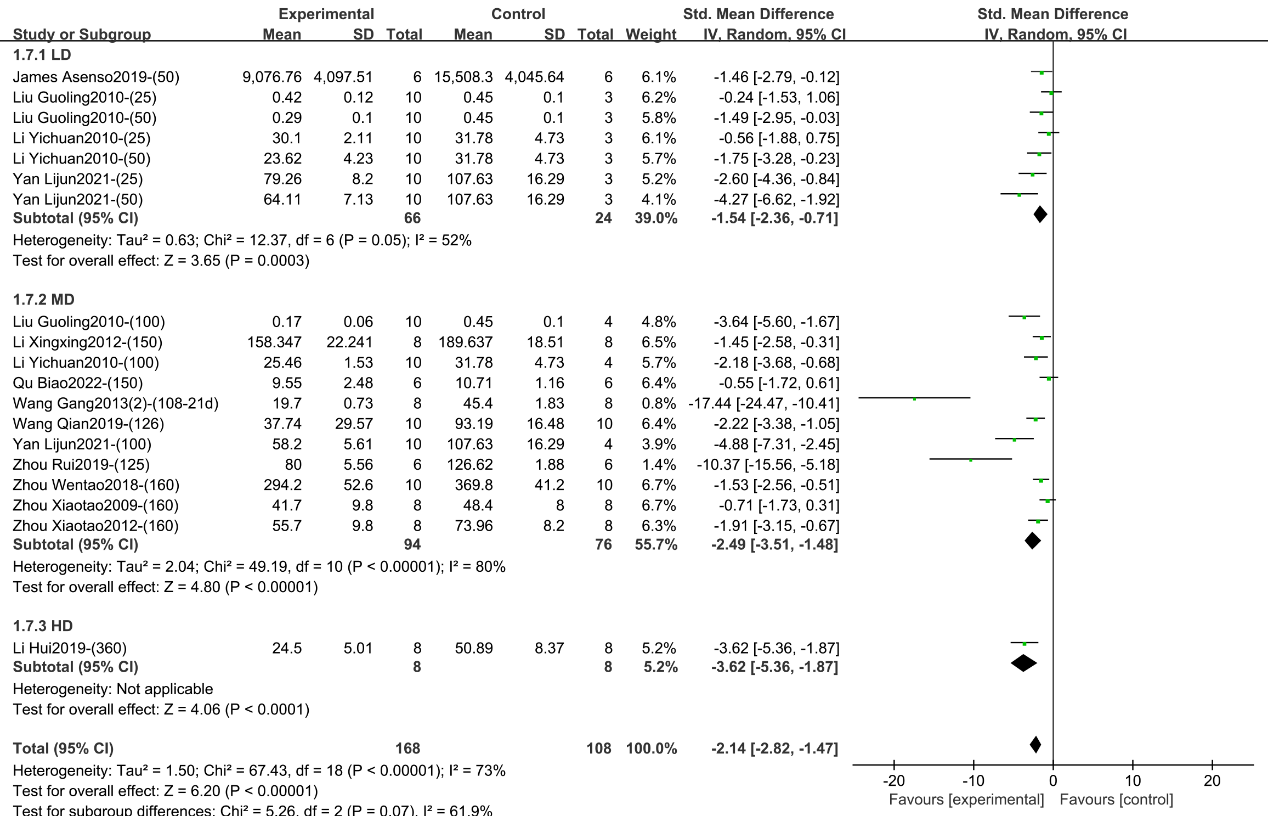


## 2.10 Subgroup analysis of TNF-α after 3-4 weeks of TGP intervention according to animal model types


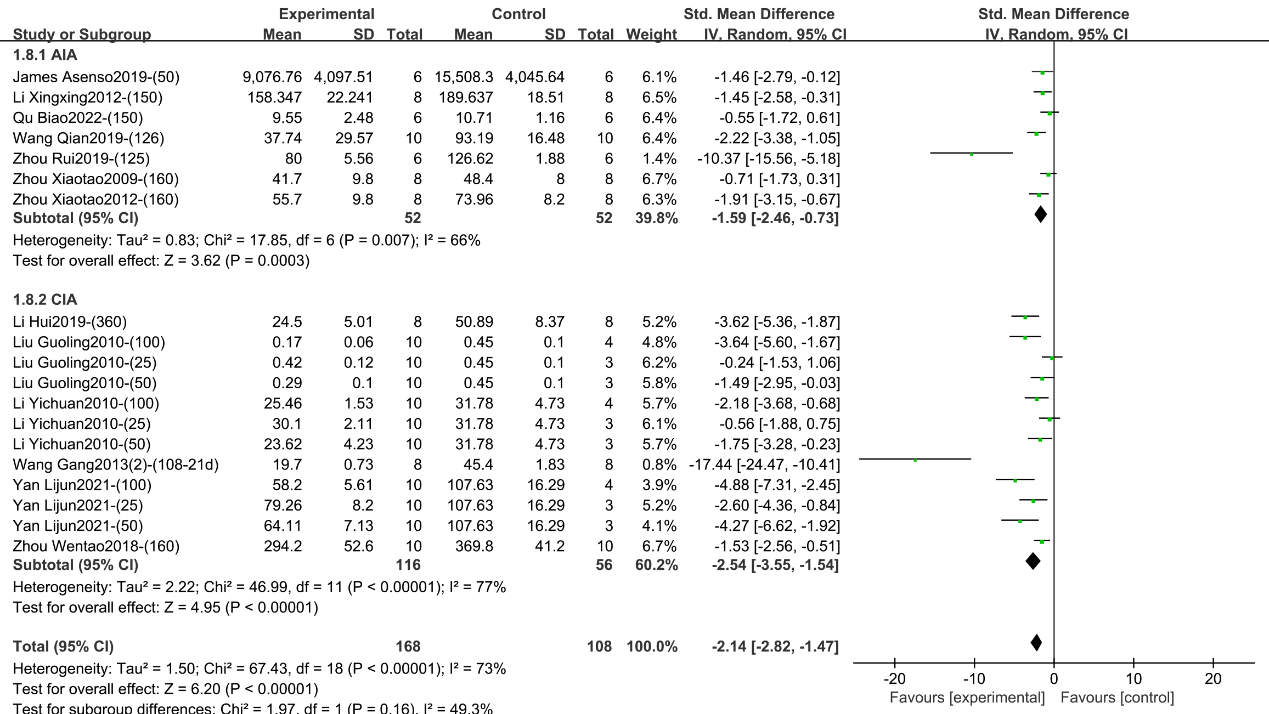


## 2.11 Subgroup analysis of TNF-α after 3-4 weeks of TGP intervention according to animal species


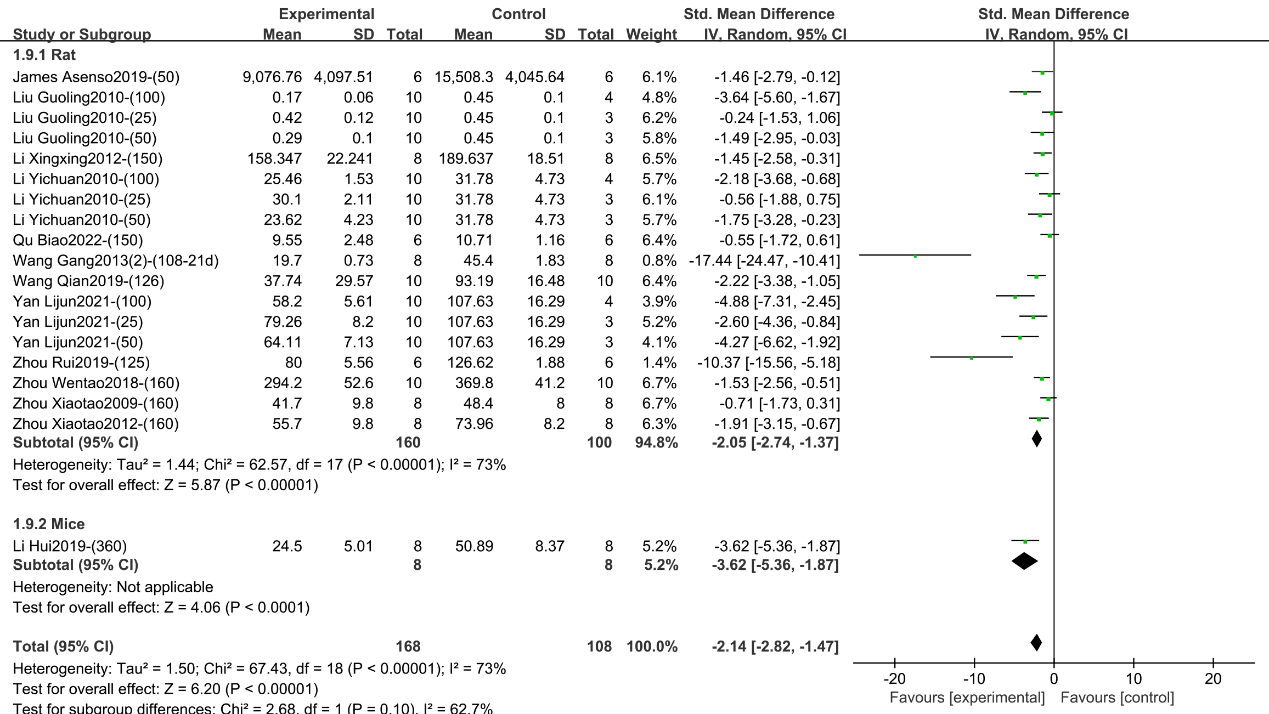


## 2.12 Subgroup analysis of TNF-α after 3-4 weeks of TGP intervention according to animal strains


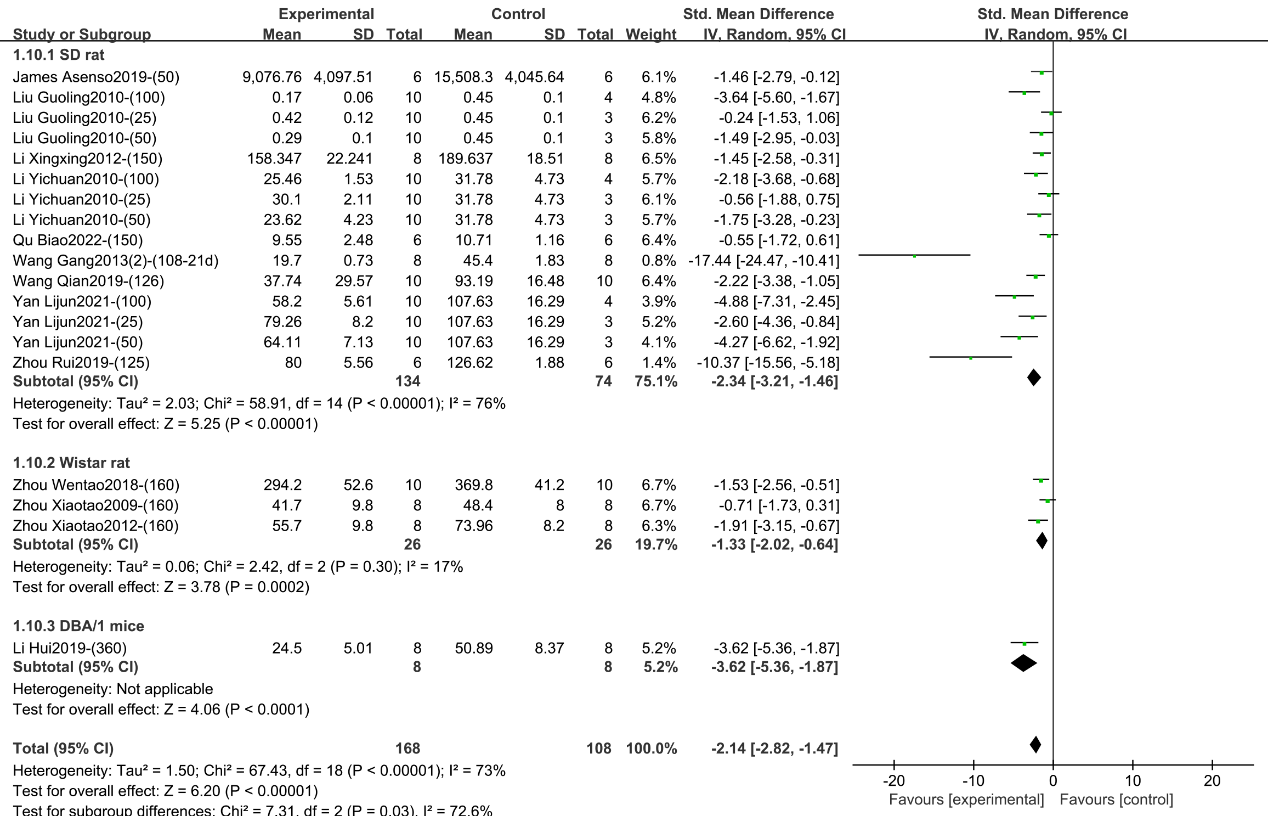


## 2.13 Funnel plot of TNF-α after 3-4 weeks of TGP intervention


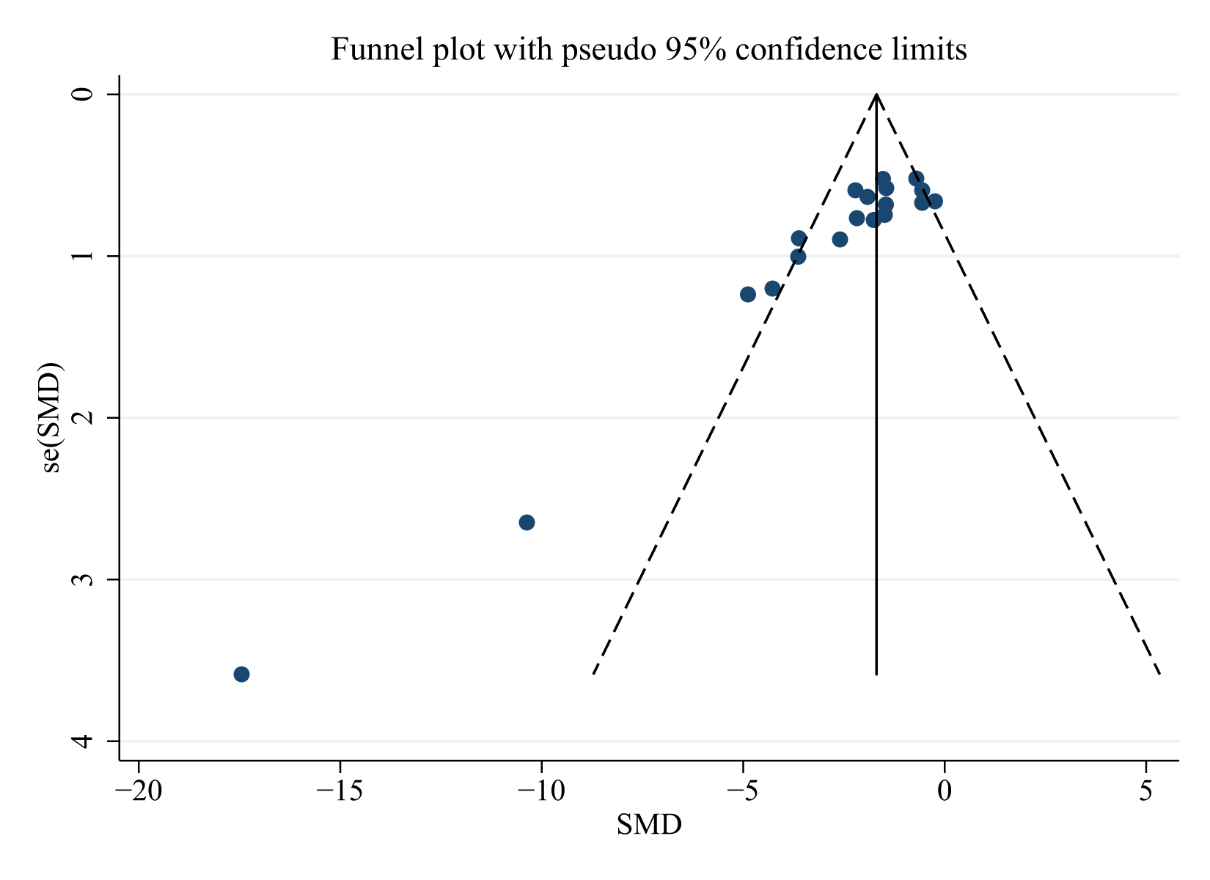


## 2.14 Forest plot of TNF-α after 8 weeks of TGP intervention


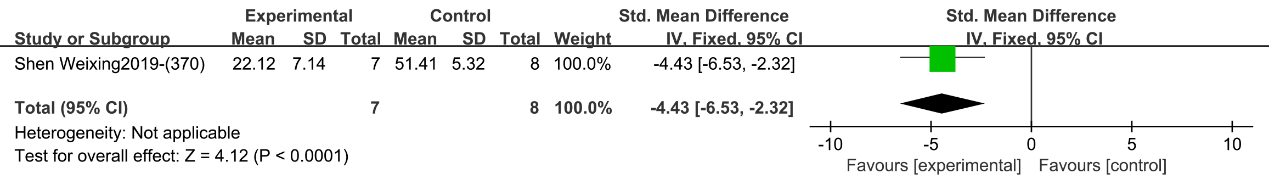


## 2.15 Forest plot of IL-1β after 1-2 weeks of TGP intervention


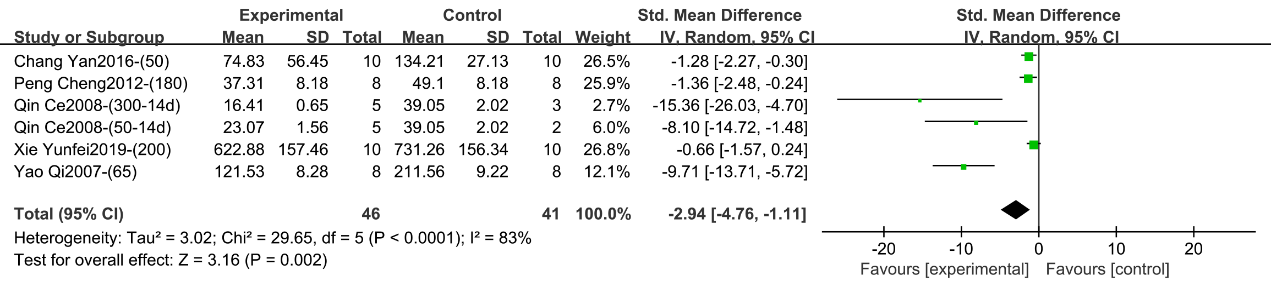


## 2.16 Sensitivity analysis of IL-1β after 1-2 weeks of TGP intervention


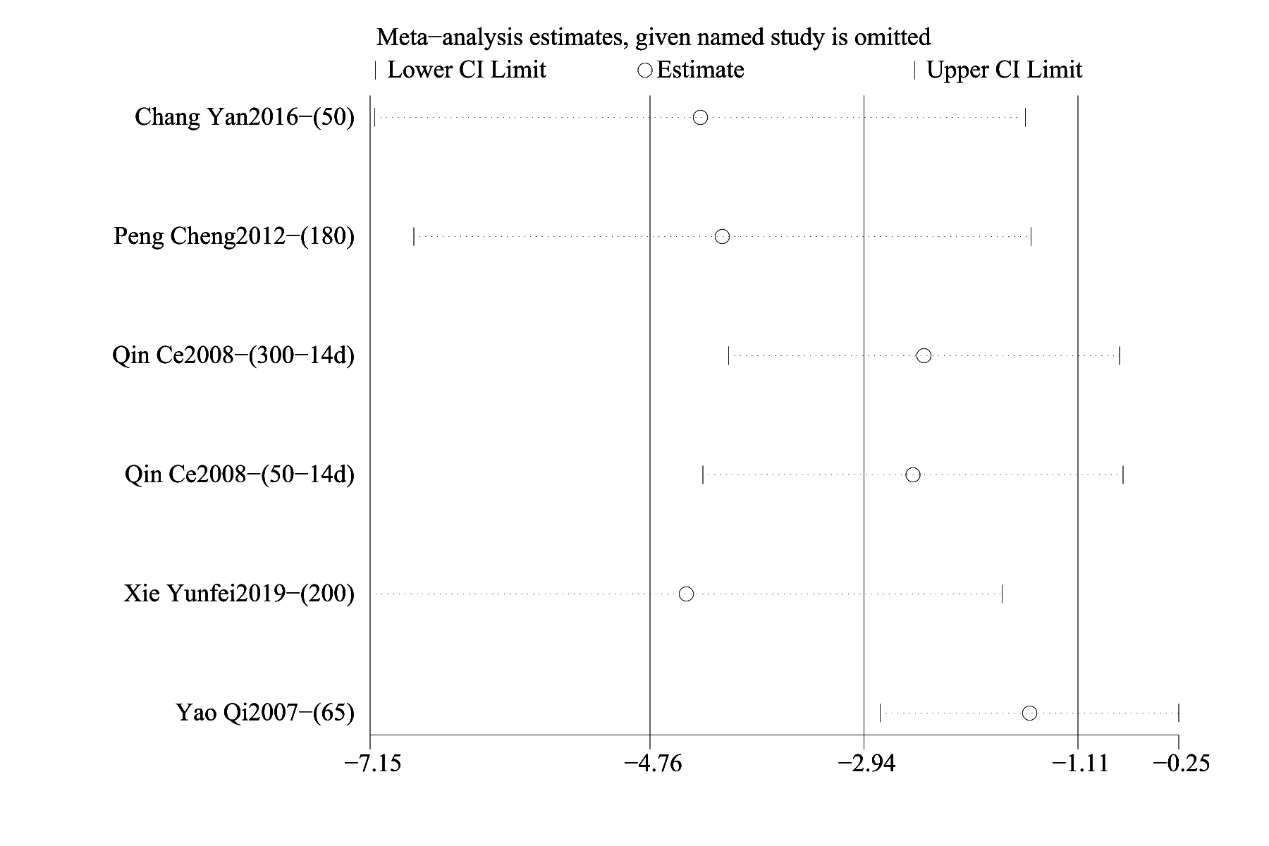


## 2.17 Subgroup analysis of IL-1β after 1-2 weeks of TGP intervention according to doses


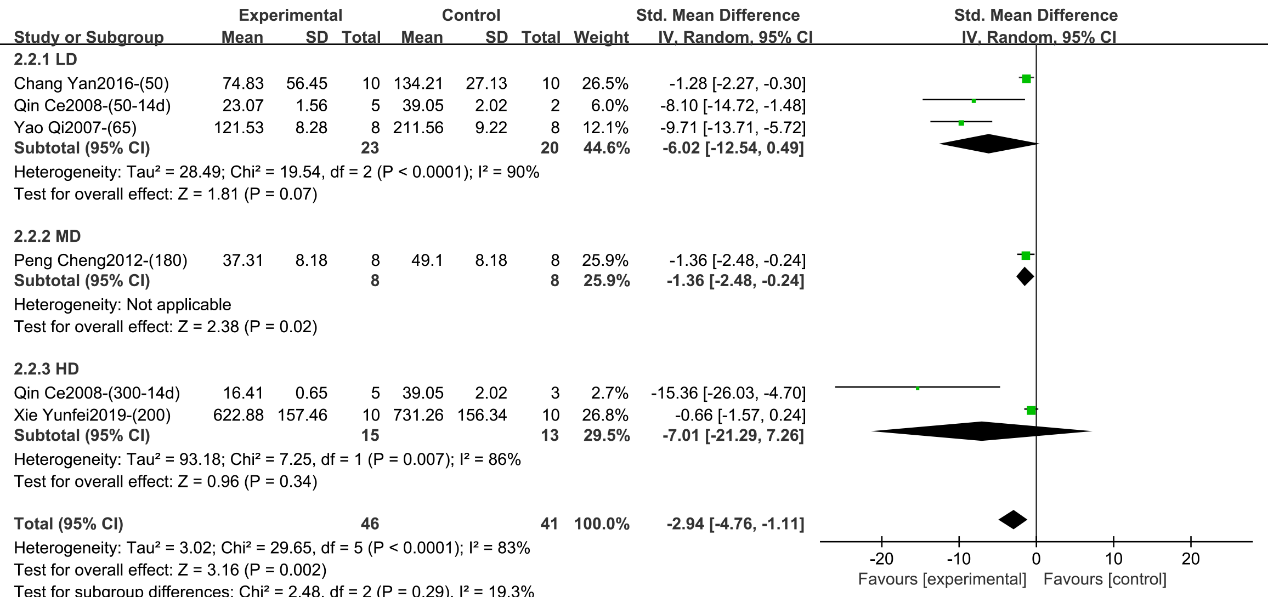


## 2.18 Subgroup analysis of IL-1β after 1-2 weeks of TGP intervention according to animal model types


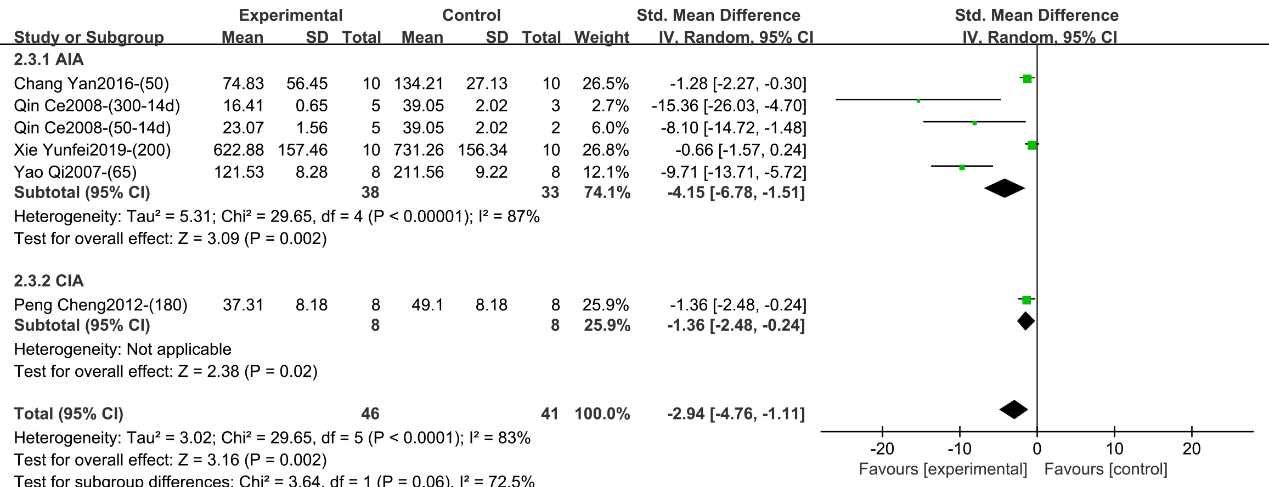


## 2.19 Subgroup analysis of IL-1β after 1-2 weeks of TGP intervention according to TGP 's manufacturers


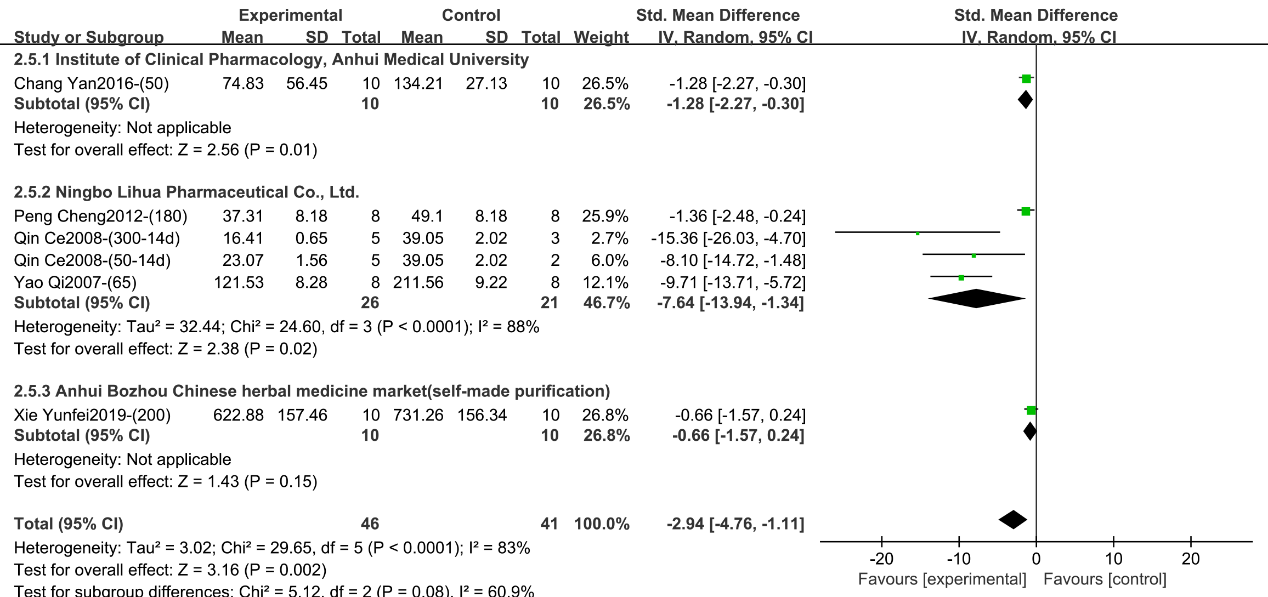


## 2.20 Subgroup analysis of IL-1β after 1-2 weeks of TGP intervention according to animal strains


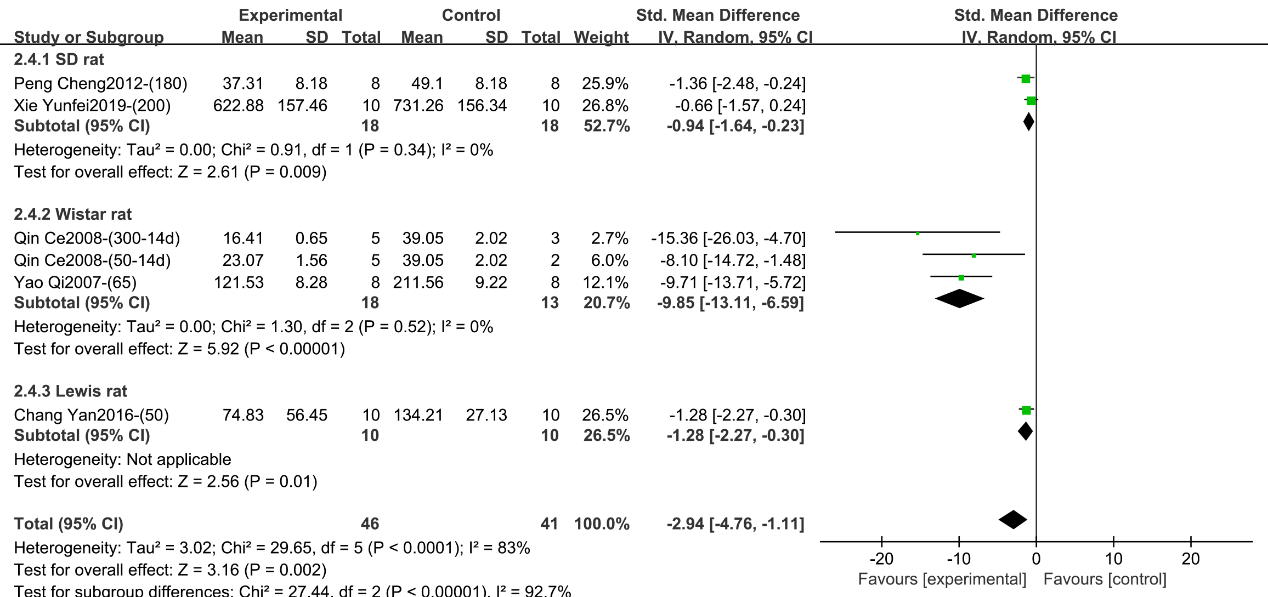


## 2.21 Forest plot of IL-1β after 3-4 weeks of TGP intervention


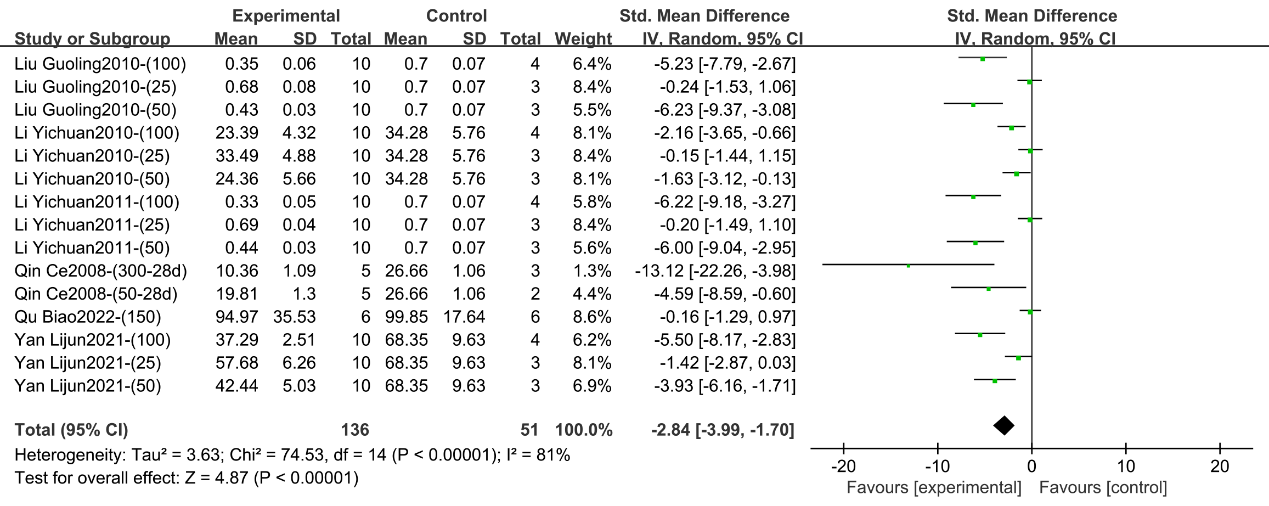


## 2.22 Sensitivity analysis of IL-1β after 3-4 weeks of TGP intervention


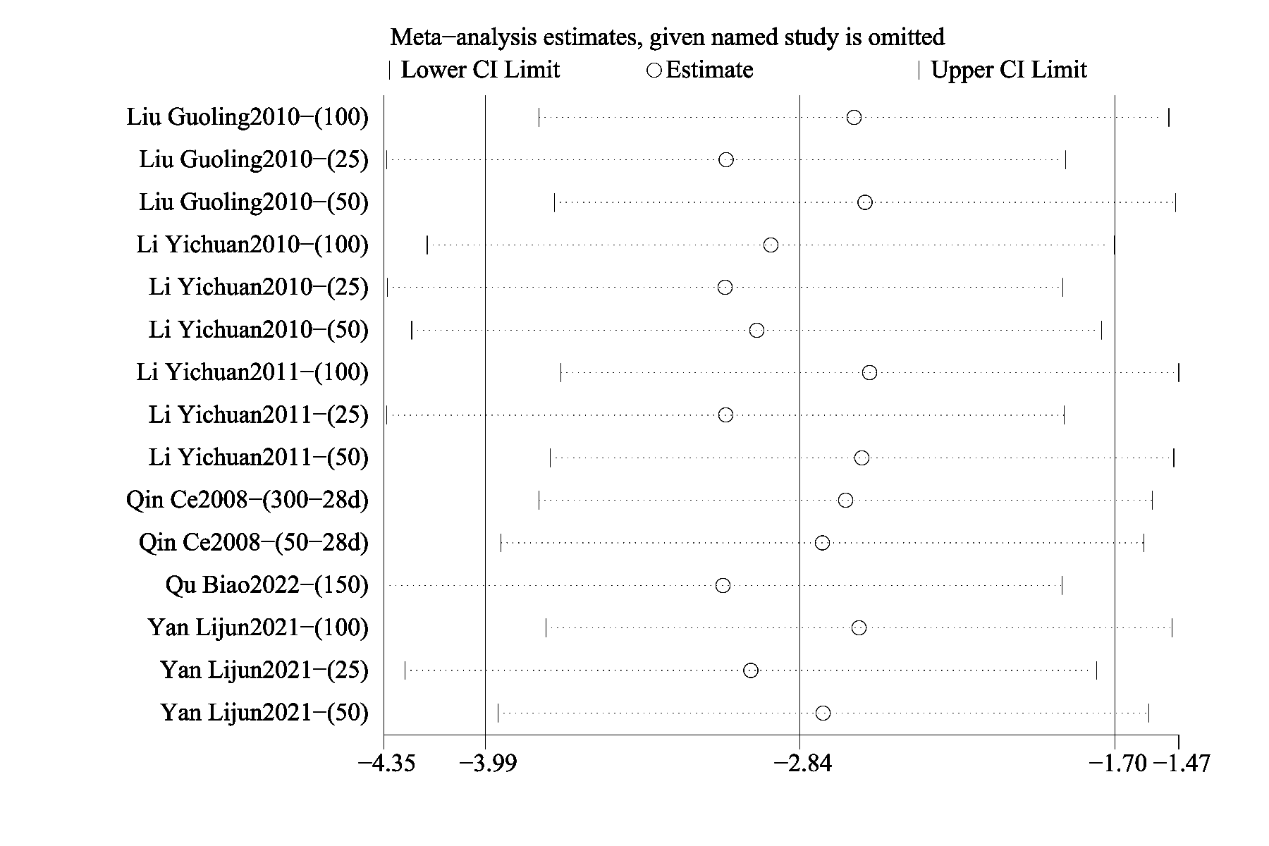


## 2.23 Subgroup analysis of IL-1β after 3-4 weeks of TGP intervention according to animal model types


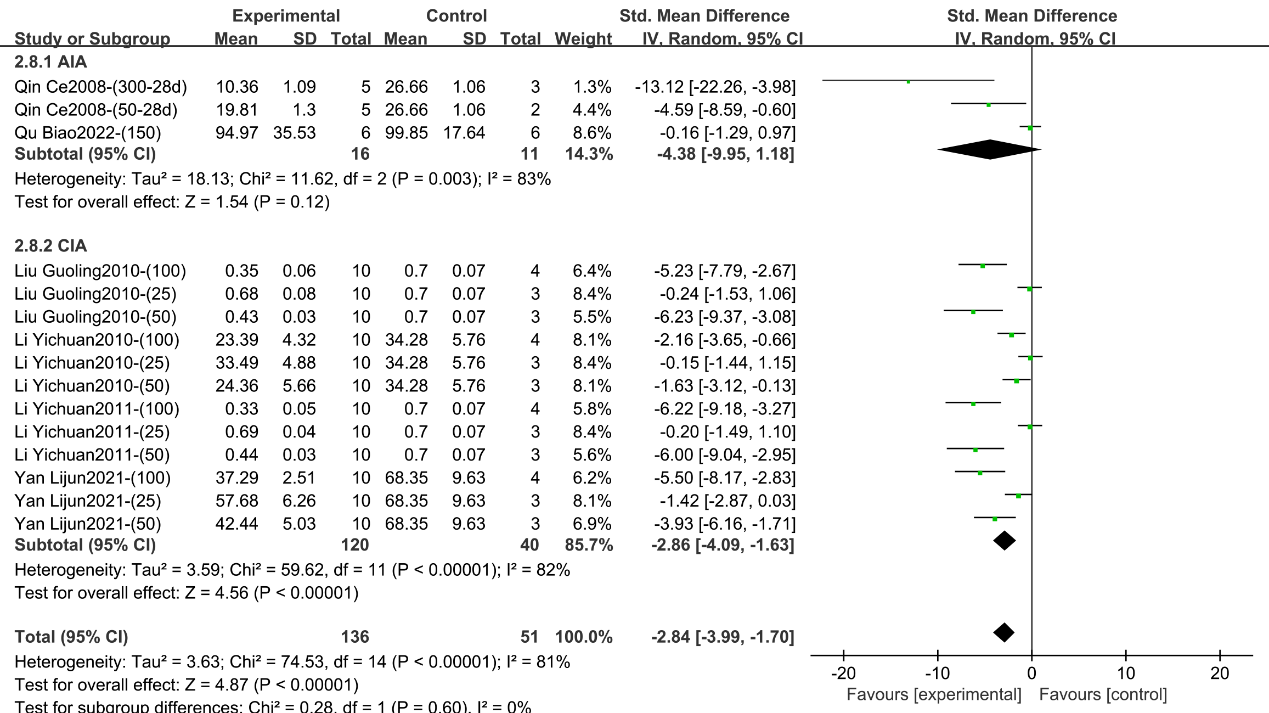


## 2.24 Subgroup analysis of IL-1β after 3-4 weeks of TGP intervention according to animal strains


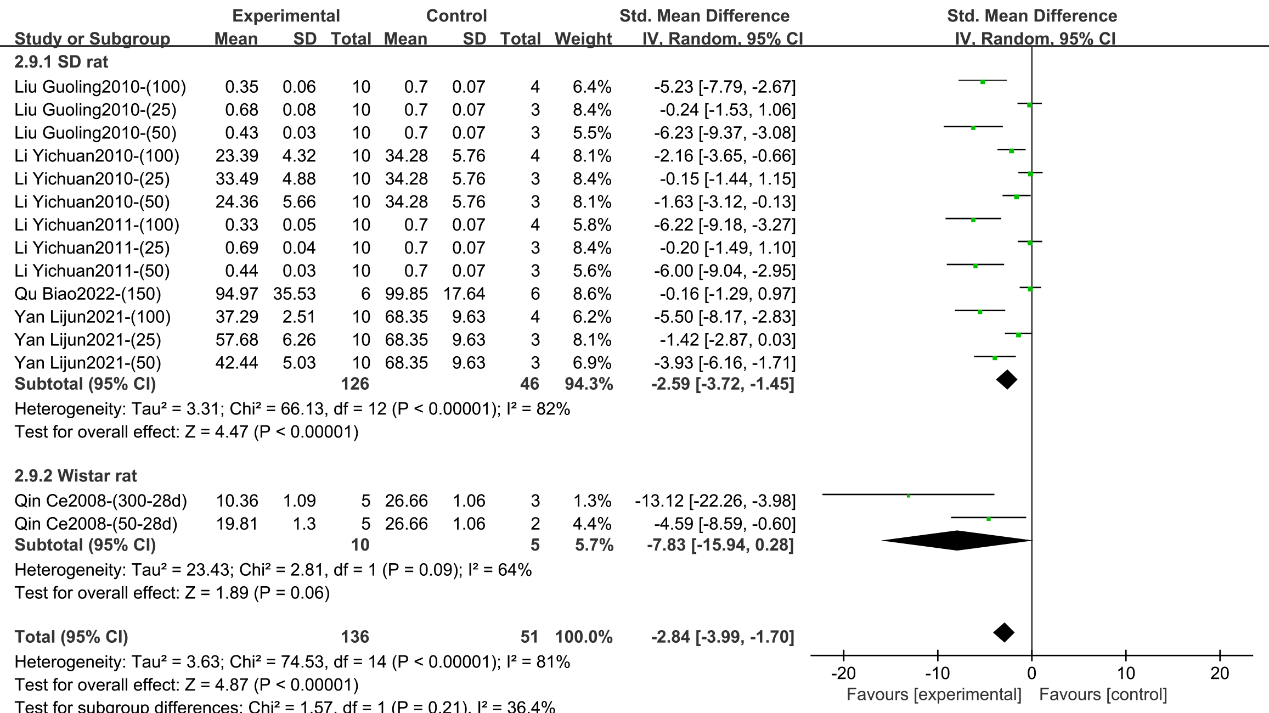


## 2.25 Subgroup analysis of IL-1β after 3-4 weeks of TGP intervention according to doses


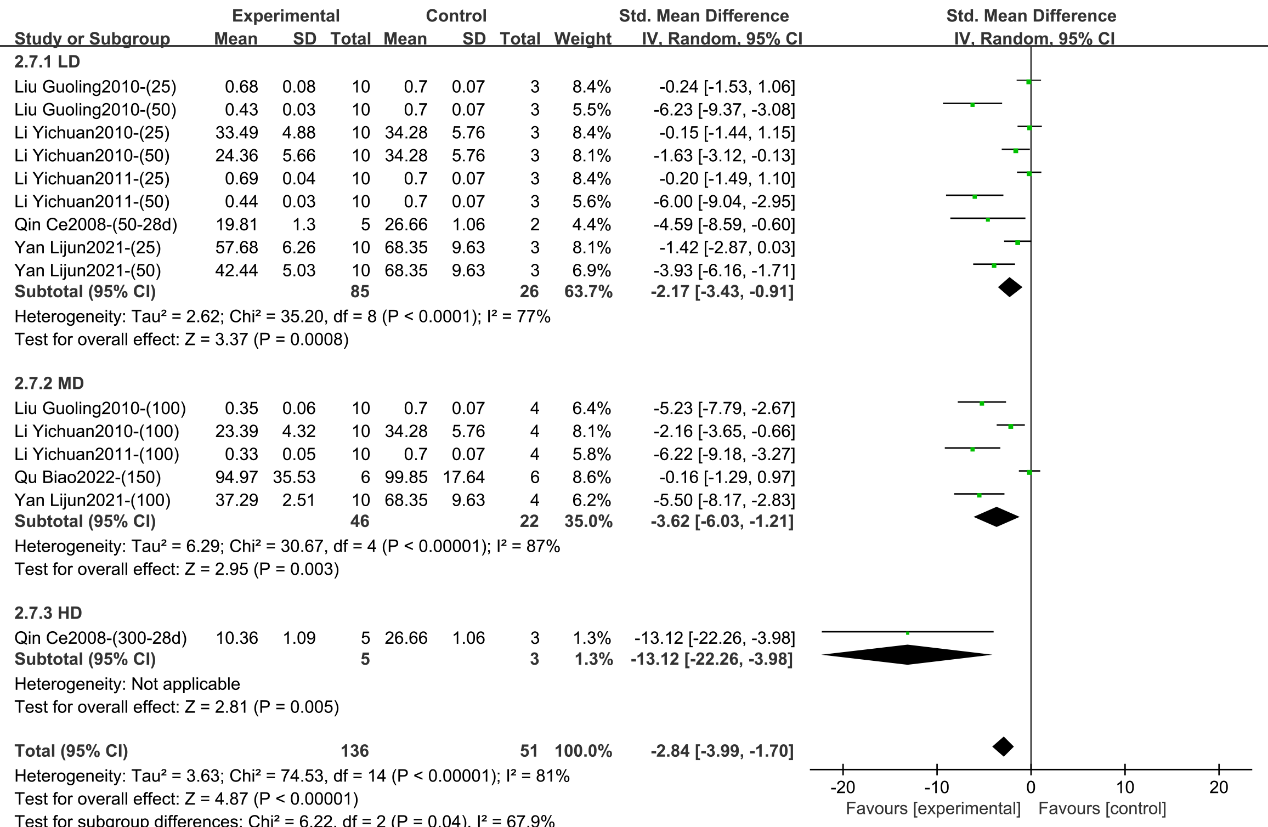


## 2.26 Subgroup analysis of IL-1β after 3-4 weeks of TGP intervention according to TGP 's manufacturers


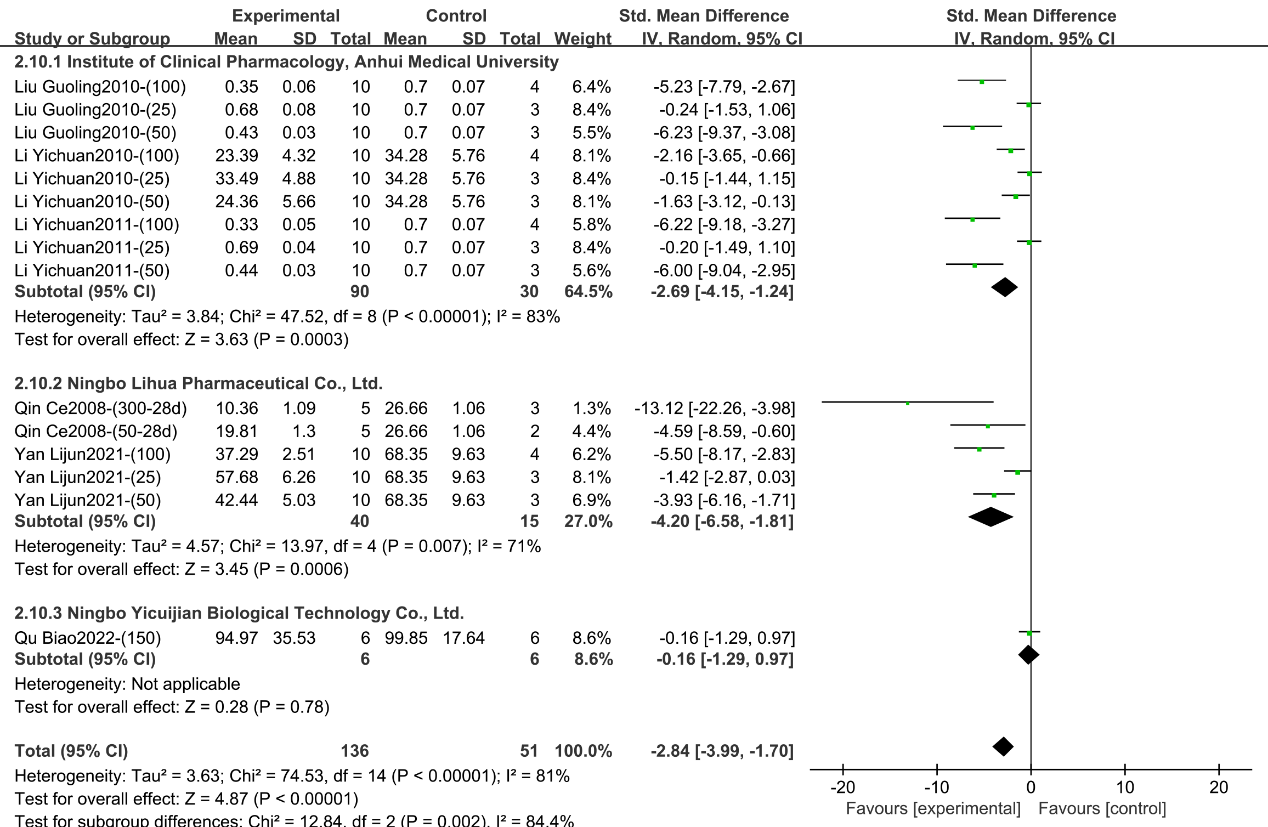


## 2.27 Funnel plot of IL-1β after 3-4 weeks of TGP intervention


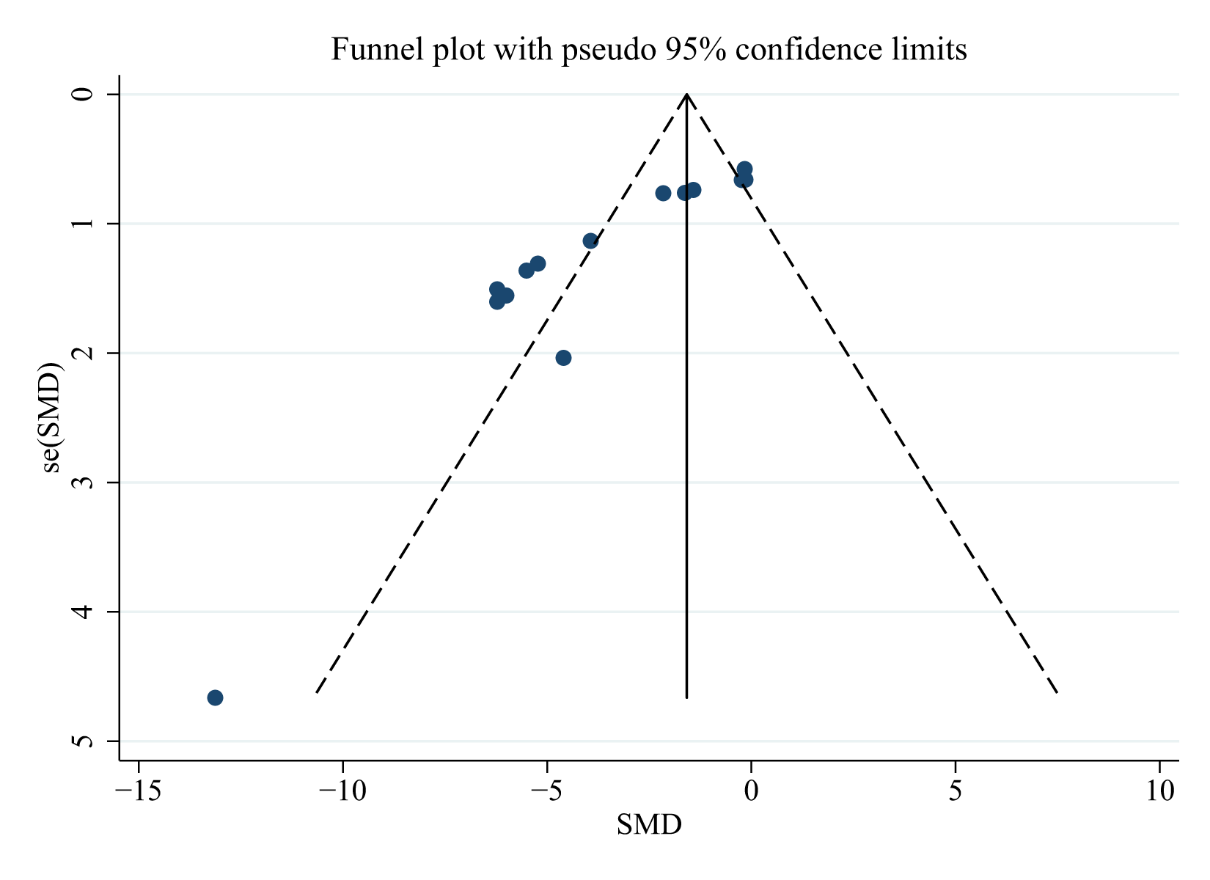


## 2.28 Forest plot of IL-6 after 1-2 weeks of TGP intervention


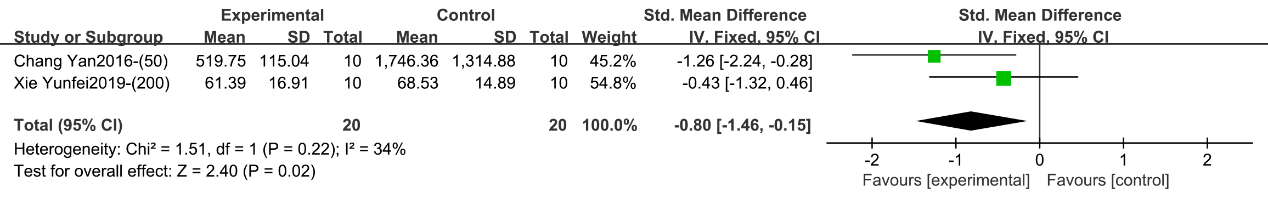


## 2.29 Forest plot of IL-6 after 3-4 weeks of TGP intervention


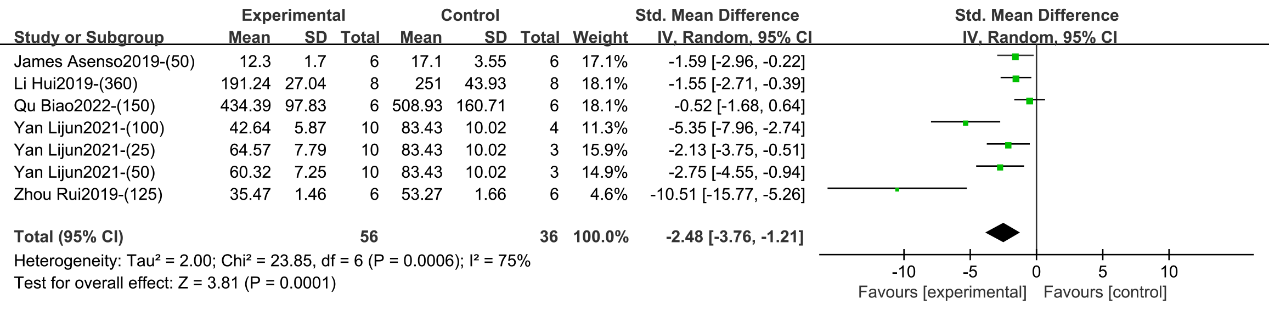


## 2.30 Sensitivity analysis of IL-6 after 3-4 weeks of TGP intervention


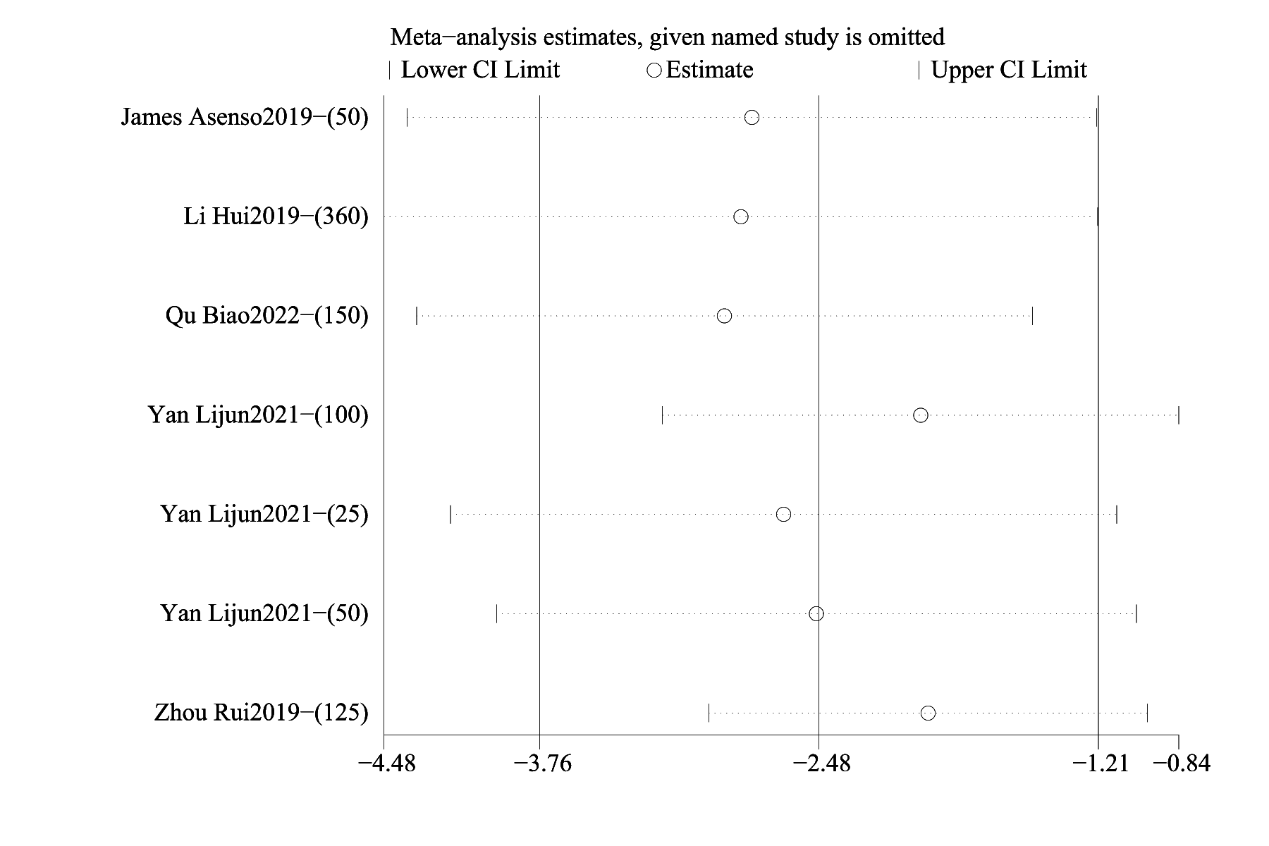


## 2.31 Subgroup analysis of IL-6 after 3-4 weeks of TGP intervention according to doses


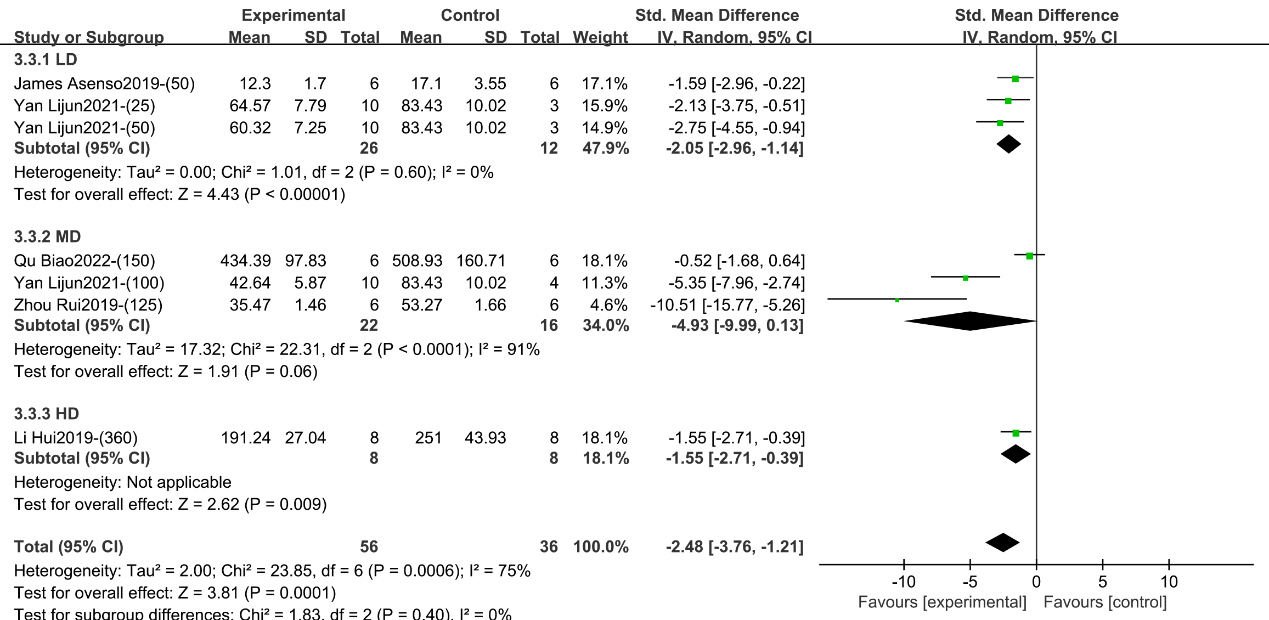


## 2.32 Subgroup analysis of IL-6 after 3-4 weeks of TGP intervention according to animal model types


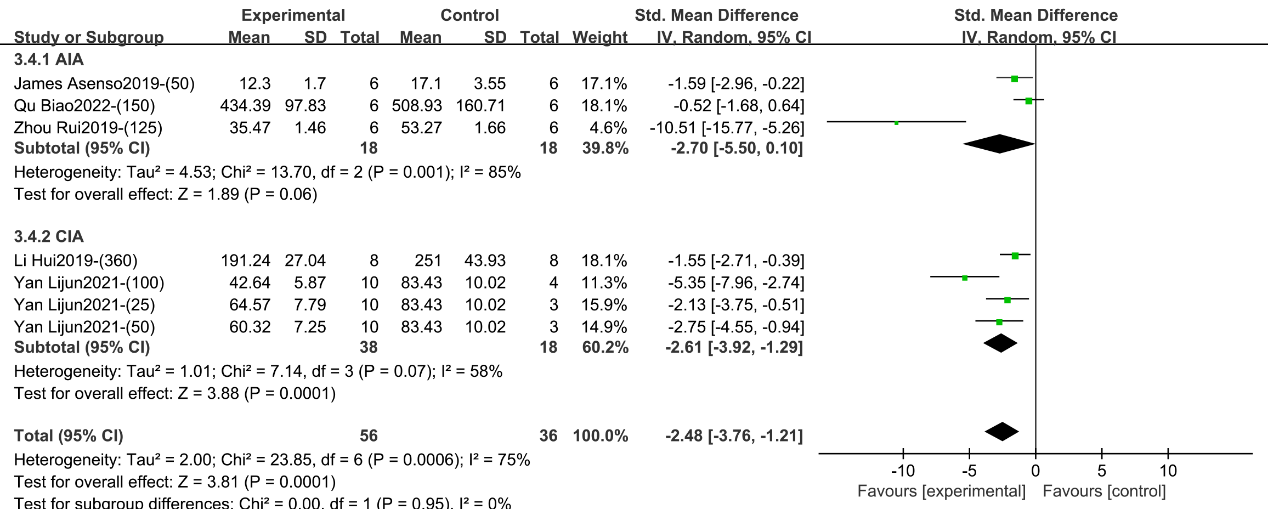


## 2.33 Subgroup analysis of IL-6 after 3-4 weeks of TGP intervention according to animal species


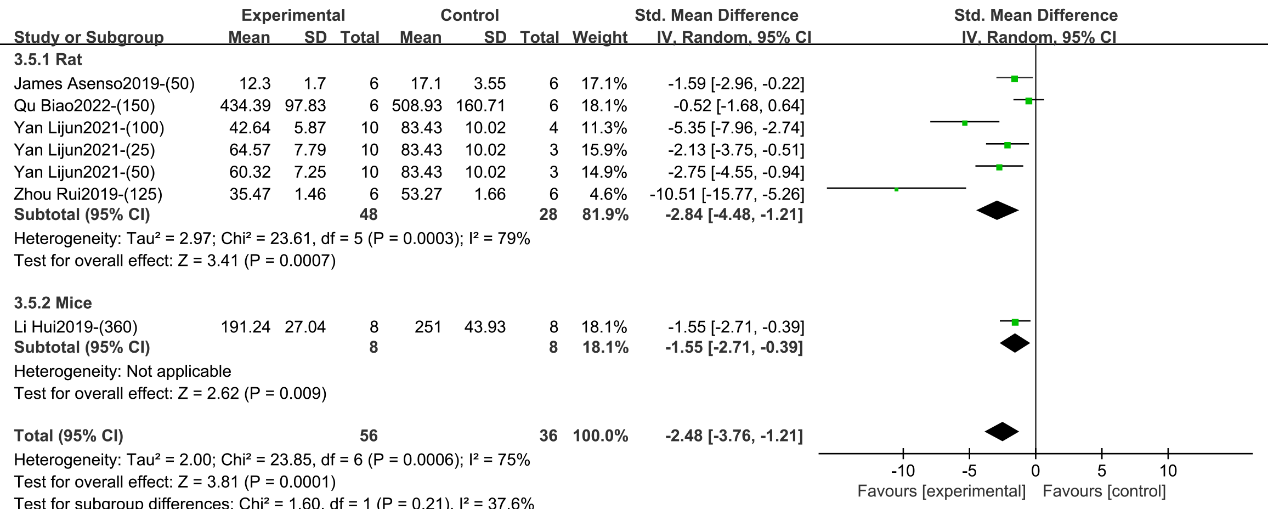


## 2.34 Subgroup analysis of IL-6 after 3-4 weeks of TGP intervention according to animal strains


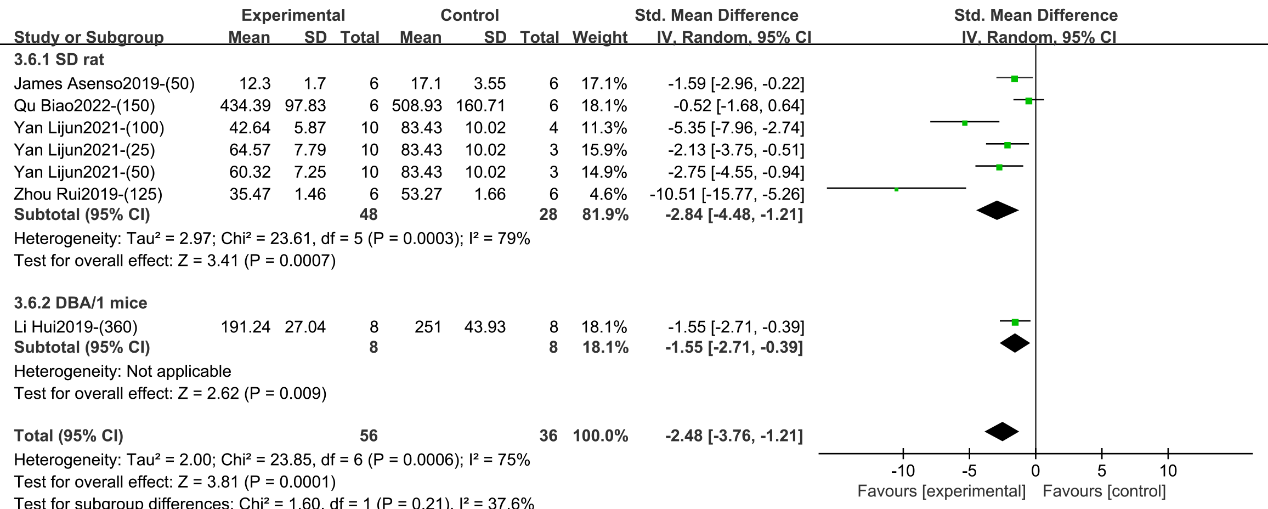


## 2.35 Subgroup analysis of IL-6 after 3-4 weeks of TGP intervention according to TGP 's manufacturers


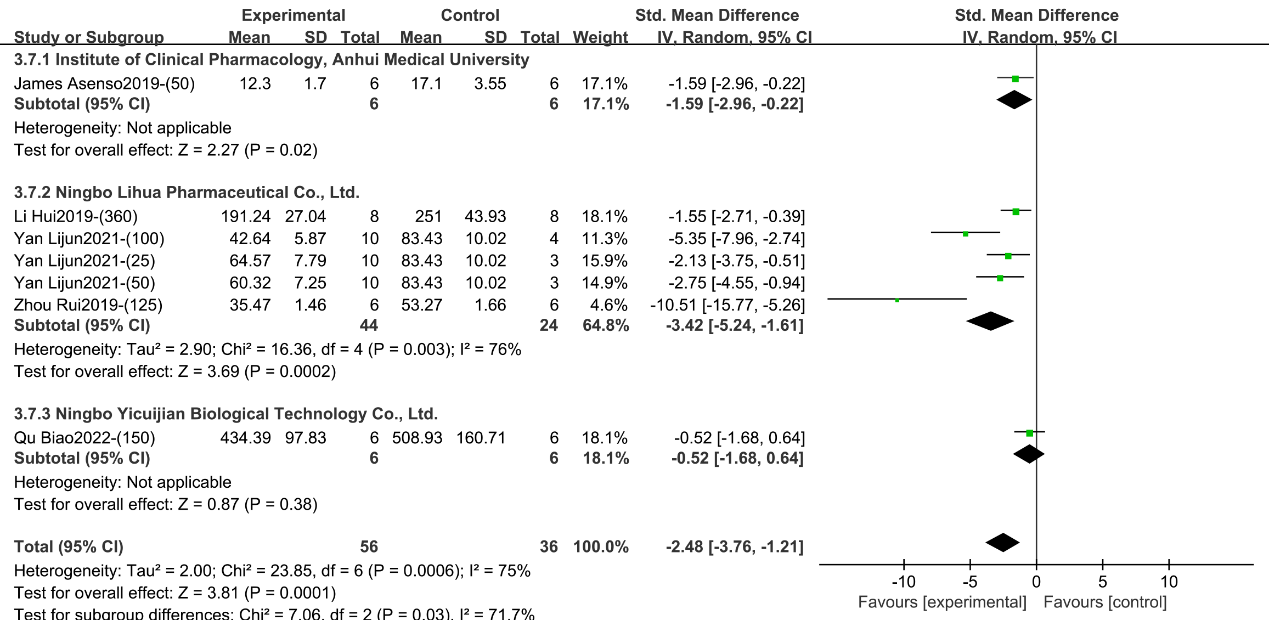


## 2.36 Forest plot of IL-6 after 8 weeks of TGP intervention


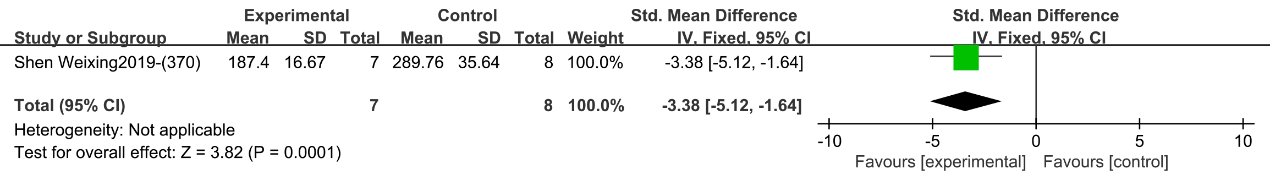


## 2.37 Forest plot of IL-10 after 1-2 weeks of TGP intervention


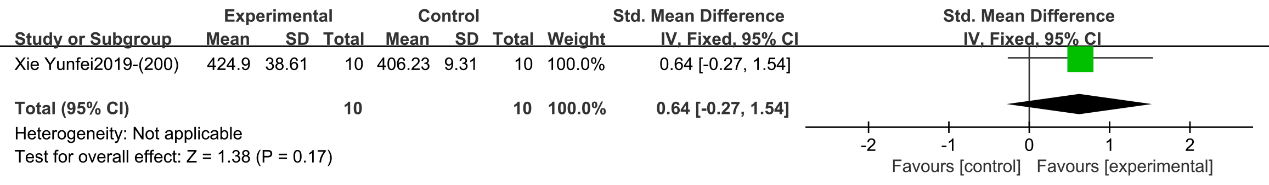


## 2.38 Forest plot of IL-10 after 3-4 weeks of TGP intervention


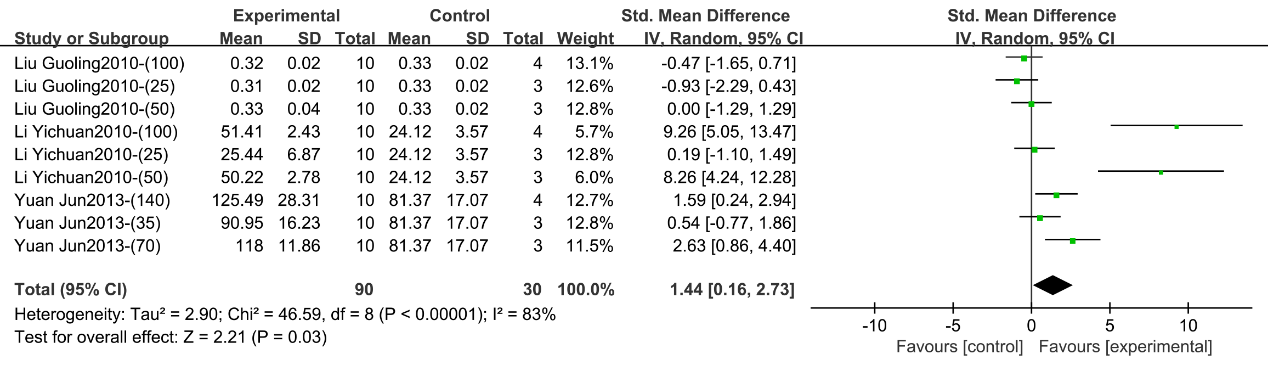


## 2.39 Sensitivity analysis of IL-10 after 3-4 weeks of TGP intervention


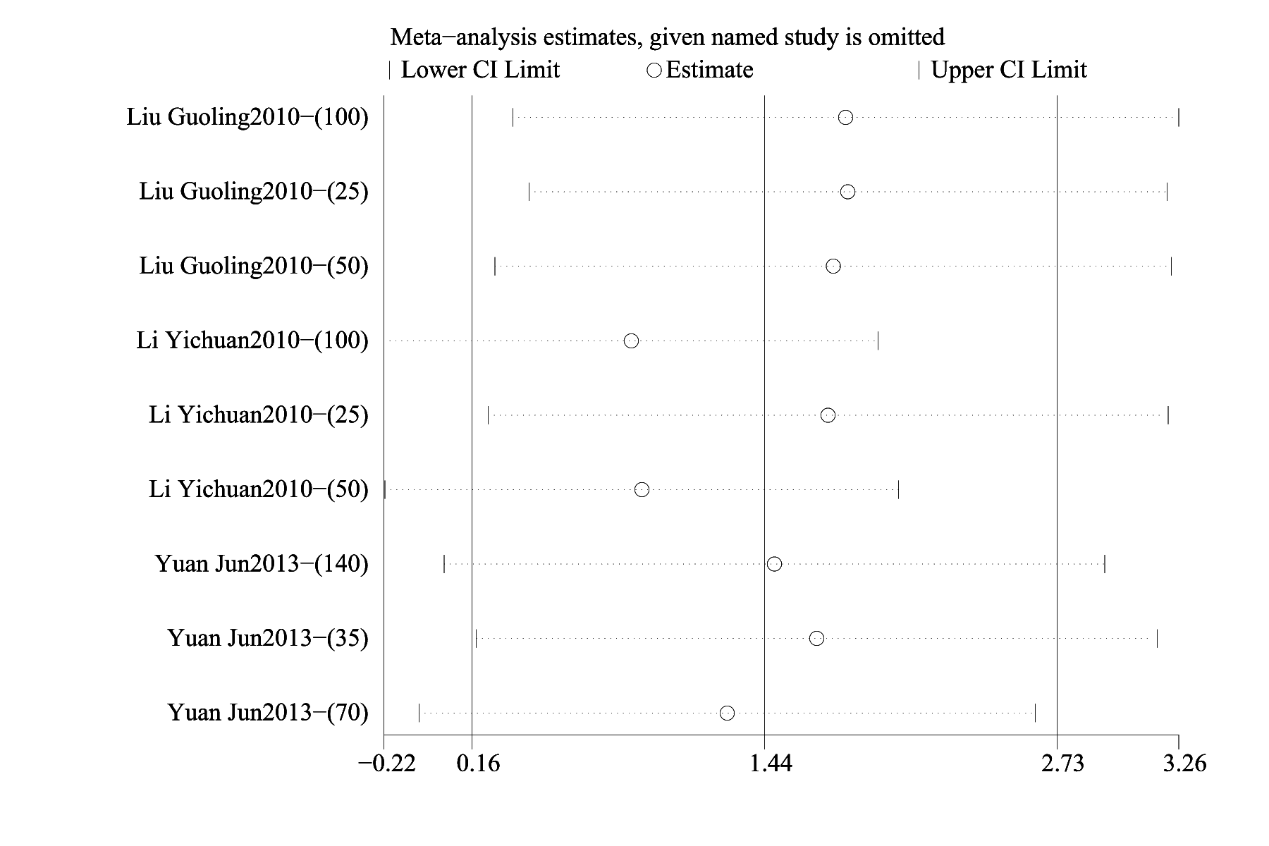


## 2.40 Subgroup analysis of IL-10 after 3-4 weeks of TGP intervention according to doses


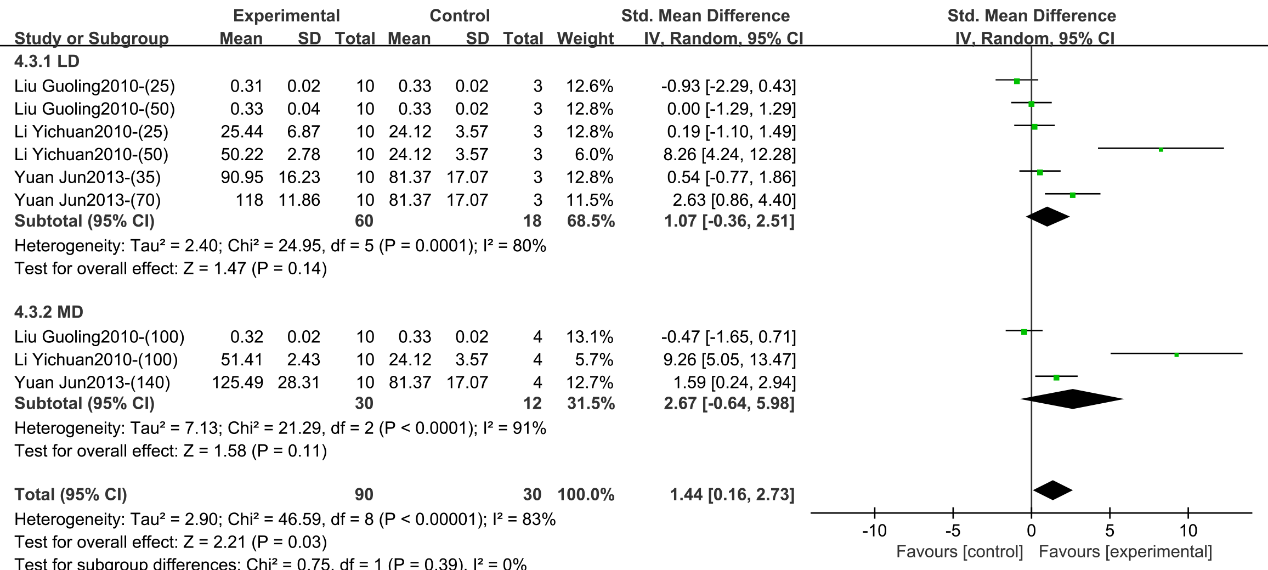


## 2.41 Subgroup analysis of IL-10 after 3-4 weeks of TGP intervention according to animal species


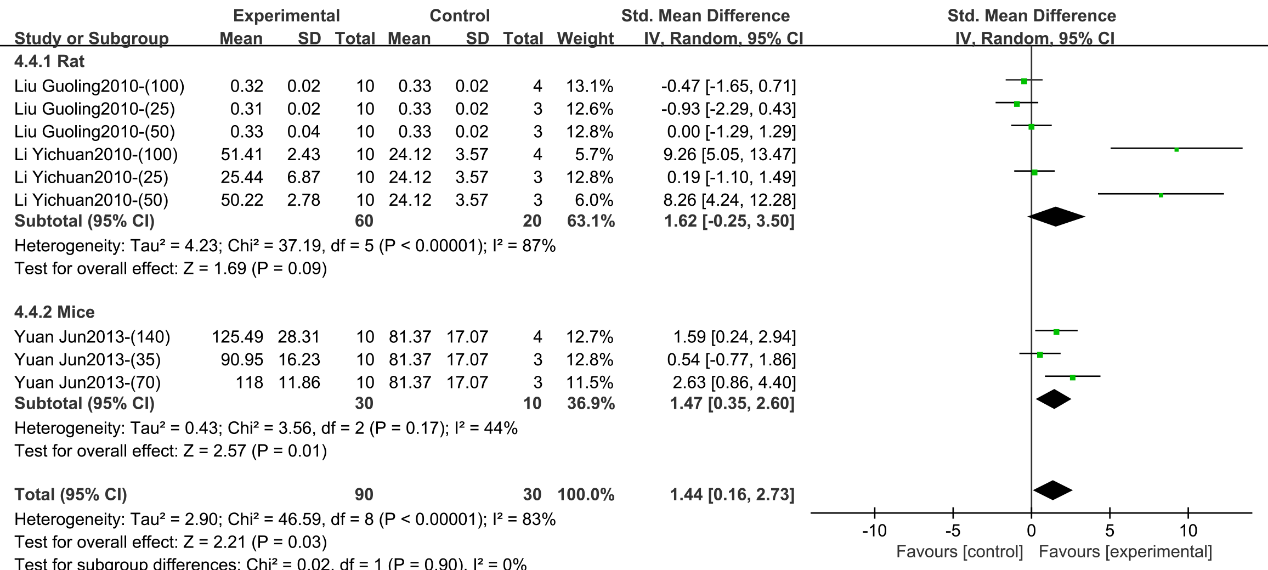


## 2.42 Subgroup analysis of IL-10 after 3-4 weeks of TGP intervention according to animal strains


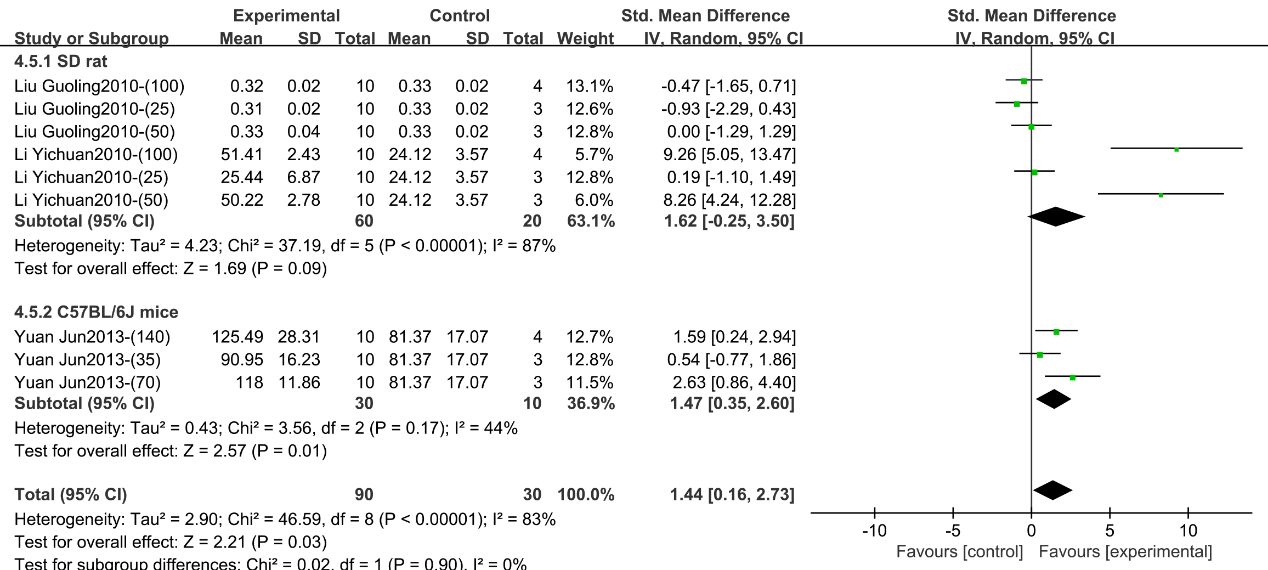


## 2.43 Forest plot of IL-10 after 8 weeks of TGP intervention


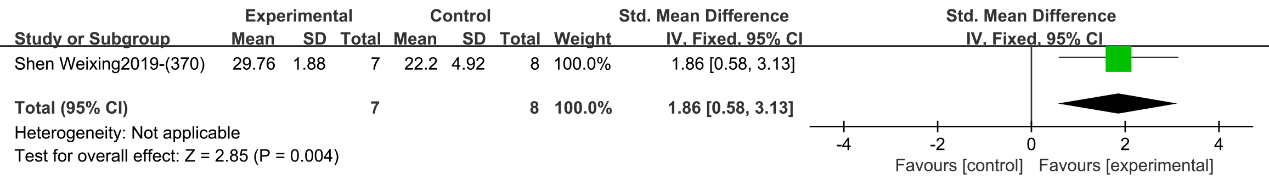


## 2.44 Forest plot of IL-1 after 3-4 weeks of TGP intervention


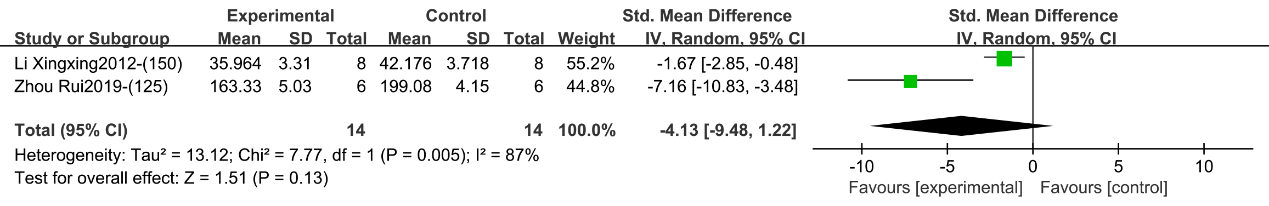


## 2.45 Forest plot of IL-2 after 3-4 weeks of TGP intervention


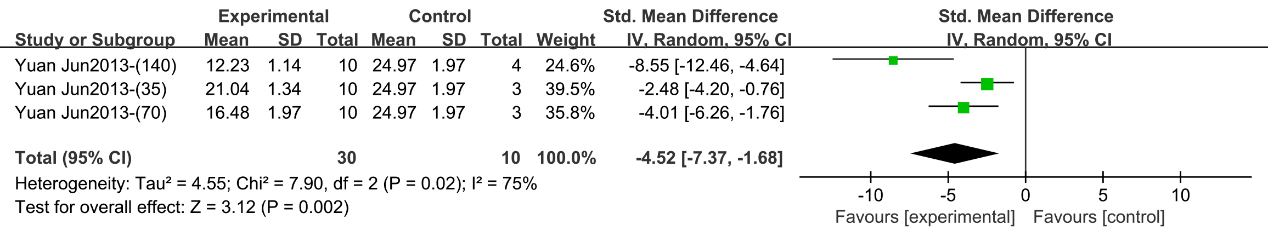


## 2.46 Sensitivity analysis of IL-2 after 3-4 weeks of TGP intervention


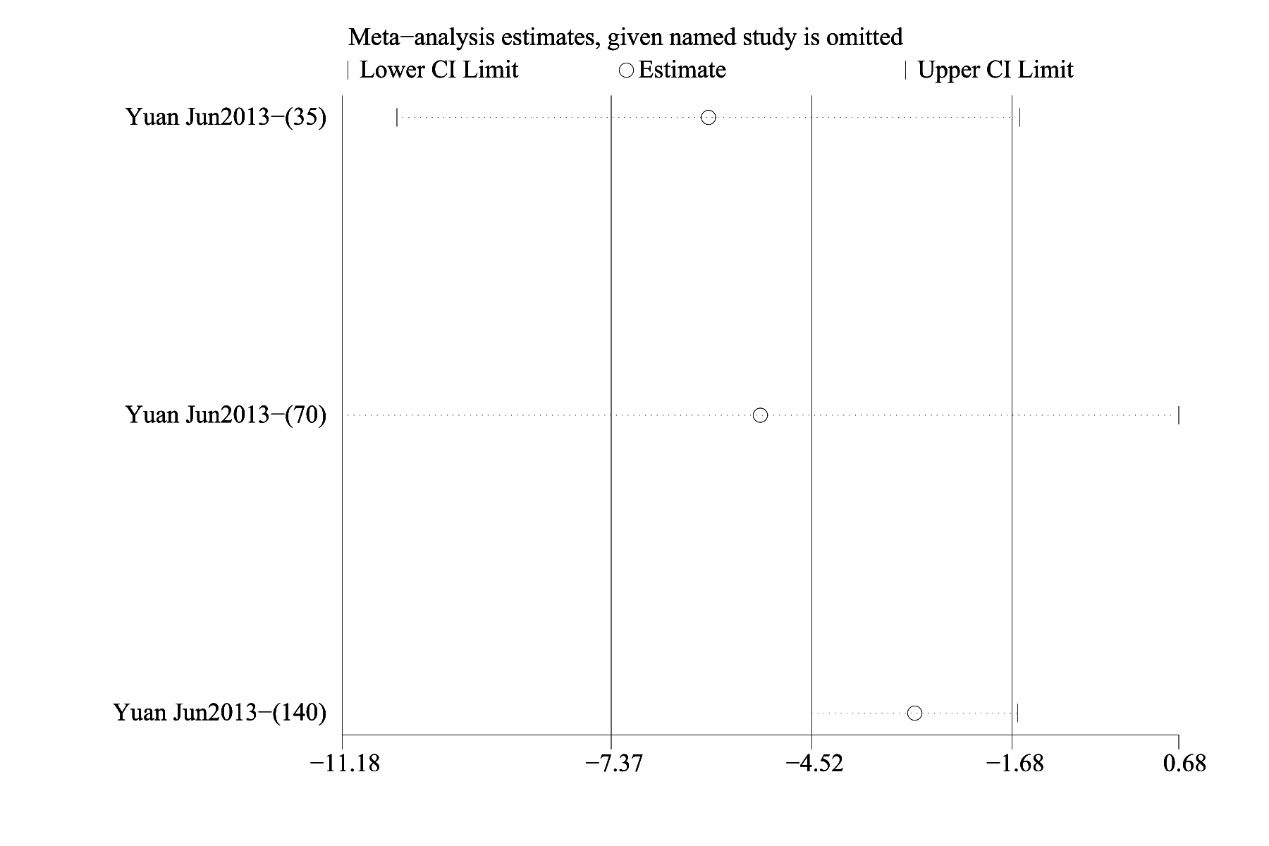


## 2.47 Subgroup analysis of IL-2 after 3-4 weeks of TGP intervention according to doses


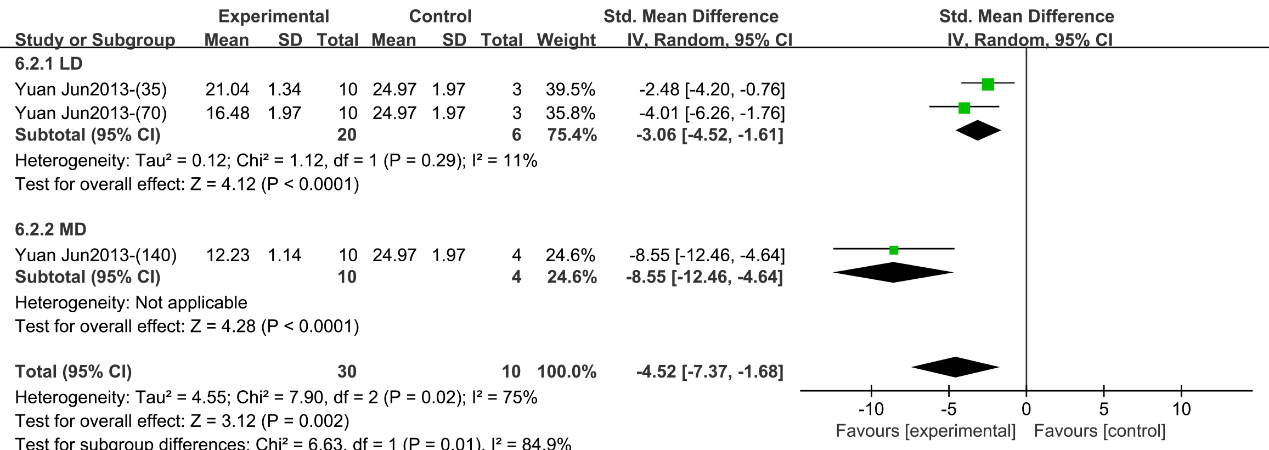


## 2.48 Forest plot of IL-4 after 3-4 weeks of TGP intervention


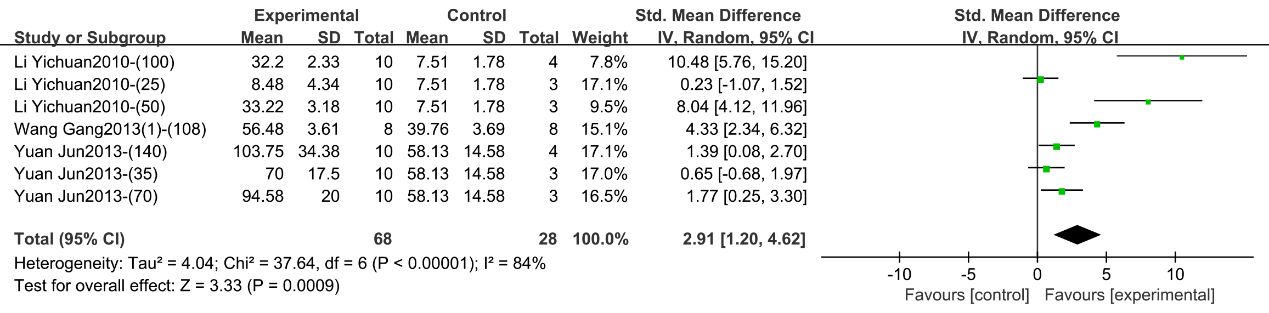


## 2.49 Sensitivity analysis of IL-4 after 3-4 weeks of TGP intervention


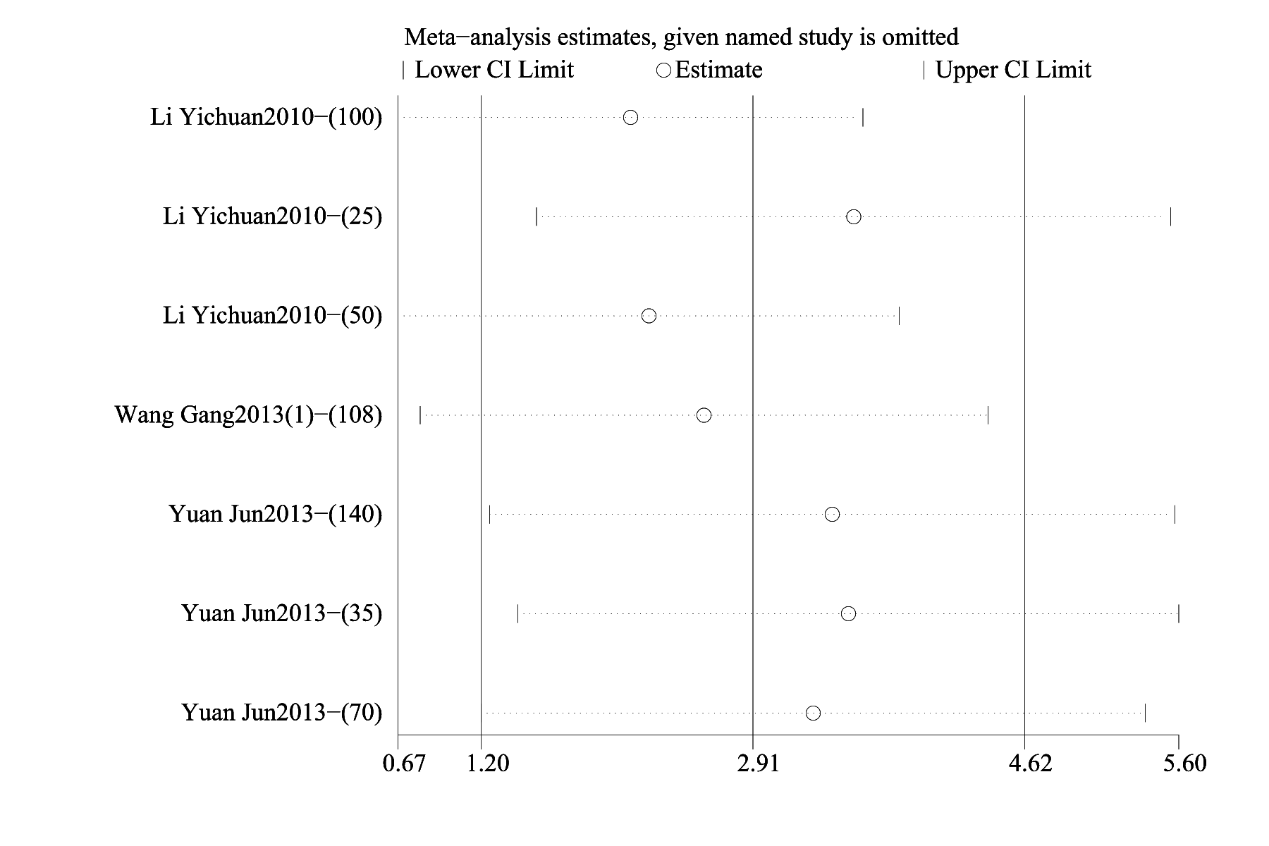


## 2.50 Subgroup analysis of IL-4 after 3-4 weeks of TGP intervention according to doses


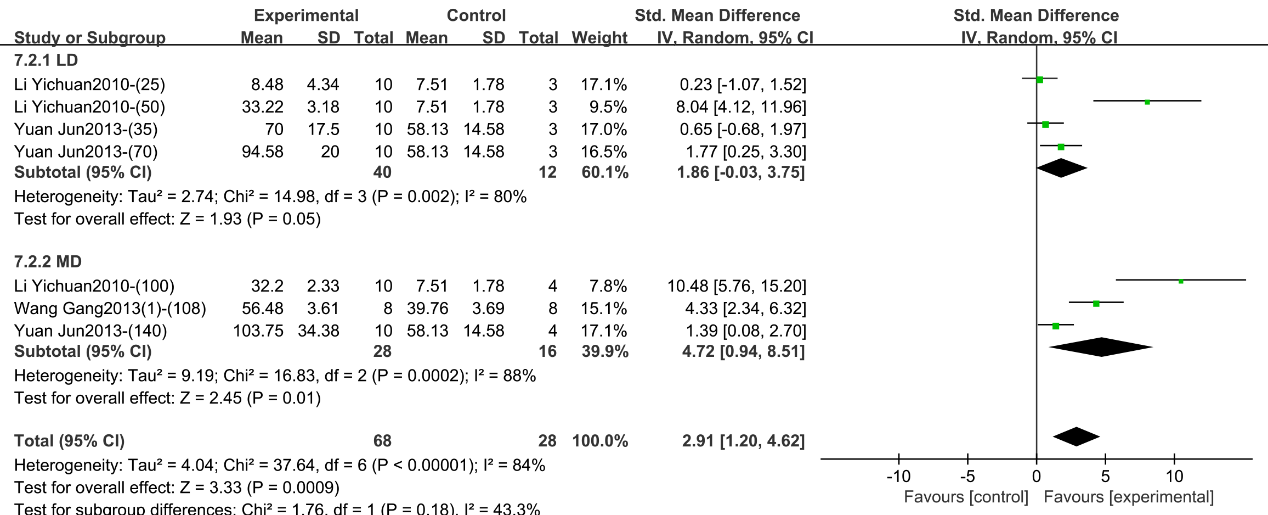


## 2.51 Subgroup analysis of IL-4 after 3-4 weeks of TGP intervention according to animal species


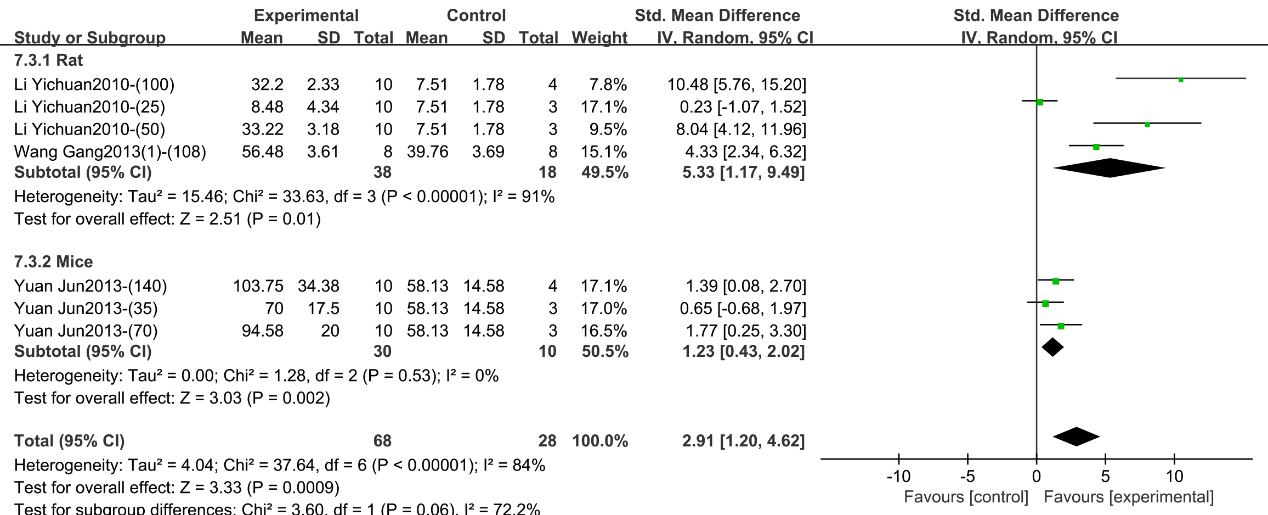


## 2.52 Subgroup analysis of IL-4 after 3-4 weeks of TGP intervention according to animal strains


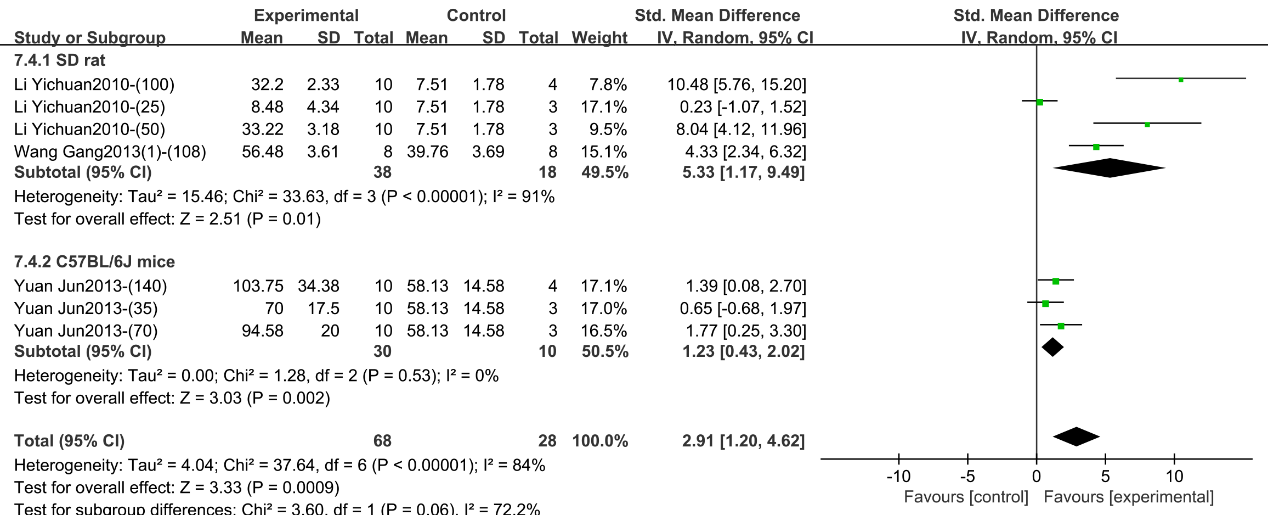


## 2.53 Subgroup analysis of IL-4 after 3-4 weeks of TGP intervention according to TGP 's manufacturers


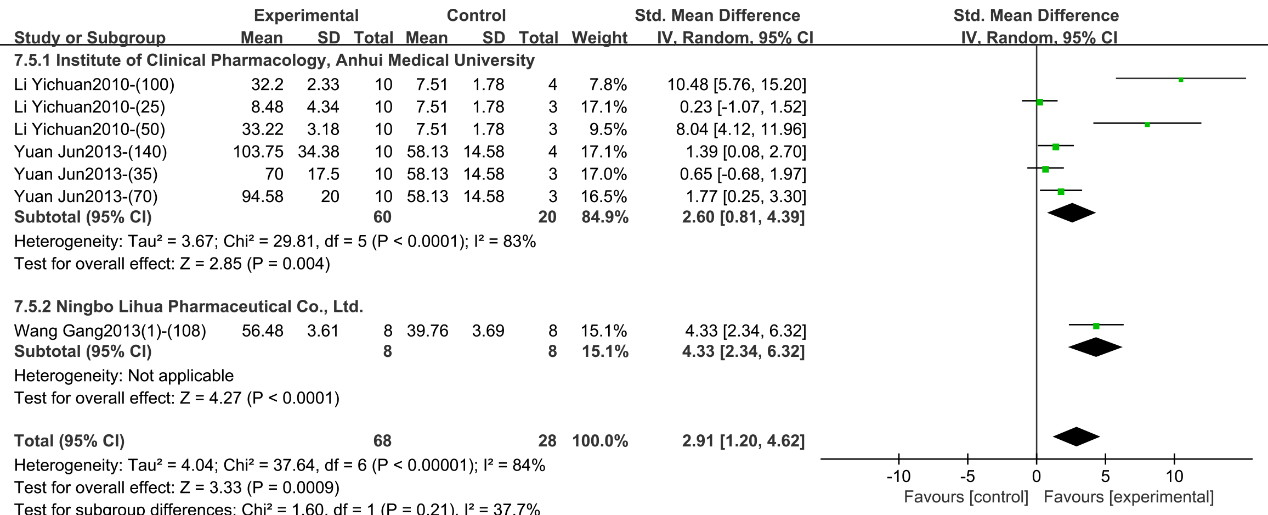


## 2.54 Forest plot of IL-17 after 1-2 weeks of TGP intervention


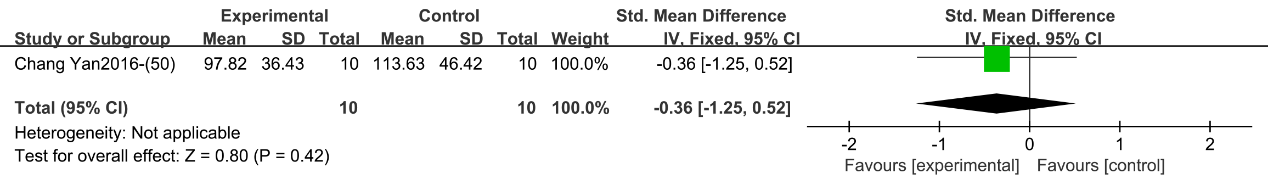


## 2.55 Forest plot of IL-17 after 3-4 weeks of TGP intervention


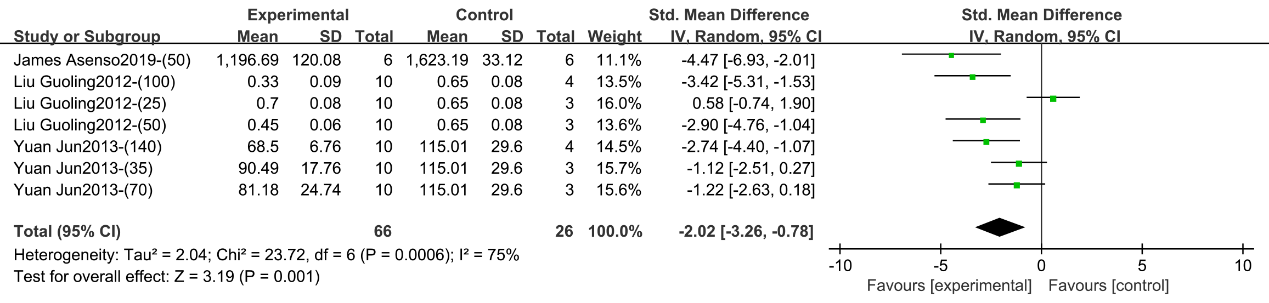


## 2.56 Sensitivity analysis of IL-17 after 3-4 weeks of TGP intervention


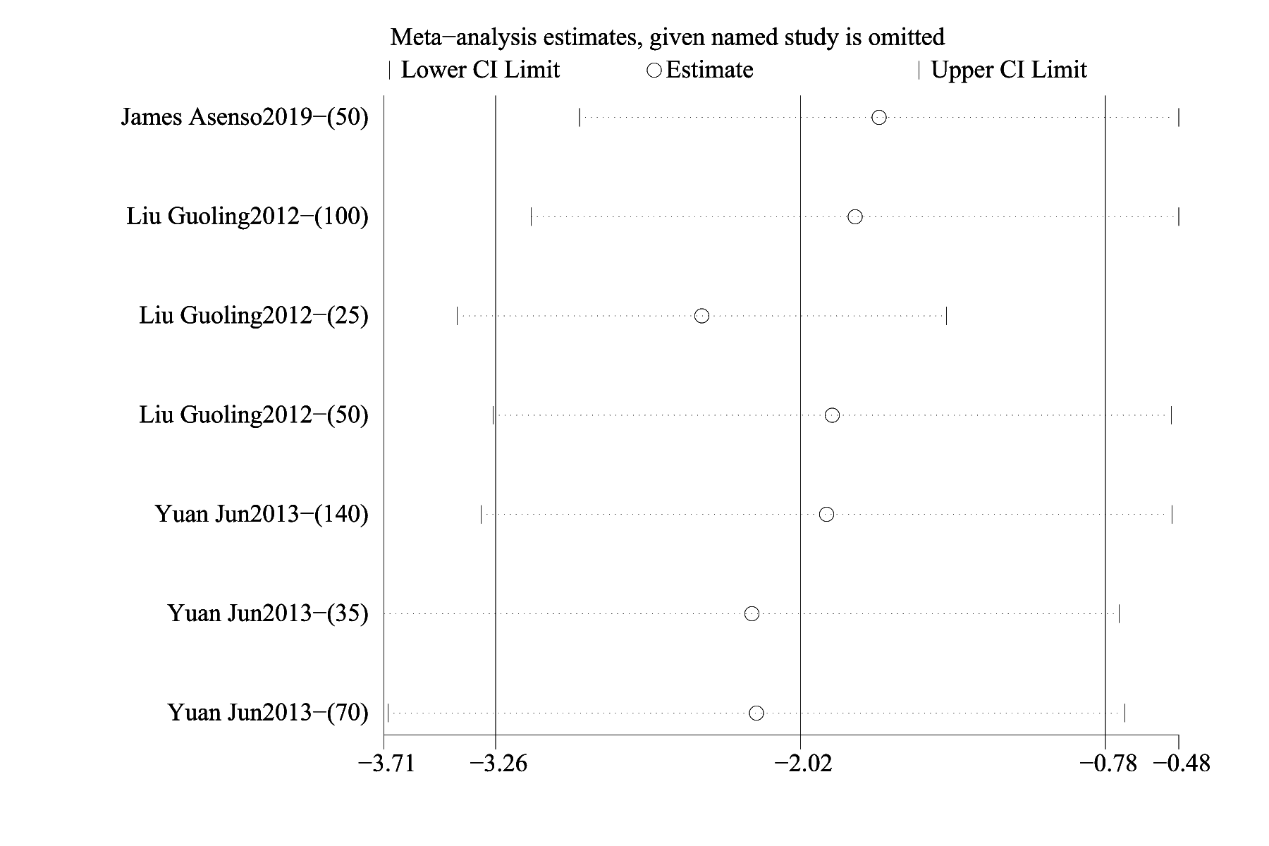


## 2.57 Subgroup analysis of IL-17 after 3-4 weeks of TGP intervention according to doses


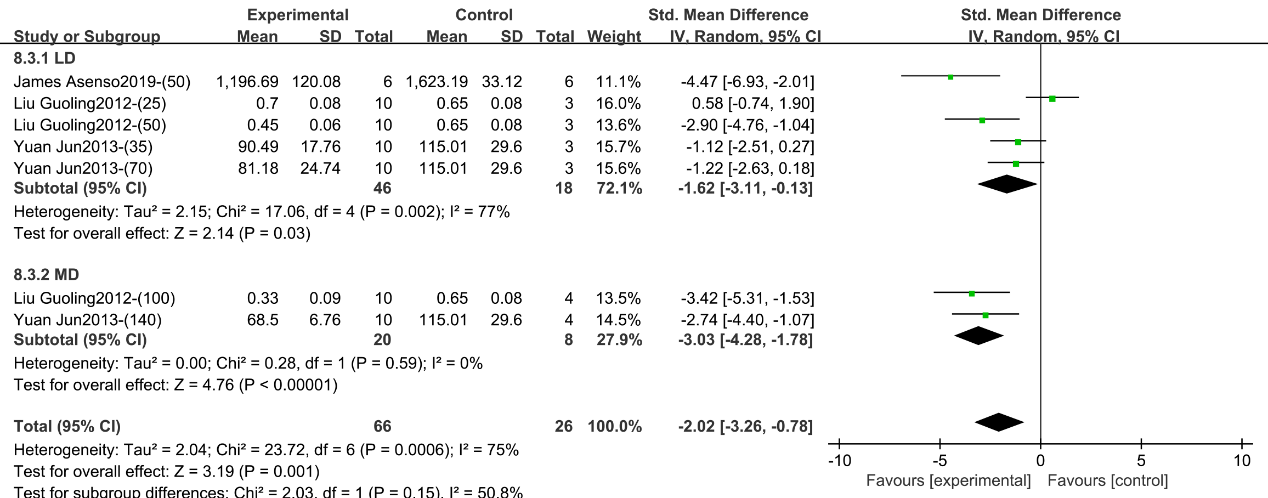


## 2.58 Subgroup analysis of IL-17 after 3-4 weeks of TGP intervention according to animal model types


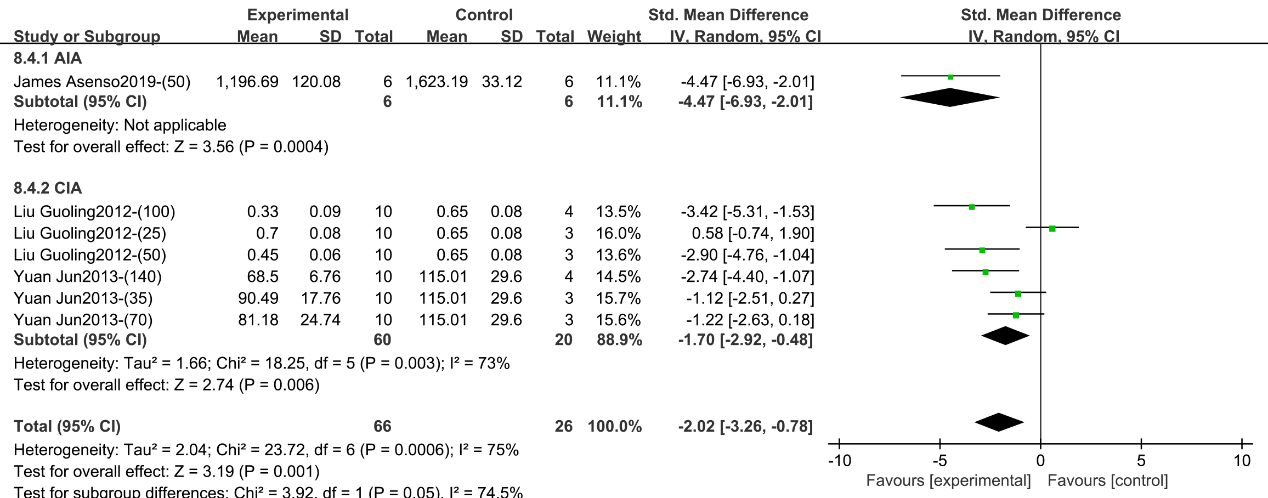


## 2.59 Subgroup analysis of IL-17 after 3-4 weeks of TGP intervention according to animal species


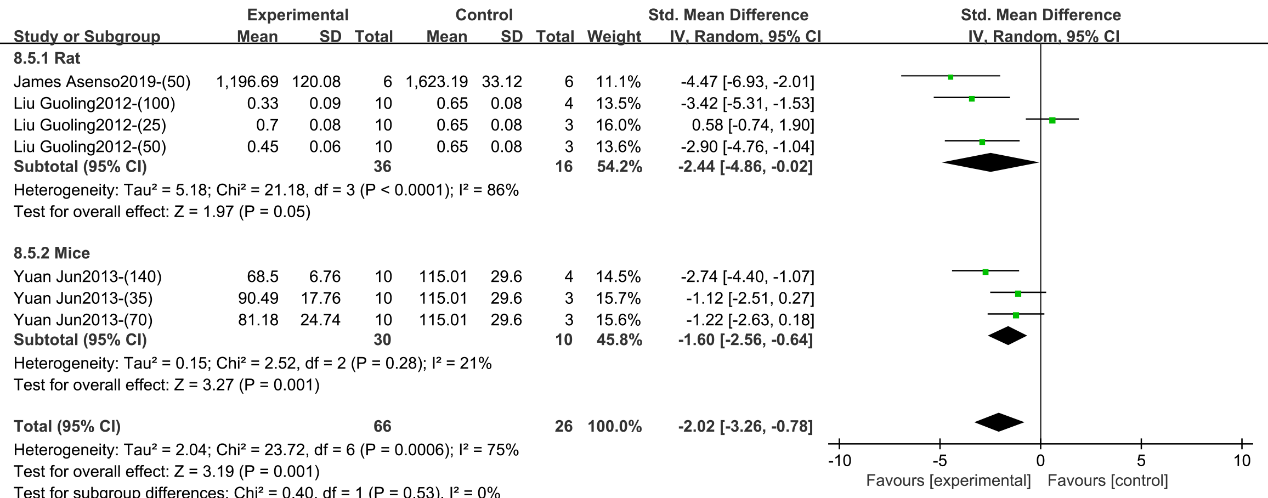


## 2.60 Subgroup analysis of IL-17 after 3-4 weeks of TGP intervention according to animal strains


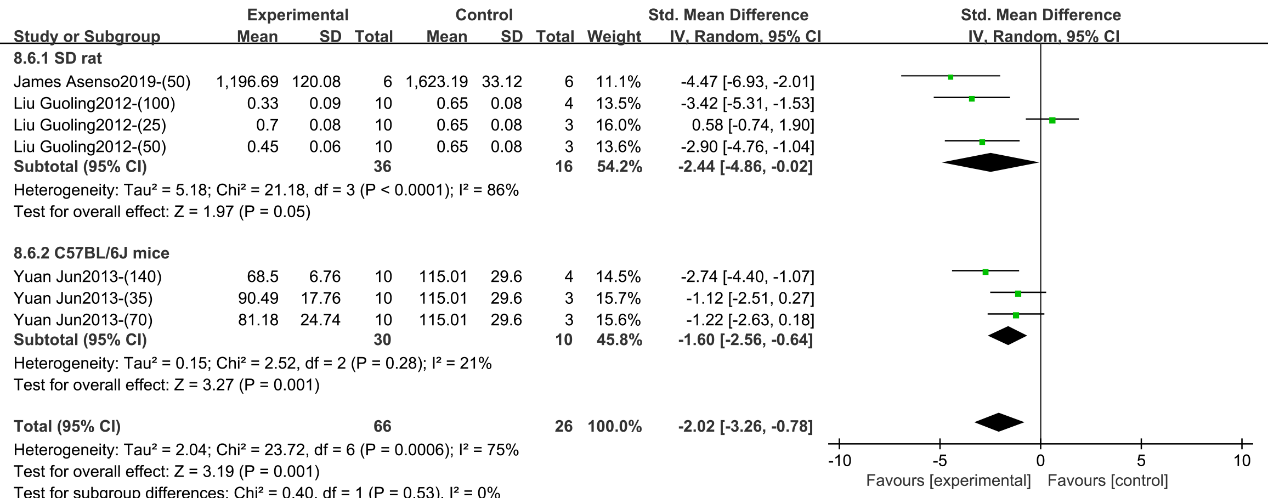


## 2.61 Forest plot of IL-17α after 3-4 weeks of TGP intervention


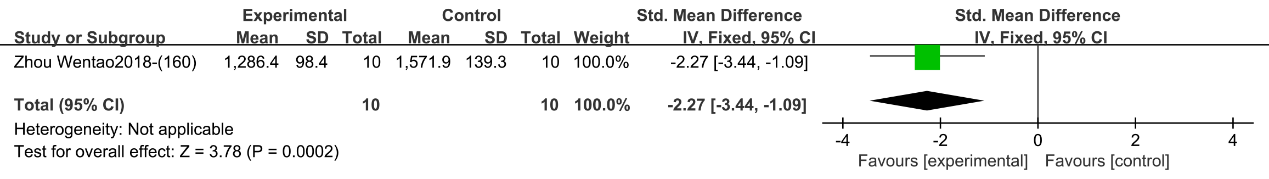


## 2.62 Forest plot of IL-17α after 8 weeks of TGP intervention


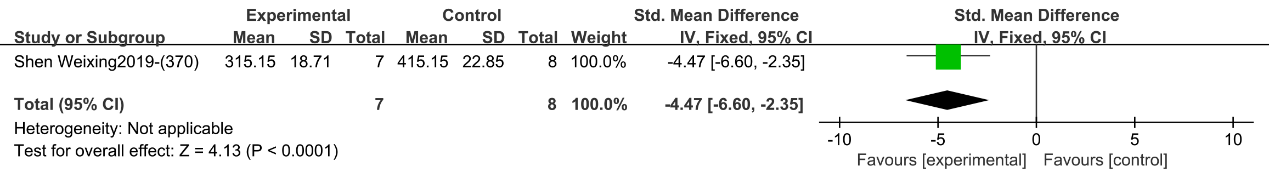


## 2.63 Forest plot of IL-21 after 3-4 weeks of TGP intervention


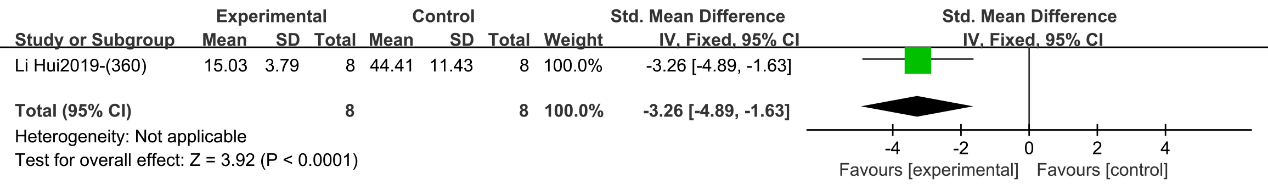


## 2.64 Forest plot of VEGF after 3-4 weeks of TGP intervention


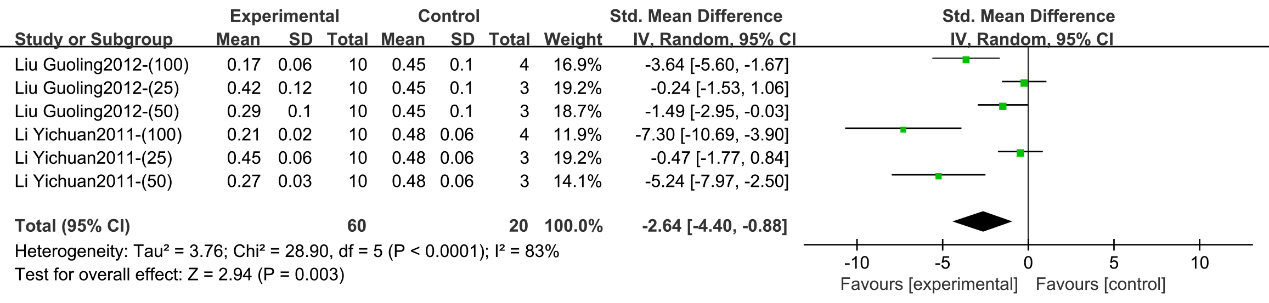


## 2.65 Sensitivity analysis of VEGF after 3-4 weeks of TGP intervention


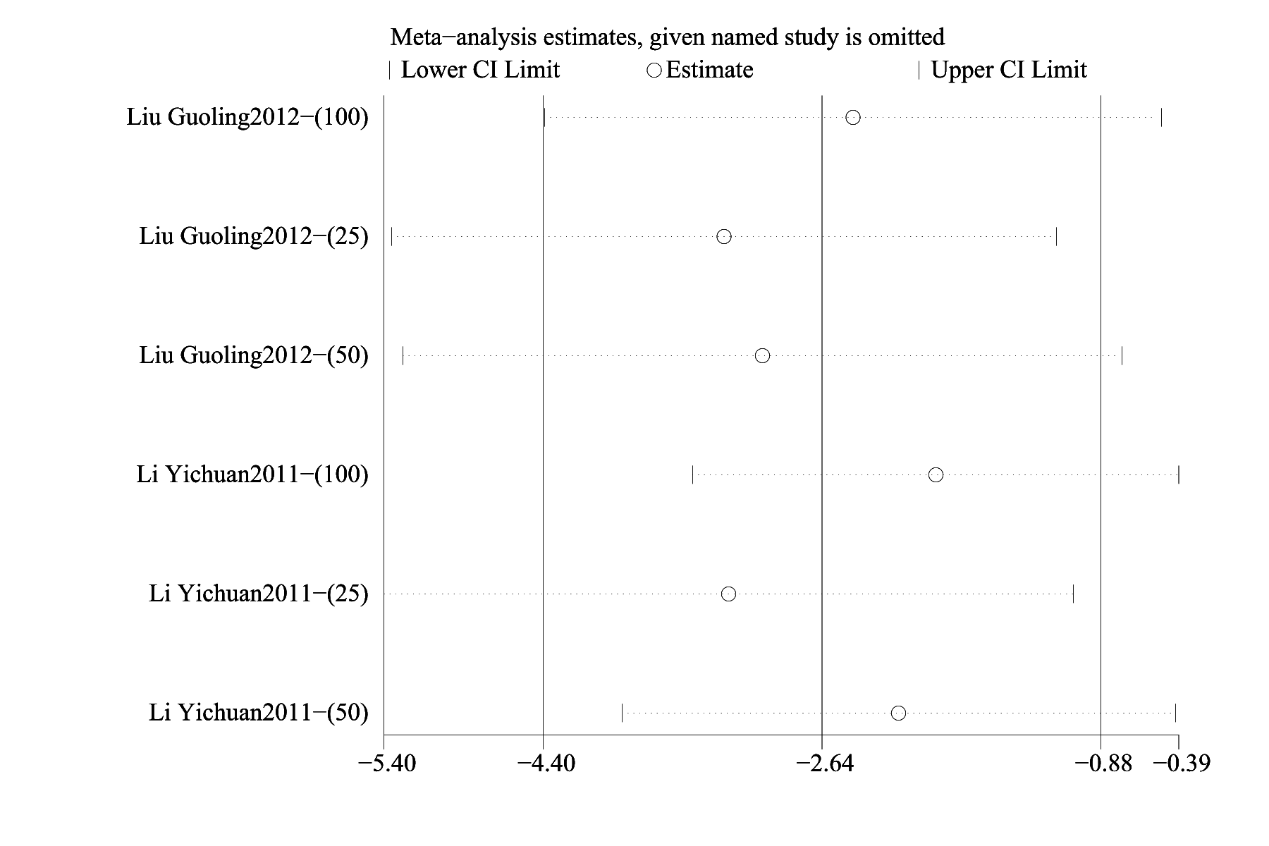


## 2.66 Subgroup analysis of VEGF after 3-4 weeks of TGP intervention according to doses


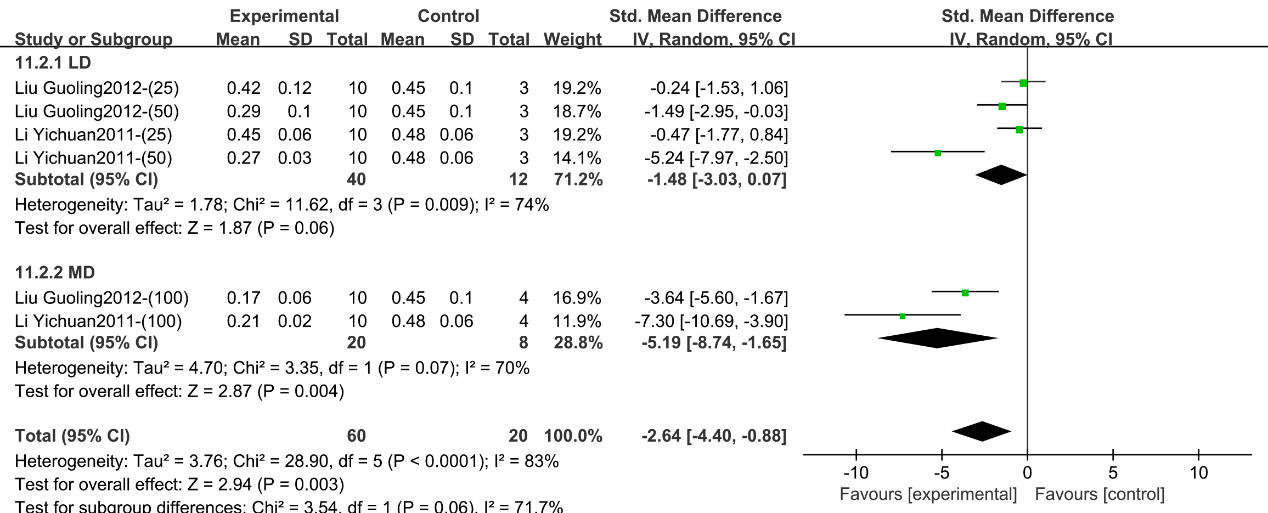


## 2.67 Forest plot of IFN-γ after 1-2 weeks of TGP intervention


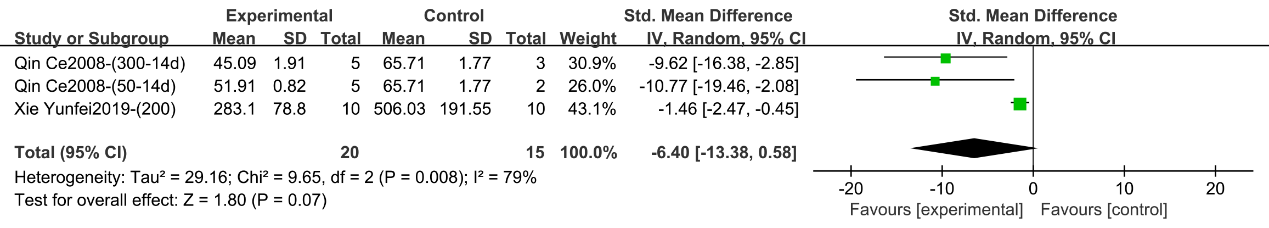


## 2.68 Sensitivity analysis of IFN-γ after 1-2 weeks of TGP intervention


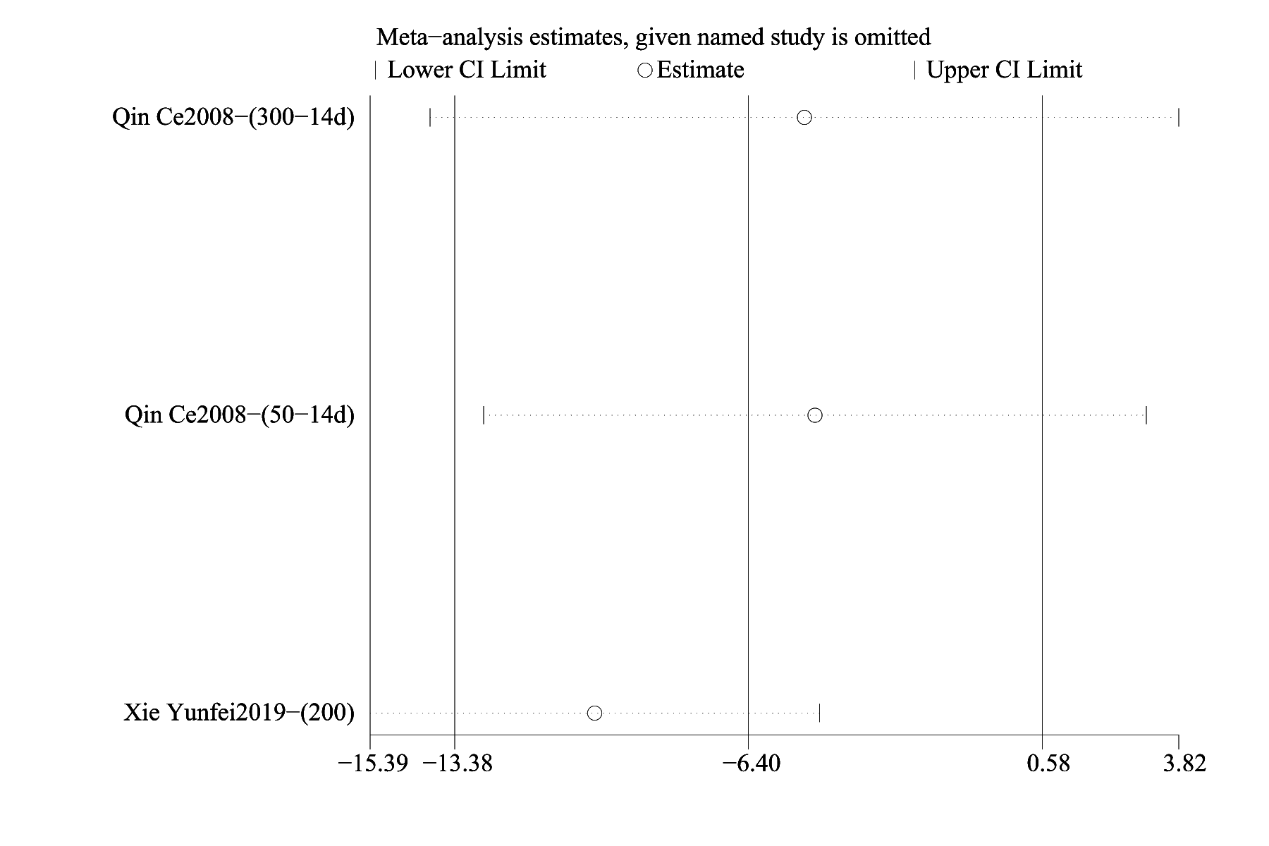


## 2.69 Subgroup analysis of IFN-γ after 1-2 weeks of TGP intervention according to doses


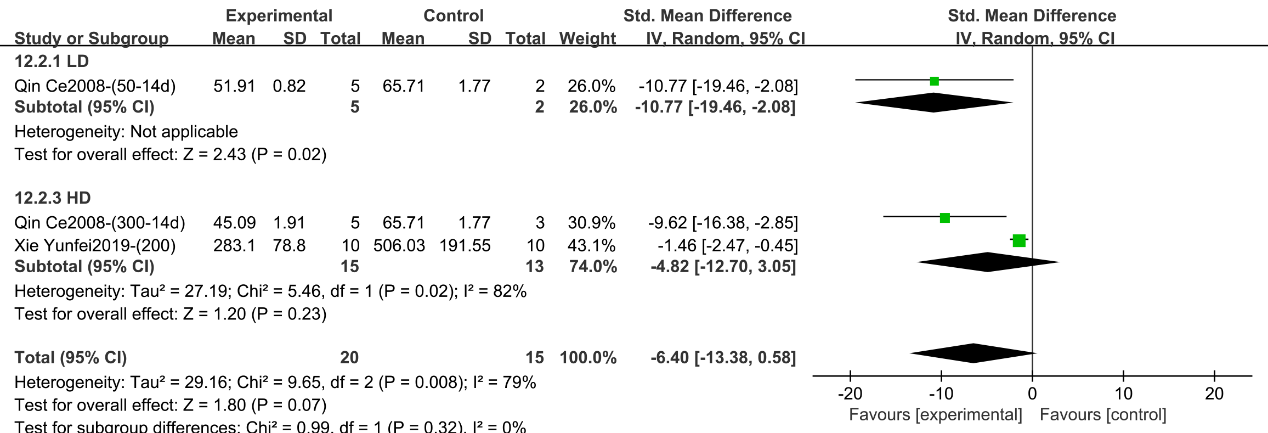


## 2.70 Subgroup analysis of IFN-γ after 1-2 weeks of TGP intervention according to animal strains


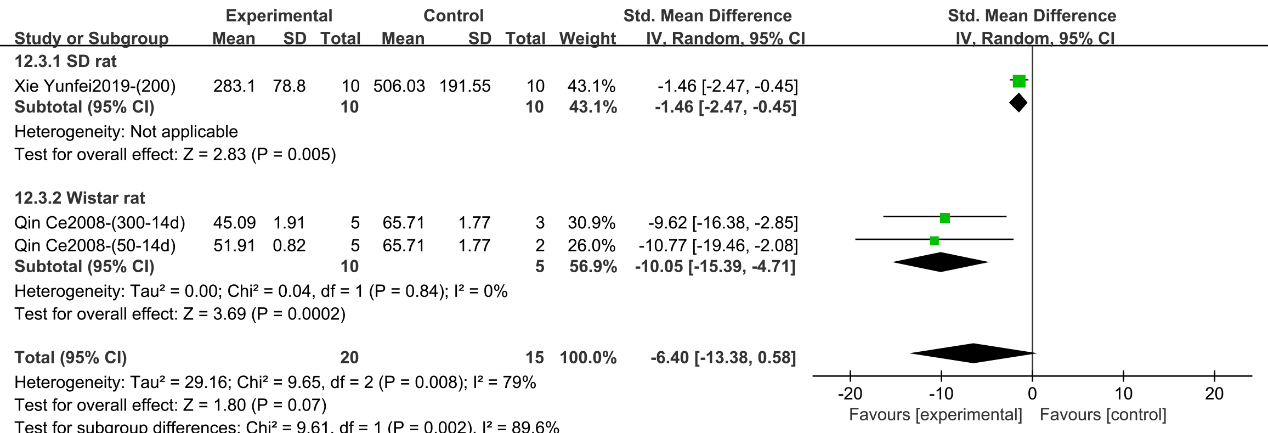


## 2.71 Subgroup analysis of IFN-γ after 1-2 weeks of TGP intervention according to TGP 's manufacturers


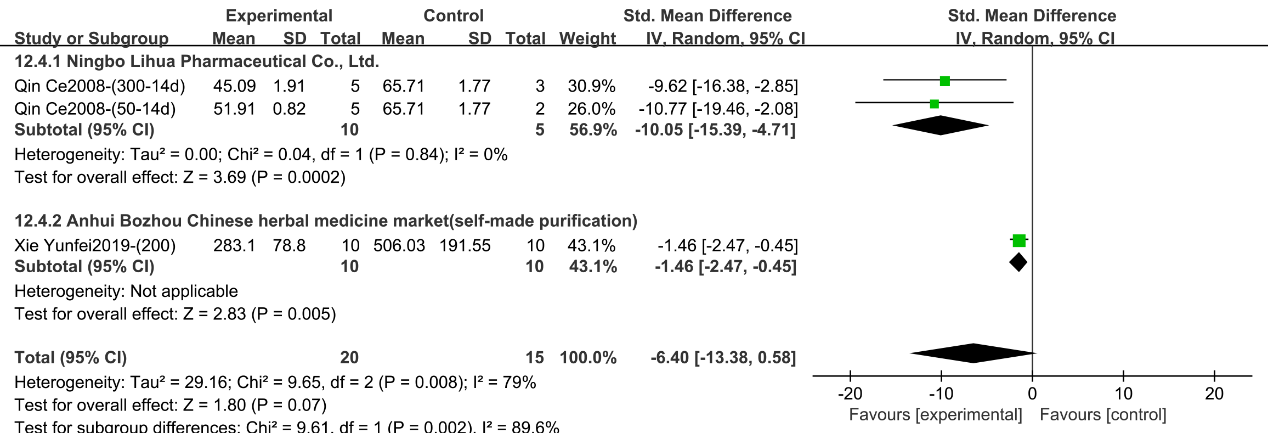


## 2.72 Forest plot of IFN-γ after 3-4 weeks of TGP intervention


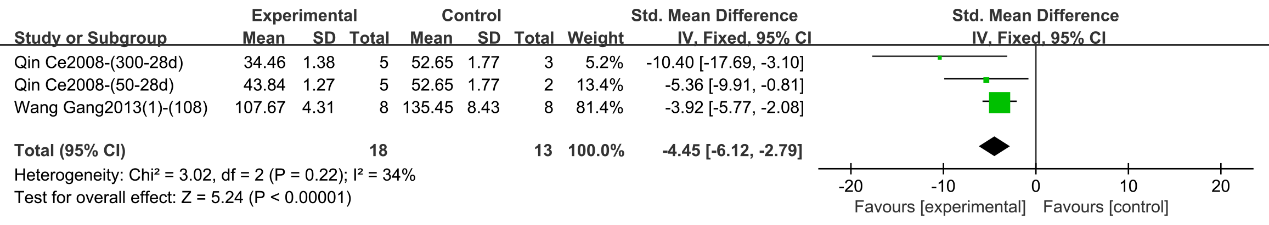


## 2.73 Forest plot of PGE2 after 1-2 weeks of TGP intervention


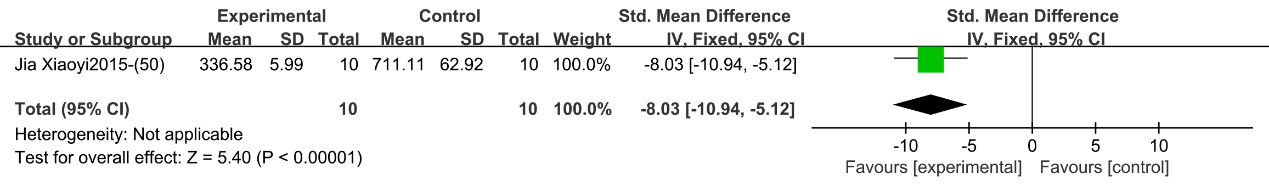


## 2.74 Forest plot of PGE2 after 3-4 weeks of TGP intervention


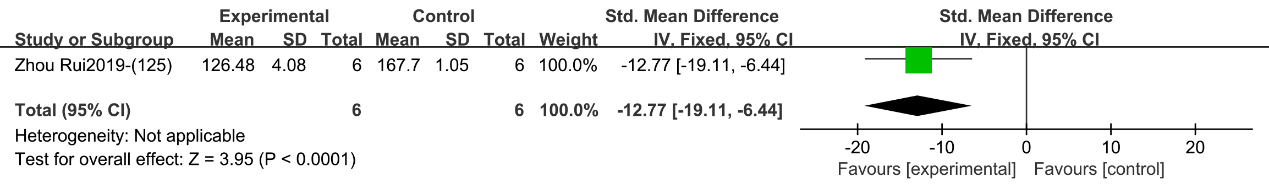


## 2.75 Forest plot of TGF-β1 after 1-2 weeks of TGP intervention


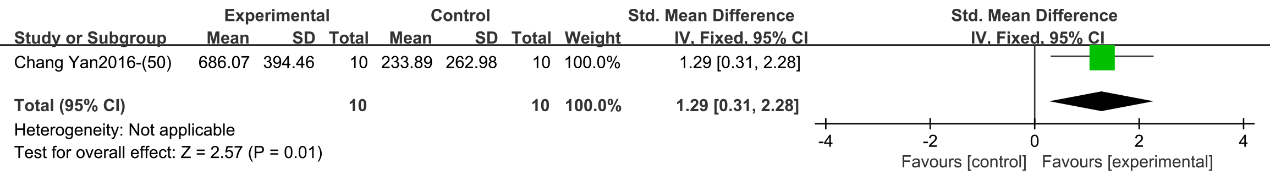


## 2.76 Forest plot of TGF-β1 after 3-4 weeks of TGP intervention


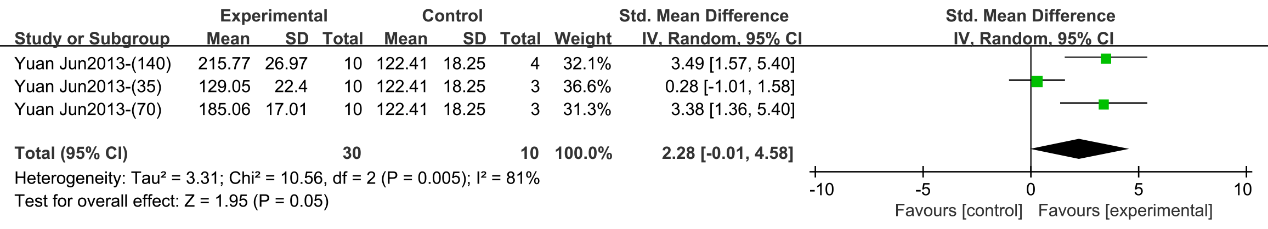


## 2.77 Sensitivity analysis of TGF-β1 after 3-4 weeks of TGP intervention


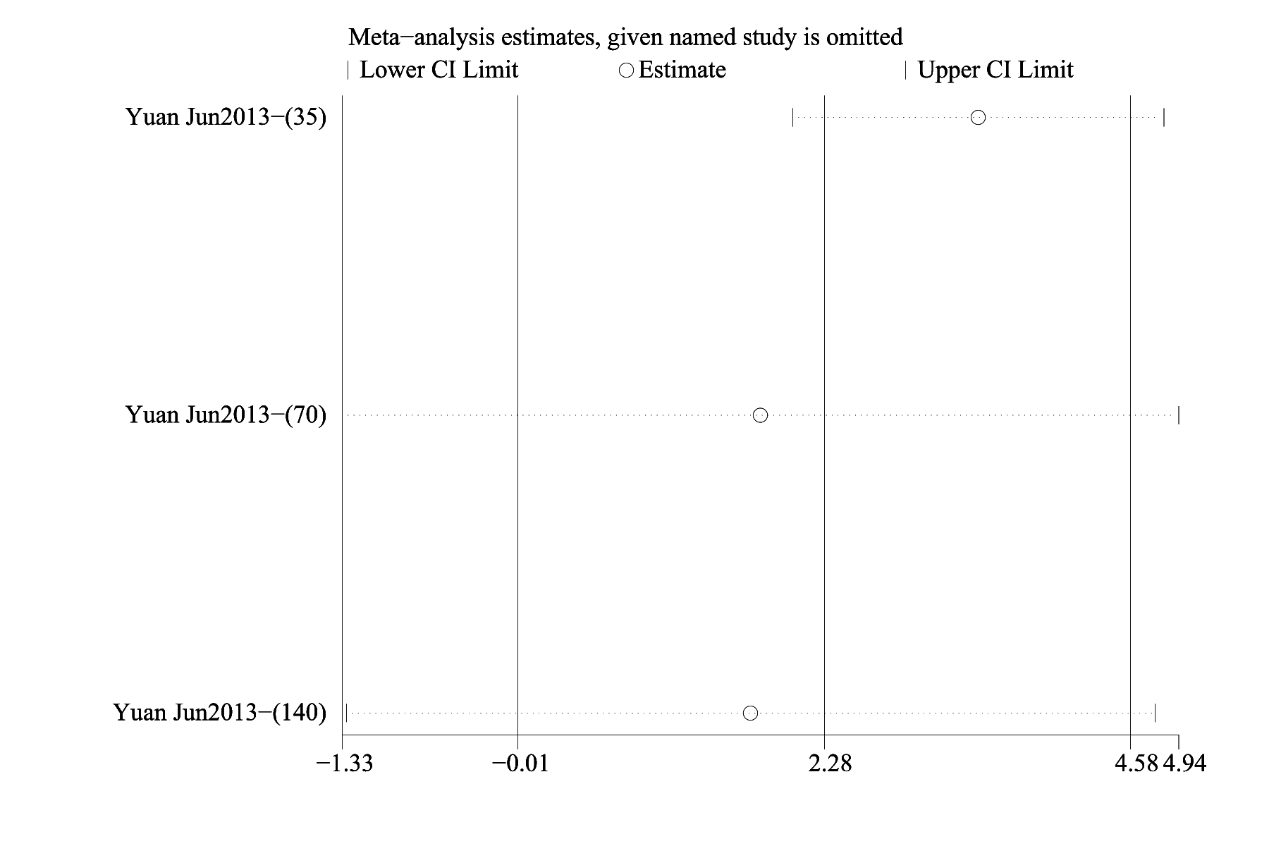


## 2.78 Subgroup analysis of TGF-β1 after 3-4 weeks of TGP intervention according to doses


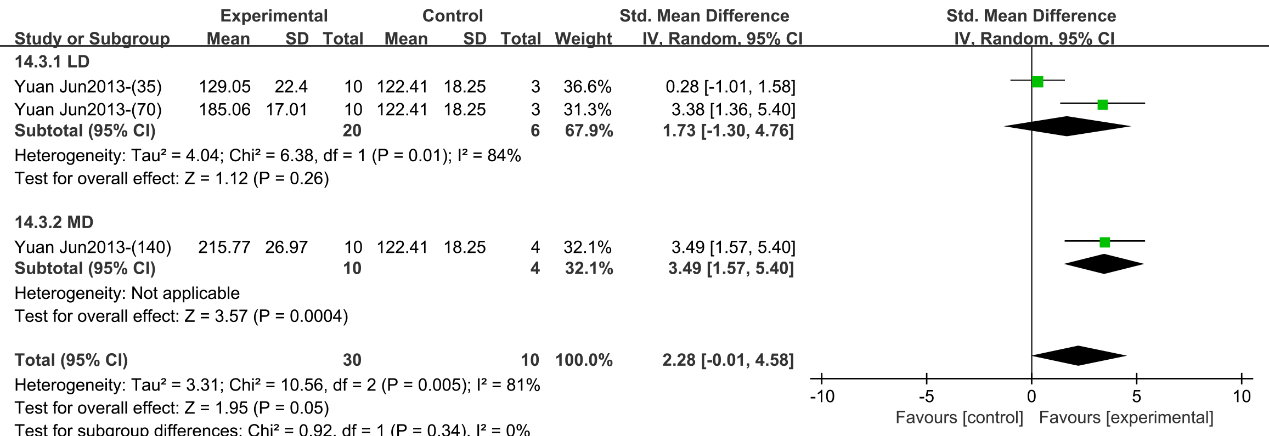

Supplement: Supplementary file 1 [file DataSheet1.docx]
